# Graphlet-based hyperbolic embeddings capture evolutionary dynamics in genetic networks

**DOI:** 10.1093/bioinformatics/btae650

**Published:** 2024-11-04

**Authors:** Sam F L Windels, Daniel Tello Velasco, Mikhail Rotkevich, Noël Malod-Dognin, Nataša Pržulj

**Affiliations:** Barcelona Supercomputing Center, Barcelona 08034, Spain; Barcelona Supercomputing Center, Barcelona 08034, Spain; Universitat de Barcelona, Barcelona 08007, Spain; Barcelona Supercomputing Center, Barcelona 08034, Spain; Universitat Politècnica de Catalunya, Barcelona 08034, Spain; Barcelona Supercomputing Center, Barcelona 08034, Spain; Barcelona Supercomputing Center, Barcelona 08034, Spain; ICREA, Barcelona 08010, Spain; Department of Computer Science, University College London, London WC1E 6BT, United Kingdom

## Abstract

**Motivation:**

Spatial Analysis of Functional Enrichment (SAFE) is a popular tool for biologists to investigate the functional organization of biological networks via highly intuitive 2D functional maps. To create these maps, SAFE uses Spring embedding to project a given network into a 2D space in which nodes connected in the network are near each other in space. However, many biological networks are scale-free, containing highly connected hub nodes. Because Spring embedding fails to separate hub nodes, it provides uninformative embeddings that resemble a ‘hairball’. In addition, Spring embedding only captures direct node connectivity in the network and does not consider higher-order node wiring patterns, which are best captured by graphlets, small, connected, nonisomorphic, induced subgraphs. The scale-free structure of biological networks is hypothesized to stem from an underlying low-dimensional hyperbolic geometry, which novel hyperbolic embedding methods try to uncover. These include coalescent embedding, which projects a network onto a 2D disk.

**Results:**

To better capture the functional organization of scale-free biological networks, whilst also going beyond simple direct connectivity patterns, we introduce Graphlet Coalescent (GraCoal) embedding, which embeds nodes nearby on a disk if they frequently co-occur on a given graphlet together. We use GraCoal to extend SAFE-based network analysis. Through SAFE-enabled enrichment analysis, we show that GraCoal outperforms graphlet-based Spring embedding in capturing the functional organization of the genetic interaction networks of fruit fly, budding yeast, fission yeast and *Escherichia coli*. We show that depending on the underlying graphlet, GraCoal embeddings capture different topology-function relationships. We show that triangle-based GraCoal embedding captures functional redundancies between paralogs.

**Availability and implementation:**

https://gitlab.bsc.es/swindels/gracoal_embedding.

## 1 Introduction

Driven by biotechnological advances, omics data is becoming increasingly abundant. These data are usually modelled as networks. For instance, in genetic interaction (GI) networks, nodes represent genes and edges connect two nodes if they *genetically interact*: the genes’ concurrent mutation changes a cell’s phenotype more than expected from their individual mutants if the genes were independent (Ashworth *et al.* 2011). An example is *synthetic lethality*, where the co-occurrence of two individually nonlethal gene mutations results in cellular death (Ashworth *et al.* 2011). In protein–protein interaction (PPI) networks, nodes represent proteins and edges connect nodes (proteins) that can bind. The analysis of biological networks has facilitated the understanding of complex biological systems and diseases. For instance, GI network analysis has been used to uncover novel therapeutic targets by exploiting disease-specific synthetic lethality interactions in cancer (Mair *et al.* 2019) and SARS-CoV-2 (Mast *et al.* 2020).

### 1.1 Network embedding

Because of their increasing size, analyzing modern networks directly is becoming computationally intractable. Thus, to ease downstream analyses, modern methods first transform the network into a low-dimensional vector-based representation, so that nodes that are directly connected, i.e. that are in the same *neighbourhood*, have similar vector representations. This process is referred to as *network embedding* (Li *et al.* 2022). Knowledge is then extracted from these embeddings based on *guilt by association*: nodes that have similar embeddings, and thus occur in the same neighbourhood in the network, are assumed to be functionally associated. Although all network embedding methods capture neighbourhood information, there is some nuance in how they do so. For instance, *Spectral embedding* groups nodes in the embedding space so that there are as few edges as possible between the nodes belonging to different groups (Belkin and Niyogi 2003). *Spring embedding* aims to group connected nodes by imagining all nodes in the network to repel each-other, whilst the edges as act as springs that pull the connected nodes together (Fruchterman and Reingold 1991).

Although intuitive, when applied to biological networks, Spring embedding is likely to produce uninformative, entangled embeddings resembling a ‘hairball’ (Bläsius *et al.* 2021). This is because many biological networks, including PPI (Jeong *et al.* 2001) and GI networks (Tong *et al.* 2004), are *scale-free*: the probability of a given node to have d neighbours follows a power law: $P(d)∼d^{-\lambda }$, where λ usually ranges between 2 and 3 (Ravasz and Barabási 2003). This means that in scale-free networks there exist few nodes with many neighbours, i.e. that have a high *degree*. These so-called hub-nodes are also likely to be connected to each other because of the rich-get-richer principle. Consequentially, Spring embedding does not manage to spread the hub-nodes, as they are pulled together by the springs connecting them, leaving little room to embed their numerous low-degree neighbours (Bläsius *et al.* 2021).

It is hypothesized that the scale-freeness of biological networks stems from a low-dimensional underlying hyperbolic geometry (Boguñá *et al.* 2021). For instance, the latent hyperbolic geometry of the brain connectome is though to be 3D (Almagro *et al.* 2022). To uncover the latent hyperbolic geometry of scale-free networks, *hyperbolic embedding* methods embed a network into a hyperbolic space. For instance, *Coalescent embedding* (CE) maps nodes onto a disk so that nodes that cluster in the network are assigned a similar angle and so that nodes with a higher degree are embedded near the circle’s centre (Muscoloni *et al.* 2017). As the circumference of a disk increases exponentially by its radius, CE manages to embed scale-free networks without excessive node overlap. CE successfully detects communities in many real networks (Muscoloni *et al.* 2017) and the rewiring of brain networks in Parkinson’s disease (Cacciola *et al.* 2017).

### 1.2 Graphlet adjacency

The above methods above capture the neighbourhood information based on standard *adjacency*, which considers two nodes to be adjacent (neighbours) if they are directly connected by an edge. Formally, if H is a network with a set of nodes V and a set of edges E, then two nodes, u∈V and v∈V, are *adjacent* if there is an edge (u,v)∈E. The connectivity of a network is usually represented in an *adjacency matrix*, $A^{\left|V|\times |V\right|}$, where the entry A(u,v) is 1 if u and v are adjacent and 0 otherwise.

**Figure 1.**
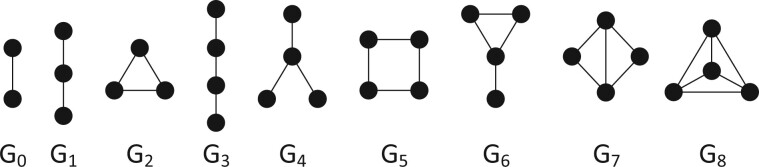
An illustration of all up to four-node graphlets ($G_{0}$–$G_{8}$).

Alternatively, information on a node can also be inferred from the number of times it touches different types of sub-graphs (e.g. triangles, paths …), known as the node’s wiring or *topology*. The state-of-the-art methods to quantify node topology are based on *graphlets*, small connected, nonisomorphic, induced sub-graphs, illustrated in Fig. 1 (Pržulj *et al.* 2004).

To simultaneously capture neighbourhood and topology information, we recently introduced *graphlet adjacency*, which quantifies the adjacency of two nodes based on how frequently they touch a given graphlet $G_{i}$ together in the network (Windels *et al.* 2019). The graphlet adjacency matrix is defined as:

(1) $$\begin{array}{c} A_{G_{i}}(u,v)=\{\begin{array}{cc} c_{uv}^{G_{i}}/\theta_{G_{i}} & \text{if}\;u\neq v\\ 0 & \text{otherwise}, \end{array} \end{array}$$

where $c_{uv}^{G_{i}}$ is equal to the number of times the nodes u and v simultaneously touch graphlet $G_{i}$ and $\theta_{G_{i}}$ is a scaling constant equal to the number of nodes in graphlet $G_{i}$ minus 1. We illustrate graphlet adjacency applied on a toy network in Supplementary Section S2.1. Note that the adjacency matrix for graphlet $G_{0}$, $A_{G_{0}}$, is the standard adjacency matrix, A. To achieve a more balanced clustering or evenly distributed embedding space, the graphlet adjacency matrix is usually normalized. The symmetrically normalized graphlet adjacency matrix, ${\tilde{A}}_{G_{i}}$, is defined as: ${\tilde{A}}_{G_{i}}=D_{G_{i}}^{1/2}A_{G_{i}}D_{G_{i}}^{1/2}$, where $D_{G_{i}}$ is the diagonal matrix such that $D_{G_{i}}(u,u)$ is the number of times node u touches graphlet $G_{i}$.

We used graphlet adjacency to define *Graphlet Spectral embedding* and *Graphlet Spectral clustering* (Windels *et al.* 2019). Through clustering enrichment analysis, we showed that graphlet adjacencies capture complementary biological functions in molecular networks.

### 1.3 Problem

Despite the abundance of omics networks, our knowledge of their functional organization remains incomplete. A state-of-the-art algorithm to describe the functional organization of a network is Spatial Analysis of Functional Enrichment (SAFE) (Baryshnikova 2018). Given a network and a set of node annotations, SAFE applies 2D Spring embedding to uncover network neighbourhoods where node annotations are over-represented or *enriched*. The annotations enriched in the same network neighbourhood are aggregated into larger *domains* and highlighted as coloured regions in the Spring embedding. In this way, SAFE creates an intuitive functional map of a network, enabling the study of its functional organization. For instance, Costanzo *et al.* used SAFE to show that the GI network of budding yeast is organized in hierarchical modules (Costanzo *et al.* 2016). Rauscher *et al.* applied SAFE on GI data for human cancer cells to uncover their functional rewiring, identifying new genotype-specific vulnerabilities of cancer cells. (Rauscher *et al.* 2018).

However, SAFE relies on Spring embedding, which provides relatively uninformative embeddings when applied on scale-free networks (Bläsius *et al.* 2021). Many biological networks are scale-free, including GI and PPI networks. In addition, SAFE only considers standard adjacency, thus ignoring the information hidden in biological networks’ higher-order wiring.

### 1.4 Contribution

To better capture the functional organization of scale-free networks, whilst also taking into account graphlet-based wiring patterns, we introduce Graphlet Coalescent (GraCoal) embedding. For a given graphlet, GraCoal embedding maps a network onto a disk so that: (i) nodes that tend to be frequently connected by that graphlet are assigned a similar angle, and (ii) so that nodes with high counts of that graphlet are near the disks’ centre. We leverage GraCoal embedding’s low-dimensional nature by using it to extend SAFE-based network analysis. We apply our method to study the functional organization of molecular networks. To enable a complete comparison with the original SAFE, which is based on Spring embedding, we generalize Spring embedding to Graphlet Spring (GraSpring) embedding, in which the tension on the springs is set based on graphlet adjacency. We also compare against Graphlet Spectral embedding as it underlies GraCoal embedding.

Through SAFE enabled enrichment analysis, we show that GraCoal embeddings better capture the functional organization of the GI networks of fruit fly, budding yeast, fission yeast and *Escherichia coli* than GraSpring and Graphlet Spectral embedding. Moreover, we find that GraCoal embeddings capture different topology-function relationships. In addition, the best performing GraCoal depends on the species: either triangle-based GraCoal embeddings or GraCoal embeddings void of triangles tend to best capture the functional organization of GI networks. We explain this result by showing that triangle-based GraCoal embeddings capture the functional redundancy of paralogous (i.e. duplicated) genes. Hence, in species with many paralogs, this leads to high enrichment scores for triangle-based Gracoal embeddings.

## 2 Materials and methods

### 2.1 Data

#### 2.1.1 Omics network data

We collect the GI network data from BioGRID v.3.5.177 for *Saccharomyces cerevisiae* (budding yeast), *Schizosaccharomyces pombe* (fission yeast), *Drosophila melanogaster* (fruit fly), and *E. coli* (Oughtred *et al.* 2019). We collect the PPI networks from BioGRID for those same four species and two additional ones: *Caenorhabditis elegans* (nematode worm), and *Mus musculus* (mouse) (Oughtred *et al.* 2019). We collect the GI similarity network for *S. cerevisiae* (Costanzo *et al.* 2010, 2016). For the numbers of nodes and edges of these networks, see Supplementary Section S1.1.

#### 2.1.2 Gene functional annotation data

We collect the experimentally validated annotations from the Gene Ontology (i.e. evidence codes EXP, IDA, IPI, IMP, IGI, and IEP), which assign genes to biological process annotations (GO-BP), cellular component annotations (GO-CC), and molecular function annotations (GO-MF) (The Gene Ontology Consortium 2021). For the numbers of each of these annotations and the numbers of genes they cover in each of our molecular networks, see Supplementary Table S4.

#### 2.1.3 Gene-paralog annotation data

We determine for each species a set of *paralogs*, homologous genes that have diverged within one species due to gene duplication events (Koonin 2005). We derive them computationally using the procedure of Pearson (2013). That is, for each species, we collect all of its protein sequences from Ensemble (Yates *et al.* 2022) and compute their pairwise sequence alignments using BlastP (Altschul *et al.* 1997). We consider pairs of genes with a percentage of sequence identity ≥ 85%, an E-value ≤ 0.001 and a bit score ≥ 50 as paralogous. For details on the numbers of paralogs per network, see Supplementary Table S5.

### 2.2 Coalescent embedding

Coalescent embedding (CE) maps a network onto a disk so that nodes in the same neighbourhood (i.e. nodes that cluster) are assigned a similar angle and so that topologically important nodes (i.e. high-degree nodes), are embedded closer to the disk’s centre (Muscoloni *et al.* 2017). Formally, the CE algorithm consists of three steps:

1. The given network is embedded into a 2D space, such that nodes that are connected in the network have similar (Cartesian) coordinates. Suggested algorithms include Laplacian eigenmaps (LE) (Belkin and Niyogi 2003) and Isomap (Tenenbaum *et al.* 2000). This 2D Cartesian embedding is the basis of a 2D polar embedding in which nodes are assigned an angular and radial coordinate in step 2 and 3.
2. The nodes’ Cartesian coordinates are mapped to angular coordinates as follows. First, considering the origin of the 2D embedding as the centre of a disk and the *y*-axis as the pole (i.e. as a point reference on the disk), the angle between each node and the pole is computed. Then, all nodes are ranked based on this angle and placed equidistantly on the periphery of a disk.
3. A radial coordinate is assigned to each node, i.e. a distance from the centre of the disk, that reflects the node’s topological importance. CE explicitly assumes that the degree distribution of the network follows a power law: $P(d)∼d^{\lambda }$. So first, CE fits a power-law to the degree distribution (i.e. estimates λ). Then, the nodes are sorted in descending order according to their degree. Finally, for each node u, its radial coordinate, $rad_{u}$, is calculated as:
  (2) $$rad_{u}=\beta \ln(r_{u})+(1-\beta )\ln(N),$$

where $r_{u}$ is the rank of u, N is the number of nodes in the network and β=1/(λ−1).

### 2.3 Graphlet coalescent embedding (GraCoal embedding)

We generalize CE to graphlet-based Coalescent (GraCoal) embedding. We provide a visual summary of GraCoal embedding in Supplementary Fig. S2. Informally, for a given graphlet, GraCoal embedding maps a network onto a disk so that nodes that tend to be frequently connected by that graphlet are assigned a similar angle, and so that nodes with high counts of this graphlet are nearer to its the centre. Formally, our GraCoal embedding follows three steps analogous to those of CE:

1. For a given network and graphlet $G_{i}$, we embed the network into a 2D space using Graphlet Spectral embedding.
2. We map the nodes’ Cartesian coordinates to angular coordinates.
3. We compute a radial coordinate for each node by applying the following formula:
  (3) $$rad_{u}=\ln(r_{u}),$$

where $r_{u}$ is the rank of u based on the number of times it touches graphlet $G_{i}$. The graphlet count distributions for our real networks do not all follow a power-law. To account for this, our formula to determine the radius of a node [Equation (3)] is a simplified version of the equation applied in standard Coalescent embedding [Equation (2)]. We discuss this in Supplementary Section S3.1.

### 2.4 SAFE meets graphlet-based embeddings

Given a biological network and a set of node annotations of interest, SAFE uncovers annotations that are statistically overrepresented in regions of the network and provides an intuitive visual representation of their relative positioning within the network (Baryshnikova 2018). It does so by embedding the network in a 2D space and uncovering regions in the 2D space where nodes with a given annotation co-occur more often than expected by change (using a hypergeometric test). Whereas the original SAFE software only considers Spring embedding, we include our different graphlet-based embeddings: GraCoal (see Section 2.3), GraSpring and Graphlet Spectral embeddings (see Supplementary Sections S2.2 and S2.3).

The SAFE pipeline consists of four steps:

1. The network is embedded in 2D space. We consider GraCoal, GraSpring, and Graphlet Spectral embeddings.
2. The local neighbourhood of each node is determined based on information from the embedding space and information directly from the network. First, SAFE weights each edge of the original network by the Euclidean distance between the two corresponding nodes’ embedding. Then, SAFE computes the weighted shortest path distance (WSPD) between all nodes in the network. Finally, SAFE considers a node’s local neighbourhood to be the node itself and all nodes at a WSPD less than a chosen threshold α. We tune α for each type of embedding algorithm so that the average neighbourhood size is 50 using an elbow method (see Supplementary Section S3.2).
3. SAFE computes for each local neighbourhood the annotations that occur more than expected by chance using a hyper-geometric test (considering only the annotated nodes, applying Benjamini and Hochberg correction for multiple hypothesis testing per local neighbourhood). SAFE considers an annotation to be enriched if it is statistically significantly overrepresented in the local neighbourhood of at least one gene. SAFE considers a gene to be enriched if at least one annotation is enriched in its local neighbourhood.
4. The annotations that are enriched in overlapping sets of local neighbourhoods are aggregated into more descriptive groups, called *domains*, using hierarchical clustering. First, the annotations that are enriched in fewer than β local neighbourhoods are discarded (default: β = 10). Then, for all pairwise combinations of the remaining annotations, their overlap in terms of local neighbourhoods in which they are both enriched is measured using the Jaccard Index (JI). The JI ranges between 1 and 0, 1 indicating the two annotations are enriched in exactly the same set of neighbourhoods and 0 indicating the two annotations are never enriched in the same neighbourhood. Next, agglomerative hierarchical clustering is applied on the remaining annotations. Clusters of annotations are extracted by cutting the tree at γ% of its height (default: γ = 75%). These clusters of annotations are referred to as functional domains. Each domain is described by the five most repeated words occurring in the annotations names, ignoring uninformative words.

## 3 Results

First, we investigate the functional organization captured by GraCoal embeddings using the SAFE framework. We benchmark our method against GraSpring embedding (as GraSpring for graphlet $G_{0}$ corresponds to standard Spring embedding, SAFE’s default embedding method) and Graphlet Spectral embedding (as it underlies our GraCoal embeddings). As we find some GraCoal embeddings are more enriched than others, we subsequently perform a detailed investigation of the topology-function relationships that they capture.

In the main paper, we focus on the results for our four GI networks (fruit fly, budding yeast, fission yeast and *E. coli*). For the specific examples of the topology-function relationships, we focus on the budding yeast GI network, as it is the most complete and best annotated. We find that GraCoal embeddings best capture the functional organization of this network in terms of GO-BP annotations, which we present here. The results for the other annotations (i.e. GO-CC and GO-MF) and the other networks (i.e. GIS and PPI) are in Supplementary Section S3.4.

### 3.1 GraCoal best captures the organization of GI networks

**Figure 2.**
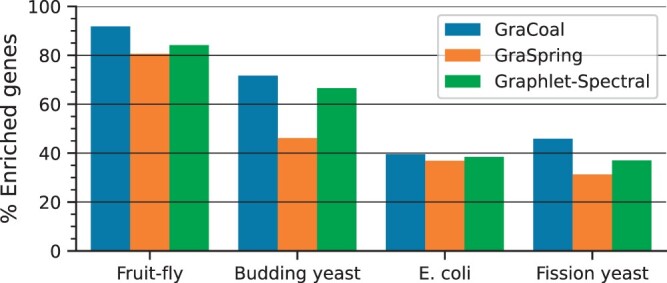
SAFE GO-BP enrichment analysis for GI networks. For the GI networks of our four species (*x*-axis), we show the percentage of enriched genes (*y*-axis) for each of the embedding algorithms considered (color coded).

We evaluate how well GraCoal embeddings capture the functional organization of GI networks via SAFE-enabled enrichment analysis: the more annotations or genes that are enriched, the better an embedding captures the functional organization of the network. As our conclusions are the same based on gene enrichment or annotation enrichment, we focus on gene enrichment here. As various GraCoal embeddings capture different aspects of the functional organization of the network, to measure the total amount of biological knowledge captured by GraCoal embeddings, we consider the union of the enriched genes across the different underlying graphlet adjacencies (i.e. ${\tilde{A}}_{G_{0}}$ to ${\tilde{A}}_{G_{8}}$). To compare against a fair baseline, we compare GraCoal to the graphlet-based extension of Spring embedding and Spectral embedding: GraSpring and Graphlet Spectral embedding. To render GraSpring deterministic, we use Graphlet Spectral embedding as its initialization. We show the results in Fig. 2.

**Figure 3.**
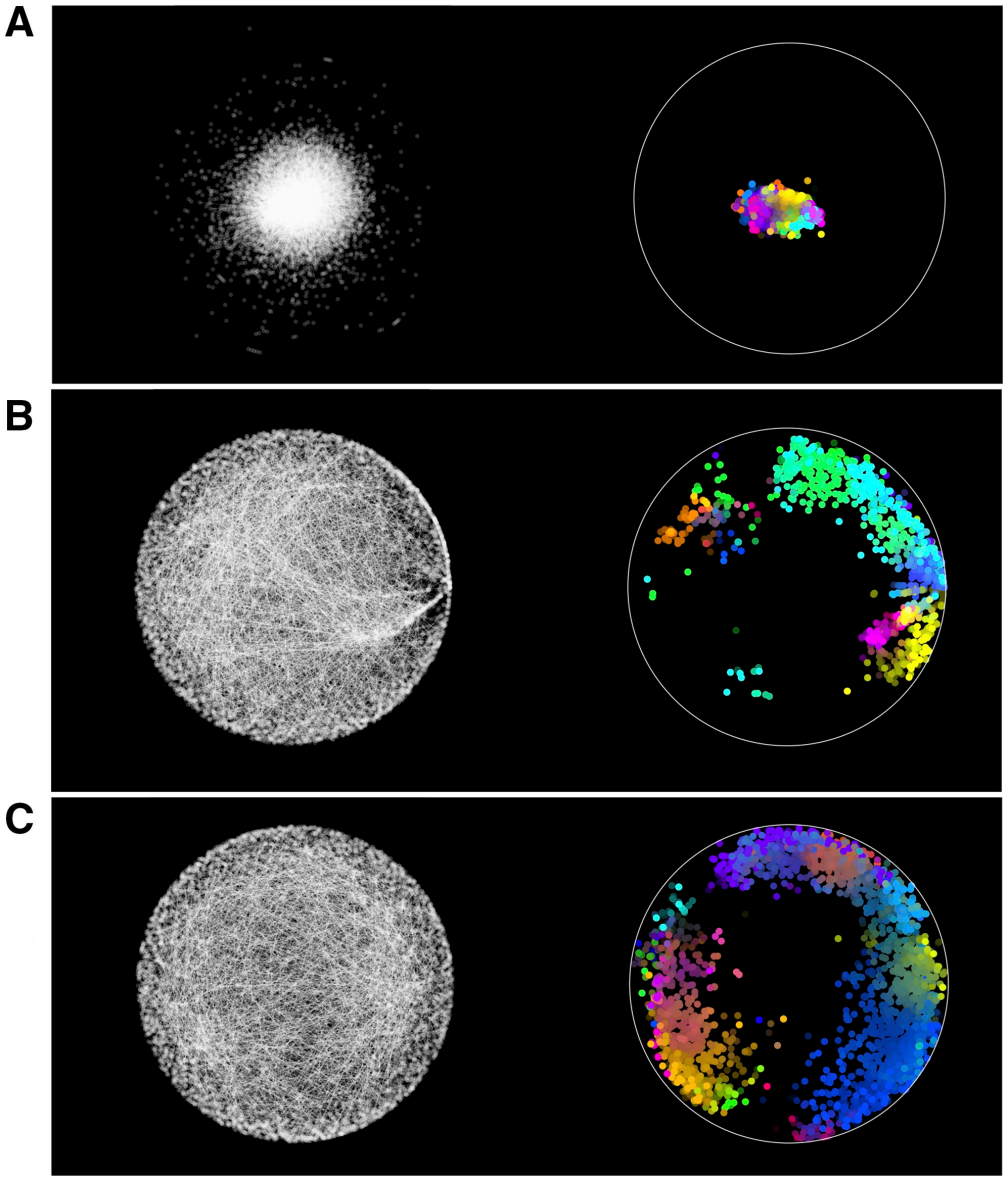
Functional maps of the budding yeast GI network. On the left hand side of sub-plots A, B, and C, we show the Spring Embedding, GraCoal_0_ embedding and GraCoal_2_ embedding of the budding yeast GI network. On the right hand side, we show the functional maps produced by SAFE using GO-BP annotations, which overlay these embeddings with coloured functional domains (details provided in Supplementary Section S3.3).

We observe that GraCoal best captures the functional organization of GI networks, achieving on average 62.22% of enriched genes over all species, against 56.55% and 48.71% of enriched genes for Graphlet Spectral and GraSpring, respectively. In particular, we find that GraCoal captures the functional organization of the fruit fly and budding yeast exceptionally well (91.78% and 71.67% of enriched genes, respectively), greatly outperforming Graphlet Spectral embedding (84.16% and 66.59% of enriched genes, respectively) and GraSpring embedding (80.59% and 46.16% of enriched genes, respectively). To explain this result, we show that GraCoal embeddings spread the nodes much more evenly in the embedding space than GraSpring embedding and Graphlet Spectral embedding: the average normalized distance between the nodes in GraCoal embeddings is roughly two and ten times that of the corresponding GraSpring and Graphlet Spectral embeddings (see Supplementary Table S6). In Fig. 3, we illustrate this result for Spring embedding (the SAFE default), GraCoal_0_ embedding (based on standard adjacency, like the default Spring embedding) and GraCoal_2_ embedding (the best performing GraCoal in budding yeast, see Section 3.2). Clearly, the Spring embedding is not able to separate the nodes in space as well as GraCoal_0_ and GraCoal_2_.

GraCoal also outperforms Graphlet Spectral and GraSpring embedding in terms of the union of enriched genes when we consider GO-CC annotations, which describe the localization of proteins in the cell, or GO-MF annotations, which describe the function of individual proteins (see Supplementary Figs S19 and S24, respectively). Hence, GraCoal embeddings capture the functional organization in GI networks best, regardless of the type of function considered. We show that this conclusion is robust to noise in the annotation data, to noise in the network, and that it is also insensitive to the tuning of SAFE’s neighbourhood parameter, see Supplementary Sections S3.9, S3.10, and S3.11, respectively.

For the six PPI networks, the results are mixed (see Supplementary Figs S17, S22, and S27). In terms of the union of enriched genes, GraCoal outperforms Graphlet Spectral and GraSpring embedding for three, two and one of the species when considering GO-BP, GO-CC, and GO-MF annotations, respectively. For the GIS network, GraCoal only outperforms Graphlet Spectral and GraSpring in terms of the union of enriched annotations of GO-BP terms and GO-CC terms (see Supplementary Figs S15, S20, and S25).

### 3.2 GraCoal embeddings capture different functions

**Figure 4.**
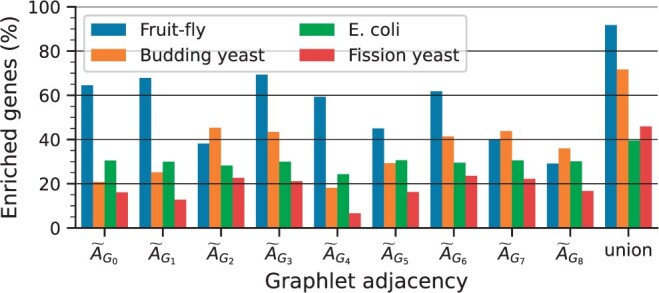
SAFE GO-BP enrichment analysis comparing GraCoal embeddings in GI networks. For the GI networks of the four species (color coded), we show the percentage of enriched genes (*y*-axis) for each of the different GraCoal embeddings (*x*-axis).

Next, we investigate which GraCoal captures the most function in GI networks. We present our results in Fig. 4. We observe that for the two species where GraCoal embeddings capture the most function, i.e. fruit fly and budding yeast, there are clear top performing GraCoal embeddings. For budding yeast for instance, the top performing GraCoal embeddings (GraCoal_2_, GraCoal_3_, GraCoal_6_, and GraCoal_7_) achieve between 41.4% and 45.2% of enriched genes, which is distinctly better then the low performing GraCoal embeddings (GraCoal_0_, GraCoal_1_, GraCoal_4_, GraCoal_5_, and GraCoal_8_), which achieve between 18.1% and 36.0% of enriched genes. Interestingly, we observe that the top performing GraCoal embeddings are not the same across the species, as those for fruit fly (GraCoal_0_, GraCoal_1_, GraCoal_3_, GraCoal_4_, and GraCoal_6_) are clearly distinct from those for budding yeast (GraCoal_2_, GraCoal_3_, GraCoal_6_, and GraCoal_7_). Notably, GraCoal_2_ and GraCoal_7_, both based on triangles, perform particularly well in budding yeast, achieving 45.3% and 43.8% of enriched genes. In contrast, these GraCoal embeddings perform poorly in fruit fly, achieving 38.2% and 40.0% of enriched genes. Conversely, GraCoal_0_, GraCoal_1_, GraCoal_3_, and GraCoal_4_, all based on graphlets void of triangles, perform particularly well in fruit fly, achieving 64.47%, 67.9%, 69.3%, and 59.3% of enriched genes. These same GraCoal embeddings (with the exception of GraCoal_3_) perform poorly in budding yeast, achieving 20.9%, 25.1%, 43.1%, and 18.1% of enriched genes. For fission yeast, the best performing GraCoal embeddings (GraCoal_2_, GraCoal_3_, and GraCoal_6_) largely follow those of budding yeast, although the differences in performance between the different GraCoal embeddings are less pronounced. For *E. coli*, there are no clear best GraCoal embeddings. We conclude that triangle-based GraCoal embeddings (GraCoal_2_ and GraCoal_7_) best capture the functional organization of the budding yeast, fission yeast and *E. coli* GI networks, and that GraCoal embeddings void of triangles (GraCoal_0_, GraCoal_1_, GraCoal_3_, and GraCoal_4_) tend to best capture the functional organization of the fruit-fly GI network. We show that this conclusion is robust to noise in the annotation data, to noise in the network, and that it is also insensitive to the tuning of SAFE’s neighbourhood parameter, see Supplementary Sections S3.9, S3.10, and S3.11, respectively.

As expected, we find that the union of the enriched genes over all GraCoal embeddings outperforms those of the individual GraCoal embeddings. For instance, in budding yeast we observe that the union of the enriched genes covers 71.4% of all genes. This is consistent with the literature, as different Graphlet spectral embeddings are known to capture complementary topology-function relationships in molecular networks (Windels *et al.* 2019). To describe the topology-function relationships captured uniquely by each GraCoal in each species, we identify the GO-BP functions only enriched for that particular GraCoal (see Supplementary Tables S10–S13). In budding yeast, we observe that on average 22 GO-BP functions are uniquely enriched for each particular GraCoal. For instance, GraCoal_2_ is the only GraCoal embedding to capture the GO-BPs ‘double-strand break repair via nonhomologous end joining’ and ‘positive regulation of DNA metabolic process’. To better summarize the biology captured by each GraCoal for each species, we use SAFE’s ability to combine the enriched GO-terms into functional domains (see Section 3.4).

In summary, we observed that GraCoal embeddings capture different topology-function relationships in a given GI network. The GraCoal embedding that captures the most function depends on the species. In particular, either triangle based graphlets or graphlets void of triangles tend to perform well.

### 3.3 GraCoal_2_ captures functional redundancy

We observed that triangle-based GraCoal embeddings (GraCoal_2_ and GraCoal_7_ embeddings) or GraCoal embeddings void of triangles (GraCoal_0_, GraCoal_1_, GraCoal_3_, and GraCoal_4_) tend to best capture the functional organization of GI networks, depending on the species. As GraCoal_2_ works the best for budding yeast, and achieves close to the best performance in *E. coli* and fission yeast, we focus here on characterizing the topology-function relationships captured by GraCoal_2_ in GI networks, to explain its performance and understand the biology it captures. We start by characterizing the topology of the GI networks and then relate our findings to our enrichment results. To characterize the topology of the GI networks, we identify for each GI network the most similarly wired type of *model network*, a randomly generated network with known graph-theoretic properties, and assume that our GI networks share their organizational properties. To perform this model fitting experiment, we apply the procedure outlined in (Malod-Dognin *et al.* 2019), as described next.

For each GI network, we generate fifteen instances of eight well-studied networks, matching their hyper-parameters to the properties of our GI networks (e.g. matching the numbers of nodes and edges, etc. See Supplementary Section S2.5.2). To measure the wiring similarity of the GI networks and the generated networks, we use the Graphlet Correlation Distance (GCD) (see Supplementary Section S2.5.1), a powerful network distance measure widely used in network biology (Ömer Nebil Yaveroğlu *et al.* 2014, Ruiz *et al.* 2017, Ullmann *et al.* 2023). A model network fits a GI network if its wiring cannot be distinguished from that of the real network. We determine this by applying a Mann-Whitney U (MWU) test on the distribution of GCD distances between our real network (GI) and the generated instances of the given model network type and the distribution of distances between the generated model networks themselves. A model network fits a real network when the MWU *P*-value is nonsignificant (i.e. >0.05). We present the results in Supplementary Table S7.

We observe that the topologies of the GI networks for budding yeast, *E. coli* is fitted by one model network: the Scale-Free Gene Duplication (SF-GD) networks (*P*-values >0.05). In contrast, the GI network of fruit fly is not fitted by any model network (*P*-value <0.05), although SF-GD is still the best-fitting model. This result is in line with the literature, as GI networks are known to be scale-free (Tong *et al.* 2004). As the SF-GD network generation procedure is designed to representing the evolution of molecular interaction networks in the presence of gene duplication events, it suggests that numerous gene-duplications may have influenced the topologies of the GI networks of budding yeast, *E. coli* and fission yeast. Indeed, we find that these species have up to fifteen times more paralogous genes in their GI network than fruit fly (31%, 32%, and 15% of the genes are paralogous in budding yeast, *E. coli* and fission yeast, respectively, compared to 2% in fruit fly, see Supplementary Table S5). This is consistent with the literature, as the genome of budding yeast has undergone a whole genome duplication event (Kuzmin *et al.* 2020), the genome of fission yeast has undergone similar duplications as budding yeast through individual gene duplications (Dujon 2010) and the genome of *E. coli* comprises many highly sequence similar gene families (Copley 2020).

We observe that our model fitting results align with our enrichment results: the GI networks that fit the SF-GD model (budding yeast, fission yeast and *E. coli*) are the networks for which GraCoal_2_ achieves the best enrichments. A possible explanation for this would be that paralogous genes are more likely to co-occur on triangles (i.e. share neighbours, forming graphlet $G_{2}$) leading to the high enrichments of GO-BPs involving paralogs. To test this hypothesis, we measure the number of triangles touched by the duplicated genes and the remaining genes in the GI networks. We find that for budding yeast, *E. coli* and fission yeast, the genes pairs of paralogous genes touch statistically significantly more triangles than pairs of nonparalogous genes (MWU *P*-values <0.05, see Supplementary Fig. S34). This is consistent with the literature, as one of the key drivers for paralog retention is functional redundancy, in which case paralogous genes are also likely to genetically interact and to share genetic interactions with the same genes, forming triangles in the GI network (Kuzmin *et al.* 2020). Looking at the number of duplicated genes enriched for each species and Gracoal, see Supplementary Table S9, we find that as expected for budding yeast, *E. coli* and fission yeast, GraCoal_2_ cover the most duplicated genes of all GraCoal embeddings (574, 321, and 186 duplicated genes, respectively, compared to 523, 286, and 91 on average for the other GraCoal embeddings). For fruit fly, whose genome is not characterized by the presence of large amounts of duplicated genes, this is not the case, with five GraCoal embeddings (GraCoal_0_, GraCoal_1_, GraCoal_3_, GraCoal_4_, and GraCoal_6_) having more duplicated genes enriched than GraCoal_2_. We conclude that in some species evolutionary forces have caused genes to duplicate, to obtain functional redundancy in biological processes, leading to the formation of triangles in the GI network. This topology-function relationship is captured by GraCoal_2_, leading to high enrichments of GO-BP involving paralogous genes.

### 3.4 Insight into the functions captured by GraCoal_2_

Here, we aim to give insight into the biological functions captured by GraCoal embeddings, and in particular by GraCoal_2_. For a given GraCoal embedding, we consider the domains it uncovers that overlap little with the domains uncovered by the other GraCoal embeddings as its *characteristic domains*. To identify the characteristic domains, we measure the pairwise overlap in terms of GO-BP between all the uncovered domains over all GraCoal embeddings using the Jaccard Index (JI). For each of the species, we list for each GraCoal embedding the top three domains that have the lowest maximum measured overlap (Max JI) as the GraCoal embedding’s most characteristic domains, see Supplementary Tables S14–S17. To relate these characteristic domains to our observations concerning paralogs, we report for each domain, the ratio of paralogous genes enriched in the domain over the total number of enriched genes in the domain—the domain’s *paralog ratio*.

Firstly, we observe that for fruit fly, budding yeast and fission yeast, GraCoal embeddings capture highly characteristic domains, with, respectively 7/92, 14/98, and 5/37 domains being completely unique (i.e. Max JI = 0.0). This is not the case for *E. coli*, for which only one of the 84 domains is completely unique. This is in line with our results at the GO-term level, where we observed fewer uniquely enriched GO-terms in *E. coli* (see Section 3.2). Secondly, we observe that for budding yeast and *E. coli*, the domains captured by GraCoal_2_ have on average the highest paralog ratios of all GraCoal embeddings: 0.2 and 0.22, respectively. In fission yeast GraCoal_2_ is just behind GraCoal_8_ in this respect, each scoring 0.14 and 0.15, respectively. In fruit fly on the other hand, the domains captured by GraCoal_2_ do not cover more paralogs than the other GraCoal embeddings. This is in line with our previous observation that GraCoal_2_ tends to capture GO-BP involving paralogs, in budding yeast, fission yeast and *E. coli*, but not in fruit fly. To show that the setting of the average neighbourhood size does not influence this conclusion, we show that the functional enrichments based on each GraCoal are highly stable when varying the average neighbourhood size from 50 up to 1000 in Supplementary Section S3.11.

We conclude by providing specific examples from the literature that illustrate the roles of the enriched paralogs in our domains. For instance, for budding yeast, the domain with the highest paralog ratio and that is completely unique is the domain described by key-words ‘membrane, cell, wall, chitin, process’, uncovered by GraCoal_8_ (JI = 0.0, paralog ratio 43%). This domain is composed of GO-BPs such as ‘cell wall chitin biosynthetic process’, ‘cell wall chitin metabolic process’, and ‘fungal-type cell wall chitin biosynthetic process’, which are all related to cell wall biosynthesis (i.e. the formation of the cell wall, which consists of chitin). A key element of this biosynthetic process is the ‘exomer’ protein complex, a heterotetrameric complex assembled at the trans-Golgi network, that is required for the delivery of a distinct set of proteins to the plasma membrane. Its cargo adaptors consist of two Chs5 proteins and two out of four paralogous proteins: Bud7, Bch1, Bch2, and Chs6. The paralogs part of the exomer complex determine which proteins it can transport (Anton *et al.* 2018). For instance, transport of Chs3 is completely dependent on the presence of Chs6 in the exomer. So, in the chitin biosynthetic process, gene duplication enabled different specializations of the exomer to transport different proteins, which is captured by GraCoal_8_. The domain uncovered by GraCoal_2_ in budding yeast that has the highest paralog-ratio, and that is also relatively unique, achieving the third lowest maximum JI score for GraCoal_2_, is the domain described by key-words ‘secretion, cell, exocytosis, export’ (JI = 0.12, paralog ratio 43%). This domain is composed of GO-BPs such as ‘export from cell’, ‘secretion by cell’, and ‘exocytosis’, which are all vesicle traffic related functions. It has been shown that, as paralogs can be differentially expressed or regulated, or can have different interaction partners, they contribute to the robustness and versatility of the vesicle traffic pathway (Purkanti and Thattai 2022). In conclusion, GraCoal_2_ captures functional redundancy and functional specialization in GI networks of species with many paralogs.

## 4 Conclusion

To better capture the functional organization of scale-free networks, whilst also considering different graphlet-based wiring patterns (e.g. triangles, paths…), we introduce the GraCoal embedding. We use our method to extend network analysis based on SAFE, a popular tool for biologists to investigate the functional organization of biological networks through the creation of 2D functional maps of the network. Through SAFE enabled enrichment analysis, we show that GraCoal embeddings better capture the functional organization of the fruit fly, budding yeast, fission yeast and *E. coli* GI networks, than Graphlet Spring embedding (which generalizes the Spring embedding, used in SAFE by default) and Graphlet Spectral embedding (which underlies GraCoal embedding). We show that depending on the graphlet considered, GraCoal embeddings capture different topology-function relationships. In particular, we show that triangle-based GraCoal embeddings capture the functional redundancy of paralogous genes.

Although we study molecular networks using GO annotations, our method is universal and can be applied on any type of network in any field. For instance, our method could be applied on a social network in combination with annotations indicating user interests, to create a map of the network highlighting groups of users with similar interests. In this work, we focus on extending the SAFE pipeline with our GraCoal embeddings to better capture the functional organization of scale-free networks. In future work, to uncover more functionally meaningful domains and to improve their descriptive power, we envision that the semantic similarity of the node annotations should be taken into account when computing the domains.

## Supplementary Material

btae650_Supplementary_Data

## References

[btae650-B1] Almagro P , Boguñá M, Serrano MÁ et al Detecting the ultra low dimensionality of real networks. Nat Commun 2022;13:6096. 36243754 10.1038/s41467-022-33685-z PMC9569339

[btae650-B2] Altschul SF , Madden TL, Schäffer AA et al Gapped BLAST and PSI-BLAST: a new generation of protein database search programs. Nucleic Acids Res 1997;25:3389–402. 9254694 10.1093/nar/25.17.3389 PMC146917

[btae650-B3] Anton C , Taubas JV, Roncero C et al The functional specialization of exomer as a cargo adaptor during the evolution of fungi. Genetics 2018;208:1483–98. 29437703 10.1534/genetics.118.300767 PMC5887143

[btae650-B4] Ashworth A , Lord CJ, Reis-Filho JS et al Genetic interactions in cancer progression and treatment. Cell 2011;145:30–8. 21458666 10.1016/j.cell.2011.03.020

[btae650-B5] Baryshnikova A. Spatial analysis of functional enrichment (SAFE) in large biological networks. Methods Mol Biol 2018;1819:249–68. 30421408 10.1007/978-1-4939-8618-7_12

[btae650-B6] Belkin M , Niyogi P. Laplacian eigenmaps for dimensionality reduction and data representation. Neural Comput 2003;15:1373–96.

[btae650-B7] Bläsius T , Friedrich T, Katzmann M et al Force-directed embedding of scale-free networks in the hyperbolic plane. In: *19th International Symposium on Experimental Algorithms (SEA 2021)*. Schloss Dagstuhl-Leibniz-Zentrum für Informatik. 2021.

[btae650-B8] Boguñá M , Bonamassa I, De Domenico M et al Network geometry. Nat Rev Phys 2021;3:114–35.

[btae650-B9] Cacciola A , Muscoloni A, Narula V et al Coalescent embedding in the hyperbolic space unsupervisedly discloses the hidden geometry of the brain. arXiv, arXiv:1705.04192, 2017, preprint: not peer reviewed.

[btae650-B10] Copley SD. Evolution of new enzymes by gene duplication and divergence. FEBS J 2020;287:1262–83. 32250558 10.1111/febs.15299 PMC9306413

[btae650-B11] Costanzo M , Baryshnikova A, Bellay J et al The genetic landscape of a cell. Science 2010;327:425–31. 20093466 10.1126/science.1180823 PMC5600254

[btae650-B12] Costanzo M , VanderSluis B, Koch EN et al A global genetic interaction network maps a wiring diagram of cellular function. Science 2016;353:aaf1420. 27708008 10.1126/science.aaf1420 PMC5661885

[btae650-B13] Dujon B. Yeast evolutionary genomics. Nat Rev Genet 2010;11:512–24. 20559329 10.1038/nrg2811

[btae650-B14] Fruchterman TM , Reingold EM. Graph drawing by force-directed placement. Softw Pract Exp 1991;21:1129–64.

[btae650-B15] Jeong H , Mason SP, Barabási AL et al Lethality and centrality in protein networks. Nature 2001;411:41–2. 11333967 10.1038/35075138

[btae650-B16] Koonin EV. Orthologs, paralogs, and evolutionary genomics. Annu Rev Genet 2005;39:309–38. 16285863 10.1146/annurev.genet.39.073003.114725

[btae650-B17] Kuzmin E , VanderSluis B, Nguyen Ba AN et al Exploring whole-genome duplicate gene retention with complex genetic interaction analysis. Science 2020;368:eaaz5667. 10.1126/science.aaz5667 PMC7539174 32586993

[btae650-B19] Li MM , Huang K, Zitnik M et al Graph representation learning in biomedicine and healthcare. Nat Biomed Eng 2022;6:1353–69. 36316368 10.1038/s41551-022-00942-x PMC10699434

[btae650-B20] Mair B , Moffat J, Boone C et al Genetic interaction networks in cancer cells. Curr Opin Genet Dev 2019;54:64–72. 30974317 10.1016/j.gde.2019.03.002 PMC6820710

[btae650-B21] Malod-Dognin N , Petschnigg J, Windels SFL et al Towards a data-integrated cell. Nat Commun 2019;10:805. 30778056 10.1038/s41467-019-08797-8 PMC6379402

[btae650-B23] Mast FD , Navare AT, van der Sloot AM et al Crippling life support for sars-cov-2 and other viruses through synthetic lethality. J Cell Biol 2020;219:e202006159. 10.1083/jcb.202006159 PMC7659715 32785687

[btae650-B24] Muscoloni A , Thomas JM, Ciucci S et al Machine learning meets complex networks via coalescent embedding in the hyperbolic space. Nat Commun 2017;8:1615. 29151574 10.1038/s41467-017-01825-5 PMC5694768

[btae650-B25] Oughtred R , Stark C, Breitkreutz B-J et al The BioGRID interaction database: 2019 update. Nucleic Acids Res 2019;47:D529–41. 30476227 10.1093/nar/gky1079 PMC6324058

[btae650-B26] Pearson WR. An introduction to sequence similarity (“homology”) searching. Curr Protoc Bioinform 2013;Chapter 3:3.1.1–.1.8. 10.1002/0471250953.bi0301s42 PMC3820096 23749753

[btae650-B27] Pržulj N , Corneil DG, Jurisica I et al Modeling interactome: scale-free or geometric? Bioinformatics 2004;20:3508–15. 15284103 10.1093/bioinformatics/bth436

[btae650-B28] Purkanti R , Thattai M. Genome doubling enabled the expansion of yeast vesicle traffic pathways. Sci Rep 2022;12:11213. 35780185 10.1038/s41598-022-15419-9 PMC9250509

[btae650-B29] Rauscher B , Heigwer F, Henkel L et al Toward an integrated map of genetic interactions in cancer cells. Mol Syst Biol 2018;14:e7656. 29467179 10.15252/msb.20177656 PMC5820685

[btae650-B30] Ravasz E , Barabási A-L. Hierarchical organization in complex networks. Phys Rev E 2003;67:026112. 10.1103/PhysRevE.67.026112 12636753

[btae650-B31] Ruiz VE , Battaglia T, Kurtz ZD et al A single early-in-life macrolide course has lasting effects on murine microbial network topology and immunity. Nat Commun 2017;8:518. 28894149 10.1038/s41467-017-00531-6 PMC5593929

[btae650-B32] Tenenbaum JB , de Silva V, Langford JC et al A global geometric framework for nonlinear dimensionality reduction. Science 2000;290:2319–23. 11125149 10.1126/science.290.5500.2319

[btae650-B33] The Gene Ontology Consortium. The gene ontology resource: enriching a GOld mine. Nucleic Acids Res 2021;49:D325–34. 33290552 10.1093/nar/gkaa1113 PMC7779012

[btae650-B34] Tong AHY , Lesage G, Bader GD et al Global mapping of the yeast genetic interaction network. Science 2004;303:808–13. 14764870 10.1126/science.1091317

[btae650-B35] Ullmann T , Peschel S, Finger P et al Over-optimism in unsupervised microbiome analysis: insights from network learning and clustering. PLoS Comput Biol 2023;19:e1010820. 36608142 10.1371/journal.pcbi.1010820 PMC9873197

[btae650-B36] Windels SFL , Malod-Dognin N, Pržulj N et al Graphlet laplacians for topology-function and topology-disease relationships. Bioinformatics 2019;35:5226–34. 31192358 10.1093/bioinformatics/btz455

[btae650-B38] Yates AD , Allen J, Amode RM et al Ensembl genomes 2022: an expanding genome resource for non-vertebrates. Nucleic Acids Res 2022;50:D996–1003. 34791415 10.1093/nar/gkab1007 PMC8728113

[btae650-B39] Yaveroğlu ÖN , Malod-Dognin N, Davis D et al Revealing the hidden language of complex networks. Sci Rep 2014;4:4547. 24686408 10.1038/srep04547 PMC3971399

