# Supplementary material for: Graphlet-based hyperbolic embeddings capture evolutionary dynamics in genetic networks
Source: Bioinformatics. 2024 Nov 4;40(11):btae650. doi: 10.1093/bioinformatics/btae650 (PMC11568109; doi:10.1093/bioinformatics/btae650)
Supplement: btae650_Supplementary_Data [file btae650_supplementary_data.pdf]

Supplementary materials:
"Graphlet-based hyperbolic embeddings capture evolutionary dynamics in genetic networks"

Sam F. L. Windels, Daniel Tello Velasco, Mikhail Rotkevich,
Noël Malod-Dognin, Nataša Pržulj

April 2022

Contents

1 Data **2**
1.1 Omics network data 2
1.2 Gene functional annotation data 3
1.3 Gene paralog data 3

2 Methods **4**
2.1 Graphlet adjacency illustrated 4
2.2 Graphlet Spring embedding 4
2.3 Graphlet Spectral embedding 5
2.4 GraCoal embedding 5
2.5 Model network fitting 5
2.5.1 Graphlet correlation distance 6
2.5.2 Model networks 6

3 Results **8**
3.1 Graphlet degree distributions 8
3.2 SAFE hyperparameter tunning: neighborhood size 12
3.3 Functional landscape of the budding yeast GI network 16
3.4 Enrichment statistics 20
3.4.1 Gene ontology biological processes enrichment statistics 21
3.4.2 Gene ontology cellular components enrichment statistics 25
3.4.3 Gene ontology molecular functions enrichment statistics 29
3.4.4 Comparing GO-BP enrichment statistics between different species 33
3.5 Model fitting 34
3.6 Gene paralog results 37
3.6.1 Genes enriched in *E. coli*, Fission yeast and Budding yeast GI networks contain more paralogs than those enriched in the Fruit fly GI network 37
3.6.2 Paralogous genes touch tend to form triangles in the network 37
3.7 GraCoal embeddings uncover unique biological functions in GI molecular networks 38
3.8 GraCoal embeddings uncover unique functional domains in GI networks 47
3.9 Robustness of key results to noise in the annotation data 51
3.10 Robustness of key results to noise in network data 55
3.11 Robustness of key results with respect to the average neighbourhood size 58

1 Data

1.1 Omics network data

We generate three types of molecular interaction networks: GI networks (nodes represent genes and edges represent genes genetically interacting), PPI networks (nodes represent genes and edges represent their protein products physically binding) and GIS networks (nodes represent genes and edges connect pairs of genes with highly similar genetic interaction profiles). We do this for human and the following model organisms: *Saccharomyces cerevisiae, Escherichia coli, Schizosaccharomyces pombe, Drosophila melanogaster, Mus musculus* and *Caenorhabditis elegans*, which throughout the text we refer to as Human, Budding yeast, *E. coli*, Fission yeast, Fruit fly, House mouse and Roundworm, respectively. For creating the GI and PPI networks, we collect molecular interaction data from the BioGRID database version 3.5.177 [1], filtering 'Genetic' or 'Physical' interactions, respectively. For the genetic interaction data, we filter by the following experimental evidence codes: 'Dosage Growth Defect', 'Dosage Lethality', 'Dosage Rescue', 'Negative Genetic', 'Phenotypic Enhancement', 'Phenotypic Supression', 'Positive Genetic', 'Synthetic Growth Defect', 'Synthetic Haploinsufficiency', 'Synthetic Lethality' and 'Synthetic Rescue'. Similarly, for the protein-protein interaction data, we filter by the following experimental high throughput capturing technologies: 'Affinity Capture-Luminescence', 'Affinity Capture-MS', 'Affinity Capture-RNA', 'Affinity Capture-Western' and 'Two-hybrid'. Next, to create GIS networks, there is only data available for the budding yeast, which we collect from [2]. This dataset contains the Pearson correlation coefficients (PCC) between the genetic interaction profiles of the genes. We construct a network as previously described by Costanzo et al. (2010, 2016) [3, 4], in which a gene (i.e., node) is linked to another (i.e., connected by an edge) if the PCC between the corresponding profiles is PCC$\geq$2.

Finally, we exclude the GI networks for Human, House mouse and Roundworm as they cover only very little of the known genes for these species (23.6%, 2.72% and 5.56%, respectively). Below we present the network statistics for the molecular interaction data used in our experiments.

		GI	
**Organism**	**Nodes**	**Edges**	**Density**
**Budding yeast**	5,842	447,747	0.03
E. coli	3,973	169,594	0.02
**Fission yeast**	3,577	52,402	0.008
**Fruit fly**	3,159	10,687	0.002

Supplementary Table 1: **GI molecular network data statistics.** For each species (rows), we report the number of nodes, the number of edges and the edge density of the corresponding GI network (columns 1-3).

		PPI	
**Organism**	**Nodes**	**Edges**	**Density**
**Budding yeast**	5,726	92,930	0.006
E. coli	2,022	12,788	0.006
**Fission yeast**	3,530	12,757	0.002
**Fruit fly**	8,864	54,722	0.001
**Human**	18,614	398,713	0.002
**House mouse**	10,164	55,640	0.001
**Roundworm**	7,628	32,502	0.001

Supplementary Table 2: **PPI molecular network data statistics.** For each species (rows), we report the number of nodes, the number of edges and the edge density of the corresponding PPI network (columns 1-3).

		GIS	
**Organism**	**Nodes**	**Edges**	**Density**
**Budding yeast**	4,626	30,185	0.003

Supplementary Table 3: **GIS Budding yeast molecular network data statistics.** We report the number of nodes, the number of edges and the edge density (columns 1-3).

1.2 Gene functional annotation data

	Organism	Annotations	Genes	PPI	GI	GIS
**GO-BP**	Budding yeast	4,621	5,105	4,576	4,502	3,457
	E. coli	2,773	2,564	1,223	1,935	na
	Fission yeast	2,624	739	604	601	na
	Fruit fly	6,317	5,777	3,529	2,418	na
	Human	11,368	9,659	9,228	na	na
	House mouse	12,353	9,933	5,726	na	na
	Roundworm	4,210	3,060	2,082	na	na
**GO-CC**	Budding yeast	960	4,652	4,045	3,973	3,058
	E. coli	221	2,139	1,204	1,708	na
	Fission yeast	574	767	641	605	na
	Fruit fly	911	3,762	2,801	1,886	na
	Human	1,539	10,648	10,205	na	na
	House mouse	1,223	7,979	4,973	na	na
	Roundworm	565	2,115	1,591	na	na
**GO-MF**	Budding yeast	2,143	4,124	3,678	3,603	2,663
	E. coli	2,128	2,592	1,311	1,968	na
	Fission yeast	879	695	588	560	na
	Fruit fly	1,872	3,227	2,372	1,699	na
	Human	3,705	14,270	13,715	na	na
	House mouse	2,782	8,287	5,398	na	na
	Roundworm	1,230	2,045	1,640	na	na

Supplementary Table 4: **Functional annotation data statistics.** For each of the four different annotation types (rows), we report the species, the total number of annotations, the total number of annotated genes and the number of annotated genes that occur in the PPI, GI and GIS networks (columns 1-6).

1.3 Gene paralog data

In this section we present the statistics of our sets of computationally derived paralogous genes, which are presented in Section *Gene-paralog annotation data* of the paper.

Organism	Paralogs	Total genes	Paralog content (%)
**Budding yeast**	1,870	6,000	31.17
E. coli	1,420	4,402	32.26
**Fission yeast**	798	5,122	15.58
**Fruit fly**	323	14,000	2.31

Supplementary Table 5: **Paralog data statistics for GI networks.** For the four GI networks (column 1), we report the number of paralogous genes in the network and total number of genes known to date for each species according to the UniProt database (columns 2 and 3). On column 4, we report the percentage of the total genes that are paralogous genes (Paralog content) and that are in the corresponding GI network.

2 Methods

2.1 Graphlet adjacency illustrated

In Section 1.2 of the main paper we recall the definition of graphlet adjacency [5], which aims to simultaneously capture neighbourhood and topology information. Here, we briefly recall the definition of the graphlet adjacency matrix and illustrate that definition on a toy example network. The graphlet adjacency matrix is defined is as:

$$A_{G_i}(u,v) = \begin{cases} c_{uv}^{G_i}/\theta_{G_i} & \text{if } u \neq v \\ 0 & \text{otherwise,} \end{cases} \tag{1}$$

where $c_{uv}^{G_i}$ is equal to the number of times the nodes u and v simultaneously touch graphlet G_i and θ_{G_i} is a scaling constant equal to the number of nodes in graphlet G_i minus 1. We illustrate the definition of graphlet adjacency applied on a toy network in Supplementary Figure 1. To compute our graphlet adjacency matrices, we use the GRaphlet-orbit ADjacency Counter (GRADCO) python package, available on GitHub at `https://github.com/samwindels/gradco` [6].

(A)

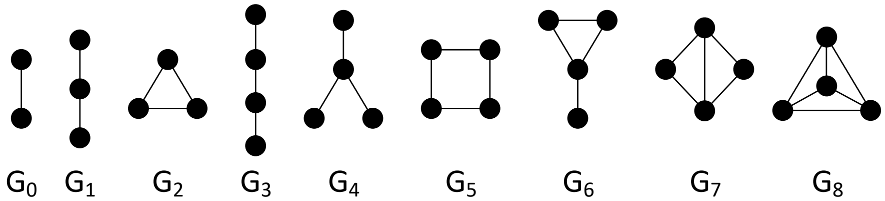

(B)

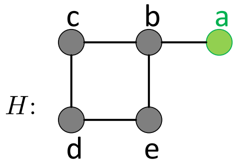

(C)

$$A_{G_0} = \begin{array}{c} \begin{matrix} a & b & c & d & e \end{matrix} \\ \begin{bmatrix} 0 & 1 & 0 & 0 & 0 \\ 1 & 0 & 1 & 0 & 1 \\ 0 & 1 & 0 & 1 & 0 \\ 0 & 0 & 1 & 0 & 1 \\ 0 & 1 & 0 & 1 & 0 \end{bmatrix} \end{array} \begin{matrix} a \\ b \\ c \\ d \\ e \end{matrix} \qquad A_{G_1} = \begin{array}{c} \begin{matrix} a & b & c & d & e \end{matrix} \\ \begin{bmatrix} 0 & 2 & 1 & 0 & 1 \\ 2 & 0 & 3 & 2 & 3 \\ 1 & 3 & 0 & 2 & 2 \\ 0 & 2 & 2 & 0 & 2 \\ 1 & 3 & 2 & 2 & 0 \end{bmatrix} \end{array} \begin{matrix} a \\ b \\ c \\ d \\ e \end{matrix}$$

Supplementary Figure 1: **An illustration of graphlets and graphlet adjacencies.** Node a is coloured green throughout. **A:** All graphlets with up to 4 nodes, labelled G_0 to G_8. **B:** Example network H. **C:** The graphlet adjacency matrices A_{G_0} and A_{G_1} for graphlets G_0 and G_1 of the example network H, shown in panel B. The off-diagonal elements correspond to the number of times two nodes touch a given graphlet together. $A_{G_0}(a,b) = 1$, as a and b form G_0 once. $A_{G_1}(a,b) = 2$, as a and b form G_1 twice, via paths a-b-c and a-b-e. This figure is adapted from Fig. 1 in [5].

2.2 Graphlet Spring embedding

Spring embedding refers to a family of network embedding methods that take inspiration from physical systems: all nodes in the network are assumed to repel each other with a repulsive force, $\vec{f_r}$, whilst connected nodes are assumed to be pulled together by springs applying an attractive force, $\vec{f_a}$. Nodes are placed in a 2D or 3D space so that the repelling and attracting forces exerted on them are in equilibrium.

Here, we focus on the Fruchterman–Reingold (FR) algorithm [7]. Formally, for two nodes u and v, the magnitudes of the attractive and repulsive forces are:

$$fa(d) = A(u,v)d^2/k, \tag{2}$$

$$fr(d) = -k^2/d, \tag{3}$$

where A is the adjacency matrix, d is the distance in the embedding space between node u and v and k is a hyper-parameter that balances the attractive and repulsing forces. By default, k is the square root of the area (volume) of the embedding space over the number of nodes.

To embed the network, the FR algorithm first places all nodes uniformly at random in the embedding space. Then, it computes for each node the sum of the repelling and attracting force vectors that push or pull the node in different directions in the 2D (3D) embedding space. This force vector is then applied on each node, displacing the nodes by an incremental step in the embedding space. This processes is repeated for a set number of iterations or until the applied forces are in equilibrium.

To generalise the Spring embedding to graphlet Spring embedding, we simply replace the adjacency matrix in equation 2 by one of the normalised graphlet adjacency matrices.

2.3 Graphlet Spectral embedding

In our Gaphlet Coalescent (Gracoal) embedding algorithm (see Section 2.4), the angular coordinates of the nodes are determined based their Graphlet Spectral embedding. Here, we recall our formal definition of Graphlet Spectral embedding [8].

A network representation related to the adjacency matrix is the *Laplacian*, which is defined as $\mathcal{L} = D - A$, where D is the diagonal matrix such that $D(u, u)$ is equal to the number of neighbours node u, i.e., its *degree*. The Laplacian matrix underlies so called *Spectral* network analysis methods, which use the property that the Laplacian's eigendecomposition naturally uncovers clusters in the network [**von˙Luxburg˙2007˙a˙tutorial**]. Spectral embedding refers to a family of methods that, based on the eigendecomposition of a Laplacian matrix representation, project a network into a lower dimensional space so that nodes that cluster in the network are embedded near each other in the space.

Given an unweighted network H with n nodes, we find a low dimensional embedding of the network, $Y = [\mathbf{y}_1, ..., \mathbf{y}_n] \in \mathbb{R}^{d\times n}$, such that if nodes u and v are frequently graphlet-adjacent with respect to graphlet G_k, then $\mathbf{y}_u$ and $\mathbf{y}_v$ are close in the d-dimensional space, by solving:

$$\begin{aligned} \underset{Y}{\text{minimize}} \quad & \sum_{u=1}^{n}\sum_{v=1}^{n} A_{G_k}(u,v)\|\mathbf{y}_u - \mathbf{y}_v\|^2 \\ \text{subject to:} \quad & YD_k\mathbf{1} = \mathbf{0} \text{ and } YD_{G_k}Y^T = I, \end{aligned} \tag{4}$$

where A_{G_k} is the graphlet-based adjacency matrix of H for graphlet G_k, D_{G_k} is the graphlet-based degree matrix of H for graphlet G_k. The two optimisation constraints aim to avoid all points being mapped to the origin, or to the same embedding $y \in \mathcal{R}^d$, respectively. The columns of Y are found as the generalized eigenvectors associated with the d smallest non-zero generalized eigenvalues solving $Y\mathcal{L}_{G_k} = \Lambda Y D_{G_k}$, where Λ is the diagonal matrix with the generalized eigenvalues along its diagonal and $\mathcal{L}_{G_i}$ is the graphlet Laplacian: $\mathcal{L}_{G_i} = D_{G_i} - A_{G_i}$.

2.4 GraCoal embedding

We define GraCoal embedding in Section 2.3 of the main paper. To ease understanding, here we present a visual summary of the Gracoal embedding algorithm in Suppl. Fig. 2.

2.5 Model network fitting

To enable our investigation of the topology-function relationships captured by Gracoal embeddings, we first describe the topology of our real networks relative to model networks, randomly generated networks with known graph-theoretic properties. The assumption underlying this approach, known as model network fitting, is that we assume that our real networks share the same organisational principles as the most similarly wired model networks. Below, we recall the definition of the graphlet correlation distance (GCD) [**yaveroglu˙revealing˙2014**], which we use to measure the topological similarity between our real and model networks. We also present the eight well studied model networks that we considered.

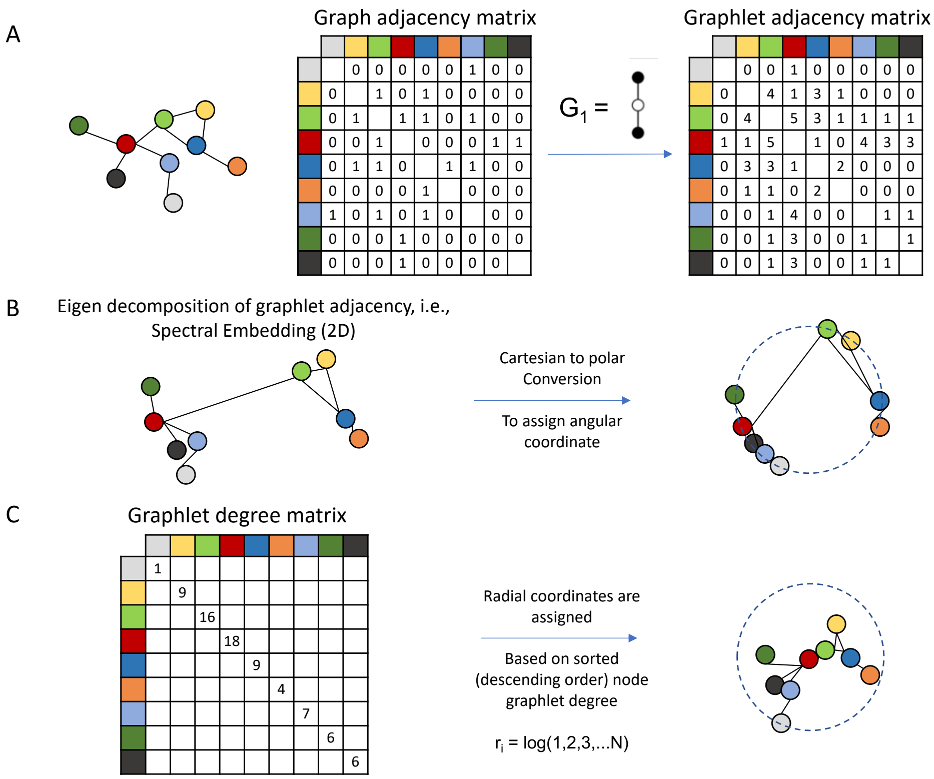

Supplementary Figure 2: **The GraCoal embedding algorithm**. A: Computing graphlet adjacency. For a given an input network and graphlet, the graphlet adjacency matrix is computed. Shown is the example for the three-node path topology ($\widetilde{A}_{G_1}$). B: Computing the angular coordinate. The network is embedded into a 2D Cartesian plane using Graphlet Spectral embedding. These Cartesian coordinates are subsequently converted to angular coordinates on a circle so that nodes nearby in the 2D Cartesian plane have a similar angle. C: Computing the radial coordinate. Nodes are assigned a radius based on the rank of their node degree. The procedure is the same for any other given graphlet adjacency.

2.5.1 Graphlet correlation distance

To measure the similarity in topology (i.e., wiring) between a real network and a type of model network, we first summarise the topology of the real network and randomly generated networks using the *Graphlet Correlation Matrix*, the state-of-the-art network descriptor [9]. The GCM is an 11 × 11 symmetric matrix of pairwise Spearman rank-correlations between the different non-redundant orbit counts over all nodes in a network, illustrated in Supplementary Figure 3. Then, we use the *Graphlet Correlation Distance* (GCD) to quantify their difference in wiring, which for two networks is defined as the Euclidean distance between the upper triangle part of their GCMs [9]. Then, we determine if a real network is topologically statistically significantly dissimilar from a given type of model network. We apply a Mann-Whitney-U (MWU) test, comparing the distribution of GCD distances between the real network and the generated model networks against the GCD distances between the generated model networks themselves. We assume that our real networks share the same organisational principles as the type of model networks that they can't be topologically distinguished from (i.e., non significant p-value, after Benjamini Hochberg correction).

2.5.2 Model networks

To characterize the structure of the molecular networks, we perform model-fitting experiments to compare our real molecular networks (e.g., each of our Budding yeast molecular networks) to eight different types of random model networks commonly used in biology. To do this, for a given real molecular network, we generate 15 random networks for each network model. To randomly generate synthetic networks that follow

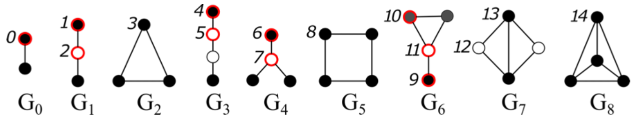

Supplementary Figure 3: **The nine 2- to 4-node graphlets and their 15 orbits**. Within each graphlet, nodes belonging to the same orbit have the same color (either white, black or grey). The orbits, also called automorphism orbits, are defined as symmetry groups of nodes within a graphlet, and are used to characterize different topological positions of a node in a graphlet. The ten non-redundant orbits, whose counts cannot be derived from the counts of the other orbits, are highlighted in red.

a particular model network, we set the number of nodes, edge density, node degree distribution, and the number of communities (when needed) to match those of the input data. To this end, we considered the following random model networks:

The Erdos Renyi (ER) random graph model represents uniformly distributed random interactions between a set of nodes. In this model, the probability of any two nodes being connected by an edge is the same for all pairs in the set [10]. Thus, networks that follow this model are characterized by a lack of local clustering, small diameter, and homogeneous node degree distribution, which also leads to a lack of hubs in the network. To generate the ER networks, we set the number of nodes and edge density to match those of the input real network, and by randomly adding edges between uniformly chosen pairs of nodes (out of the $n(n\text{-}1)/2$ possible pairs of nodes) until the desired/targeted density is reached in the network model.

The Generalized random graph model (ER-DD) is an extension of the ER model, where the distribution of the degrees of the nodes in the generated model network matches that of an input (data) network [11]. To generate ER-DD networks, we assign connection capacities (stubs, corresponding to the degree of a node) to the nodes of the network, and then add edges between nodes that have available stubs uniformly at random while reducing the available stubs of the newly connected nodes after each edge addition.

The geometric random graph model (GEO) represents the proximity relationship between uniformly distributed points in a d-dimensional space [12]. In this model, two nodes are connected by a link if the distance between them is below a certain range. Networks that follow this model are characterized by having high modularity (i.e, community structure) and by displaying assortativity (i.e., highly connected nodes are likely to be connected to other highly connected nodes). We generate GEO networks by distributing uniformly at random the set of nodes in three-dimensional space and connecting them by edges if the Euclidean distances between them are lower than or equal to threshold r. This value is set so that we obtain a given edge density. The number of nodes and edge density are set to match those of the real networks.

The GEO model with gene duplication (GEO-GD), where the dispersion of nodes is no longer uniformly random, but according to duplication and divergence rules, which mimics the gene duplication and mutation process in biology [13]. To generate a GEO-GD model network, we start from a seed network (i.e., two nodes connected by an edge) to which the duplication and mutation process is applied. First, a parent node is chosen at random and duplicated, and then the child node is randomly placed at a distance smaller than or equal to $2r$ (r is the same as in the GEO model). This process repeats itself until the required number of nodes matches that of the input data. The last step creates the edges with the same rules as in the GEO model until the edge density matches the input data.

The Barabàsi–Albert scale-free model (SF) is based on the preferential attachment principle, i.e., the rich get richer principle. Networks that follow this model are characterized by having the majority of their nodes very poorly connected (with one or very few edges) and a very small number of nodes that are highly connected. Thus, these networks follow a scale-free degree distribution which forms hubs in the network [14]. To generate an SF network, we start from a seed network (i.e., two nodes connected by an edge), and nodes are subsequently added and attached to existing nodes of the network with a probability proportional to their node degrees. This is repeated until the desired number of nodes is reached.

The scale-free model with gene duplication and divergence (SF-GD). Similar to the GEO-GD model, the SF-GD mimics the gene duplication and divergence processes in biology [15]. The initial process is the same as in the SF model, starting with a single edge, which is grown through iterative duplication and divergence events. In brief, for each iteration, a parent node is randomly selected and duplicated into a child node. The newly produced node is connected to all the neighbors of the parent node and it has a probability p

to be connected to the parent node as well. For the divergence process, connections between all the shared neighbors of the parent node and the newly duplicated node have a probability q to be removed. Parameter q is set to match the edge density of the input data.

The stickiness-index-based (STICKY) model, assumes that the higher the degree of two nodes, the higher is the probability that they interact [**przulj˙modelling˙2006**]. To generate a STICKY network, we start from n disconnected nodes, to which we randomly assign stickiness index values that are proportional to the node degrees of an input network. Then, the probability of connecting two nodes is equal to the product of their stickiness indexes. Networks that follow this model are characterized by displaying degree assortativity, where highly connected nodes are more likely to be connected to other highly connected nodes. To generate a STICKY network, we start with n disconnected nodes and we randomly assign stickiness index values which are proportional to the node degrees of the input data. The probability of connecting two nodes is equal to the product of their stickiness indexes. We stop connecting pairs of nodes after the desired number of edges is achieved.

Lastly, the nonuniform Popularity-Similarity Optimization (nPSO) model simulates how random geometric graphs grow in a hyperbolic space [16]. It is an extension of the Popular-Similarity Optimization (PSO) model, where the similarity between nodes is represented by the hyperbolic distance between them (i.e., the closer two nodes are in a hyperbolic space, the more likely they are to be connected by an edge). Similarly, the popularity of the nodes is represented by the radial coordinate in the hyperbolic plane, where nodes with a larger degree are positioned closer to the center of the circle. Networks that follow this model are characterized by displaying clustering, small-worldness (i.e., many nodes are not directly connected, but most nodes can be reached from every other by a small number of hops) scale-freeness, rich-clubness (i.e., well connected nodes tend to also connect to each other), and community organization [16]. To generate nPSO model networks, we set the number of nodes, half of the average node degree, the exponent of the power-law node degree distribution, and the number of communities to match those of the input data. For computing the number of communities we use the Clauset-Newman-Moore greedy modularity maximization algorithm [17].

3 Results

3.1 Graphlet degree distributions

The Coalescent embedding (CE) algorithm maps the nodes in a network onto a disk. Nodes of larger degree (i.e., of 'high importance') are embedded near the center of the disk and nodes that are similar (i.e., that cluster together in the network) are closer to each other by assigning them similar angles. CE explicitly assumes that the degree distribution of the network follows a power law: $P(d) \sim d^{\lambda}$. To determine the radius of the nodes, CE fits a power-law to the degree distribution (i.e., estimates λ). Then, the nodes are sorted in descending order according to their degree. The radial coordinate of each node is determined applying equation (4) in the main document: $rad_u = \beta ln(r_u) + (1-\beta) ln(N)$, where r_u is the rank of u, N is the number of nodes in the network and $\beta = 1/(\lambda - 1)$. The first term in equation (4) ($ln(r_u)$) makes it so that the lower the degree-based rank of a particular node, the more the node will be placed towards the periphery of the hyperbolic the space. Conversely, the higher the rank of a node, the more the node will be placed towards the center of the hyperbolic space. The second term of equation (4) is a constant ($ln(N)$. These two terms are balanced using a constant B, which is computed based on the power-law exponent λ. For real scale-free networks, the value of λ, usually ranges between 2 and 3 [18]. It is a measure used to quantify the degree heterogeneity of the network. The more extreme the degree heterogeneity (i.e., the larger the lambda), the less weight is assigned on the rank of the nodes by their degree, and the more all the nodes are placed towards the periphery at a constant distance. When the degree heterogeneity is well beyond that of a normal scale-free network, i.e., λ well exceeds 3, equation (4) ends up assigning very little value in the rank of the node degree, placing all nodes at a fixed distance from the centre of the hyperbolic space. This is illustrated in Suplementary Figure 7. Graphlet degree distributions do not all follow a scale-free distribution (Supplementary Figures 4-6). Often, especially for GI and GIS, fitting a power-law to the graphlet degree distributions leads to values of λ much greater than three, which makes equation (5) not directly applicable for CE embedding based on graphlets.

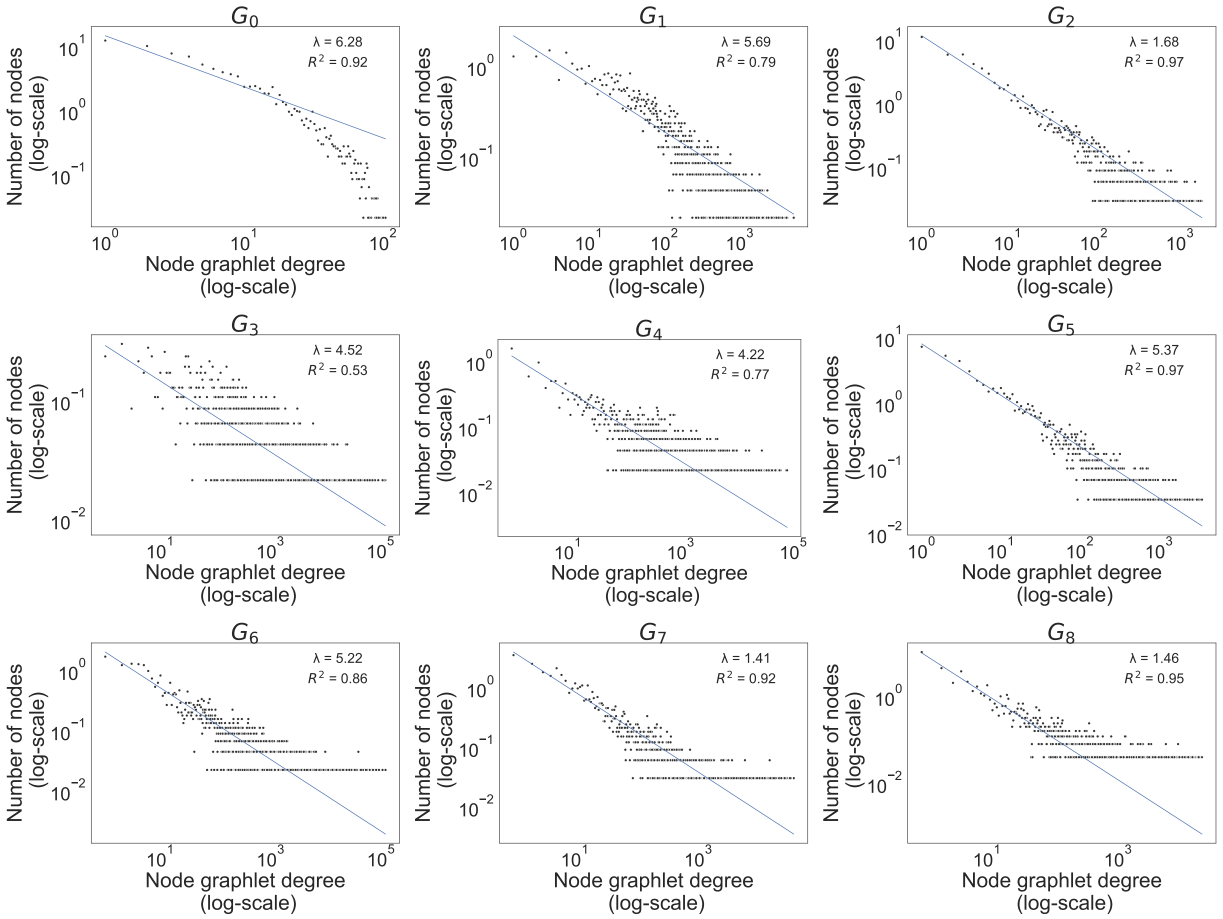

Supplementary Figure 4: **Node graphlet degree distributions for all up to 4-node graphlets (G_0-G_8) for the Budding yeast genetic interaction similarity (GIS) network.** For each of the 9 different 4-node graphlets, we show the node graphlet degree (x-axis) and the number of nodes (y-axis). The blue line shows the fitted power-law distribution. We also report the goodness-of-fit R-squared as well as the value of the power-law exponent λ (legend).

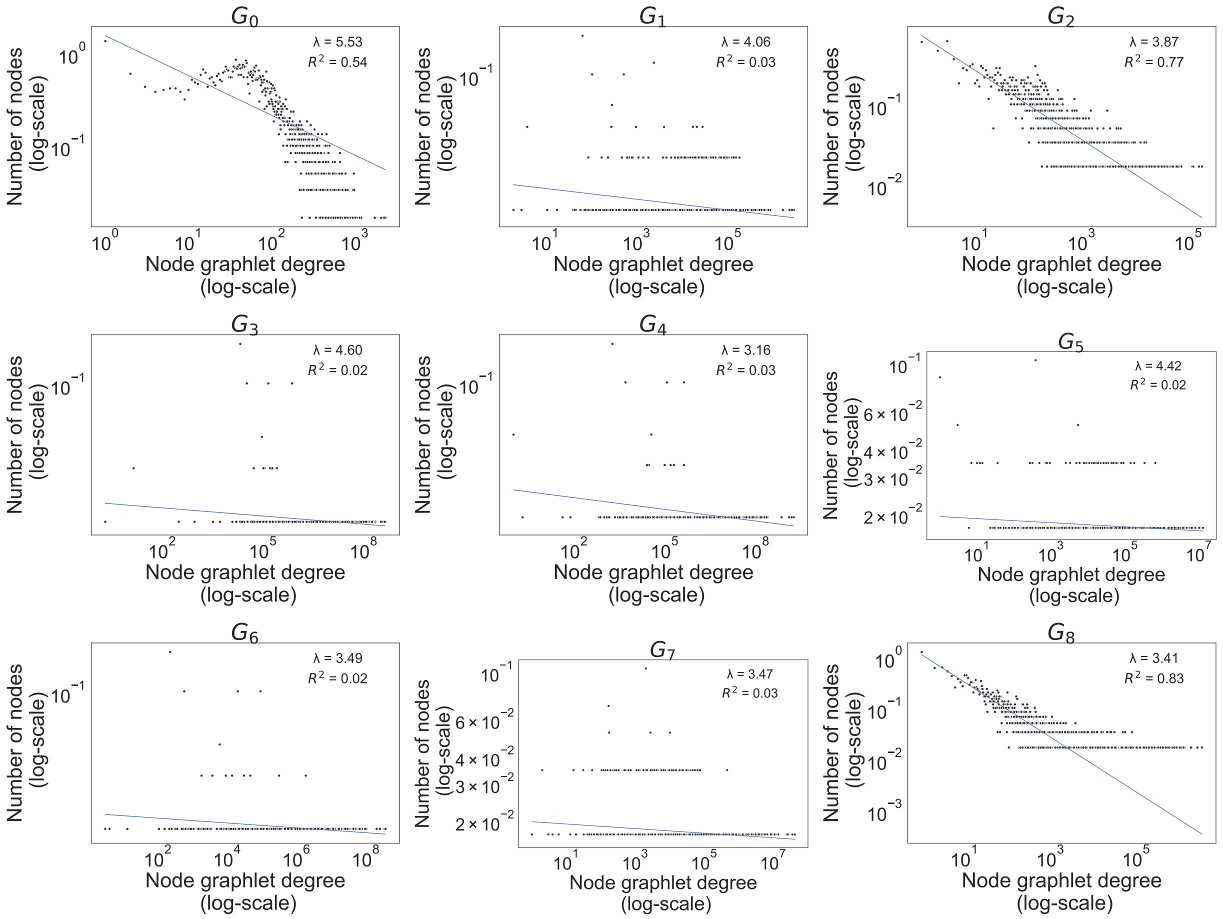

Supplementary Figure 5: **Node graphlet degree distributions for all up to 4-node graphlets (G_0-G_8) for the Budding yeast genetic interaction similarity (GI) network.** For each of the 9 different 4-node graphlets, we show the node graphlet degree (x-axis) and the number of nodes (y-axis). The blue line shows the fitted power-law distribution. We also report the goodness-of-fit R-squared as well as the value of the power-law exponent λ (legend).

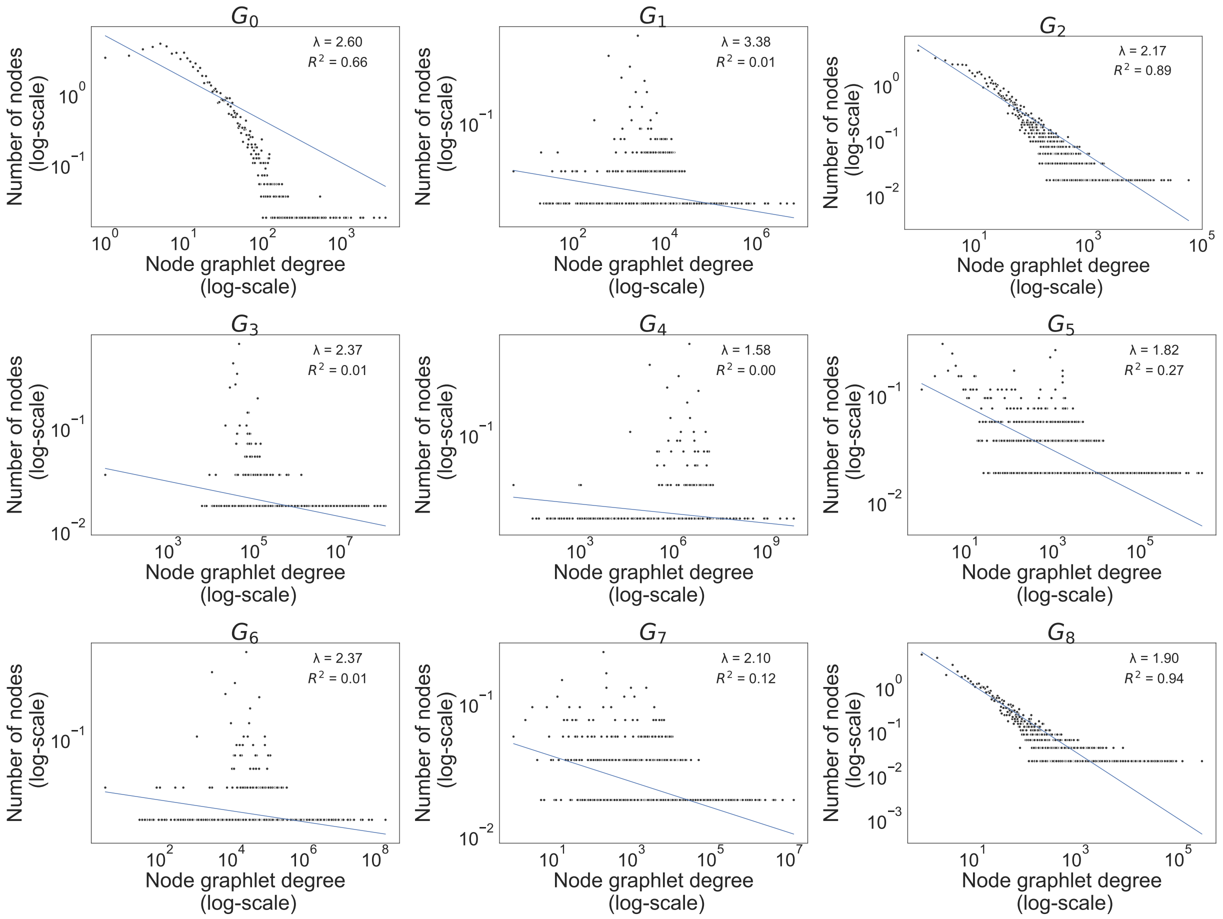

Supplementary Figure 6: **Node graphlet degree distributions for all up to 4-node graphlets (G_0-G_8) for the Budding yeast protein-protein interaction (PPI) network.** For each of the 9 different 4-node graphlets, we show the node graphlet degree (x-axis) and the number of nodes (y-axis). The blue line shows the fitted power-law distribution. We also report the goodness-of-fit R-squared as well as the value of the power-law exponent λ (legend).

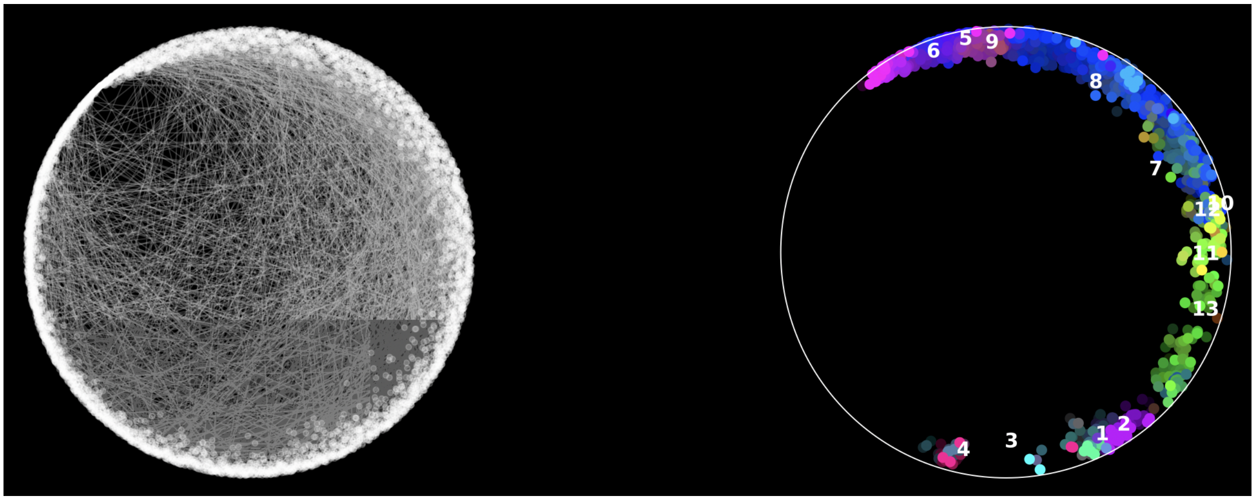

Supplementary Figure 7: **Original Coalescent embedding applied to graphlet adjacency $\widetilde{A}_{G_5}$.** When using the original Coalescent embedding based on Equation (5) on main document, large values of power-law exponent lead to large radial coordinates, placing the nodes towards the periphery of the embedding space. For instance, we show the Coalescent embedding (left hand side) and the corresponding enrichment landscape (right hand side), visualized with SAFE when annotating the Budding yeast GI network based on graphlet adjacency $\widetilde{A}_{G_5}$ (power-law exponent = 4.42). In this way, nodes with large graphlet degrees (i.e., that are very well connected, in this case with respect to G_5), are almost indistinguishable from nodes with smaller graphlet degrees, as all the nodes are embedded towards the periphery in the hyperbolic space when using Equation (4) to compute their radial coordinates.

3.2 SAFE hyperparameter tunning: neighborhood size

To choose an optimal neighborhood size, we run SAFE with increasing values of this hyperparameter (range 10 to 100 with steps of 20) with the three embedding algorithms and compare the percentages of genes that have at least one annotation enriched in their neighborhood and percentages of annotations enriched. In Supplementary Figures 8-**??** we show that the percentages of genes enriched become larger as we increase the size of the neighborhood. Furthermore, we observe that GraCoal embeddings tend to outperform graphlet-based Spring and graphlet-based Spectral embeddings over each tested neighborhood size. Lastly, the enrichments in terms of percentage of annotations enriched tend to plateau after neighborhood size is set to 50, even though the percentages of genes enriched in annotations continue to increase. Thus, we fix this hyperparameter and set it so that, on average, each node has a neighborhood of 50 nearby nodes for all of our comparison experiments.

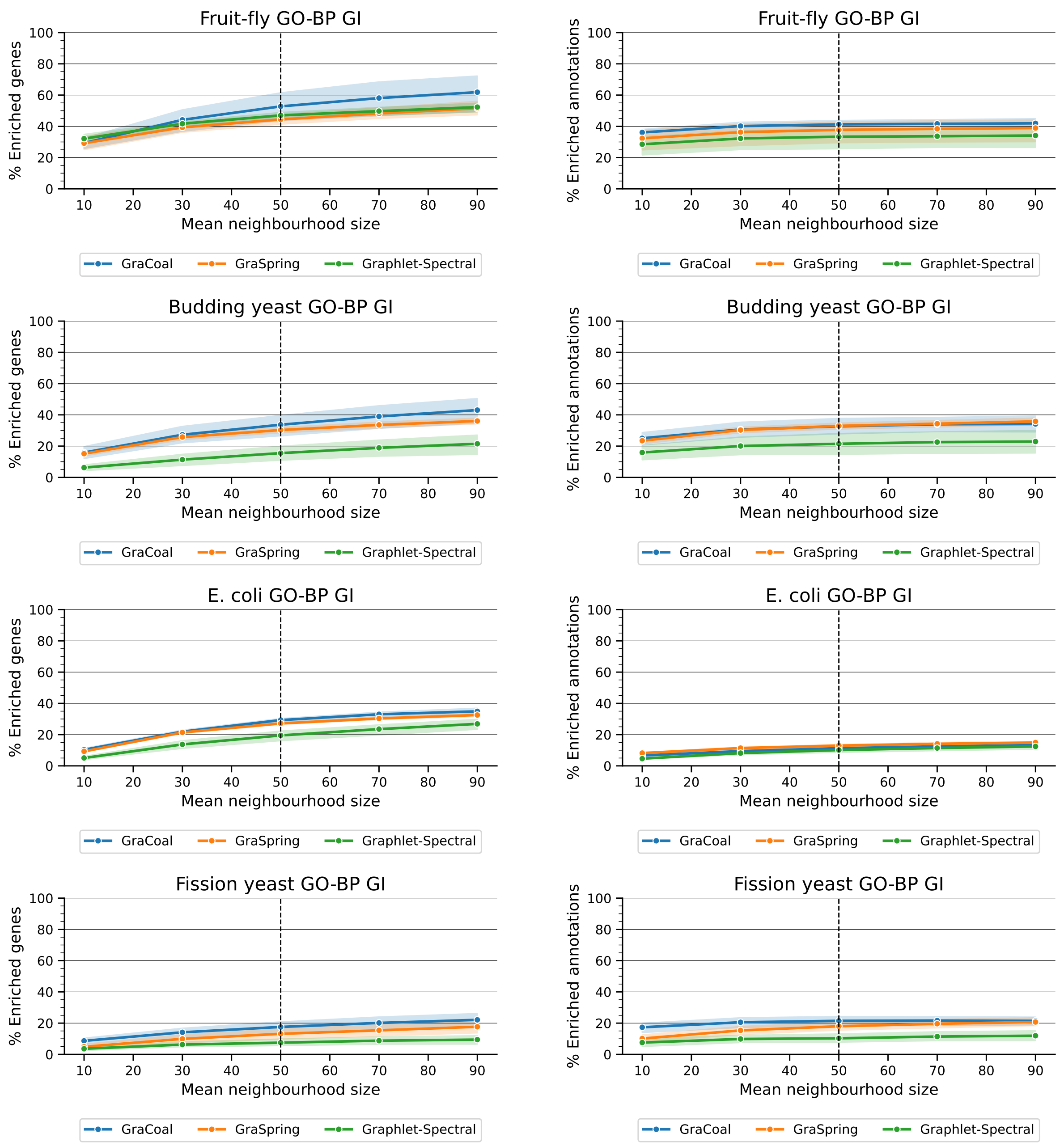

Supplementary Figure 8: **SAFE enrichment statistics with respect to neighborhood size** For the GI networks of the four species, we show the percentages of genes enriched in at least one GO-BP (left hand side) and percentages of enriched GO-BP (right hand side) for different neighborhood sizes used in SAFE (x-axis).

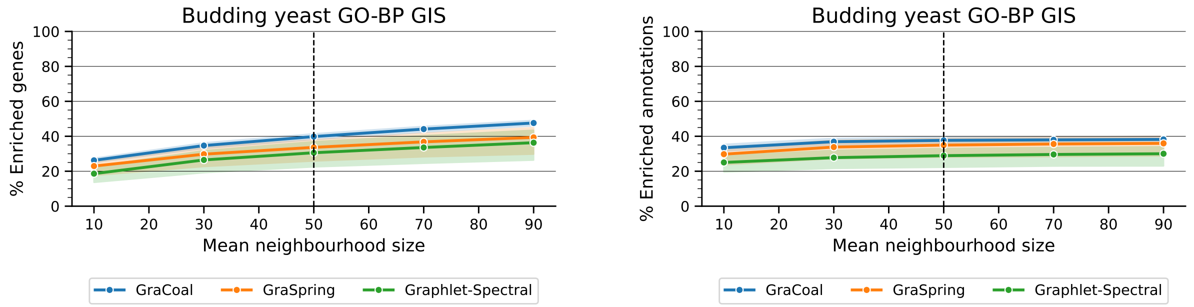

Supplementary Figure 9: **SAFE enrichment statistics with respect to neighborhood size.** For the Budding yeast GIS network, we show the percentages of genes enriched in at least one GO-BP (left hand side) and percentages of enriched GO-BP (right hand side) for different neighborhood sizes used in SAFE (x-axis).

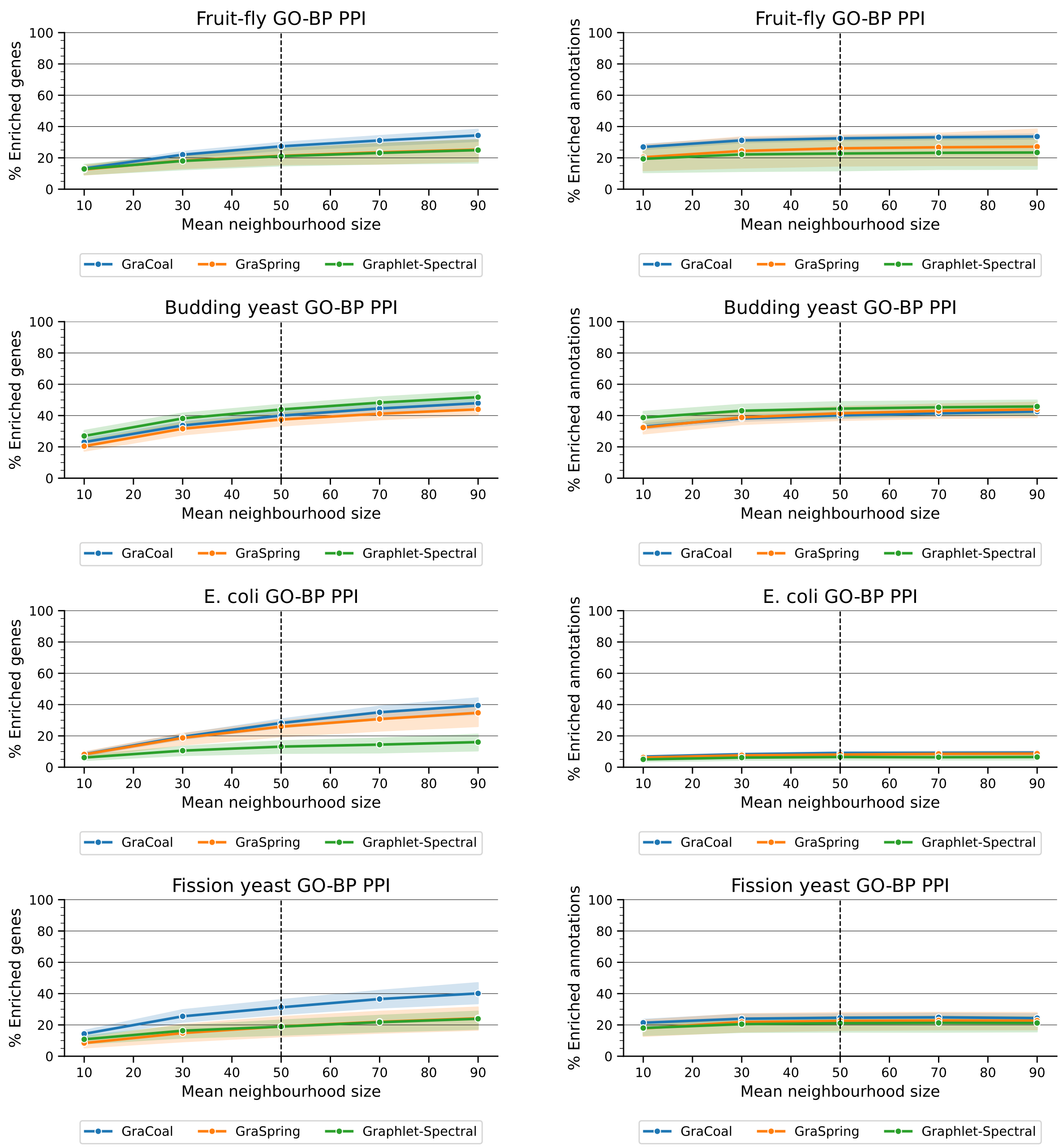

Supplementary Figure 10: **SAFE enrichment statistics with respect to neighborhood size, Part 1.** For the PPI networks of the six species, we show the percentages of genes enriched in at least one GO-BP (left hand side) and percentages of enriched GO-BP (right hand side) for different neighborhood sizes used in SAFE (x-axis).

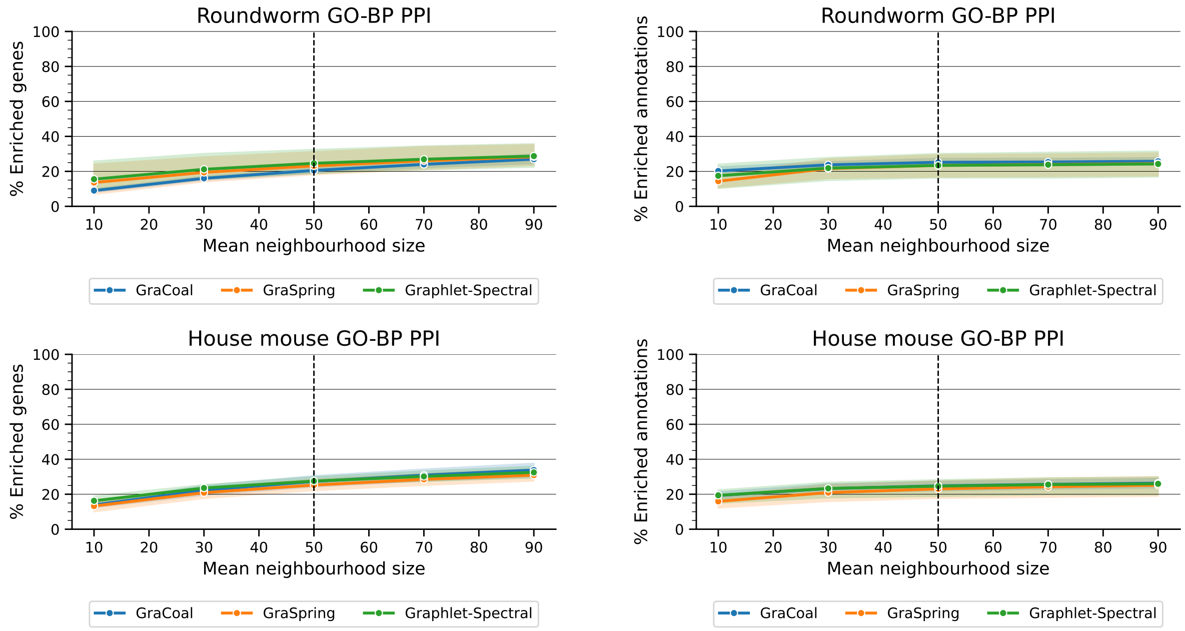

Supplementary Figure 10: **SAFE enrichment statistics with respect to neighborhood size, Part 2.** For the PPI networks of the six species, we show the percentages of genes enriched in at least one GO-BP (left hand side) and percentages of enriched GO-BP (right hand side) for different neighborhood sizes used in SAFE (x-axis).

3.3 Functional landscape of the budding yeast GI network

In general, we observe that GraCoal embeddings lead to the best enrichment results for our GI networks. A possible explanation for this is the fact that GraCoal embedding spreads the nodes better in the embedding space with respect to graphlet-based Spring embedding or graphlet-based Spectral embedding, which leads to better separated functional domains. Below we compare the 2-dimensional (2D) network embedding layouts (left hand side) and functional landscapes (right hand side) produced by SAFE of the three graphlet-based embeddings for the Budding yeast GI network. To have a baseline comparison, we first show the 2D plots corresponding to the normal graphlet adjacency in Supplementary Figure 11 (i.e., $\widetilde{A}_{G_0}$). Next, in Supplementary Figures 12 and 13, we show the 2D plots for two of the top performing GraCoals: based on graphlet adjacency $\widetilde{A}_{G_2}$ (i.e., the three-node clique), and graphlet adjacency $\widetilde{A}_{G_3}$ (i.e., the four-node path), respectively. For all other 2D plots of the network embedding layout and the functional landscape produced by SAFE of each of our GI and PPI molecular networks (i.e., for all species and all graphlet-based embeddings), please refer to Supplementary file functional-domains.zip. Next, we validate that this is consistent for the embedding layouts corresponding to the other graphlet adjacencies (i.e., not just based on $\widetilde{A}_{G_2}$ and $\widetilde{A}_{G_3}$). For each of the embedding methods, we measure the average Euclidean distance between all pairs of nodes. To allow for comparison between the three different embedding methods, we normalize these average embedding distances by dividing by the largest measured Euclidean distance for each of the corresponding embedding spaces and report these values in Supplementary Table 6. We observe that the average normalized distance between nodes when using GraCoal embedding, is approximately 2.11 to 3.56 times larger than when using GraSpring embedding and approximately 475 to 2,850 times larger than when using Graphlet Spectral embedding.

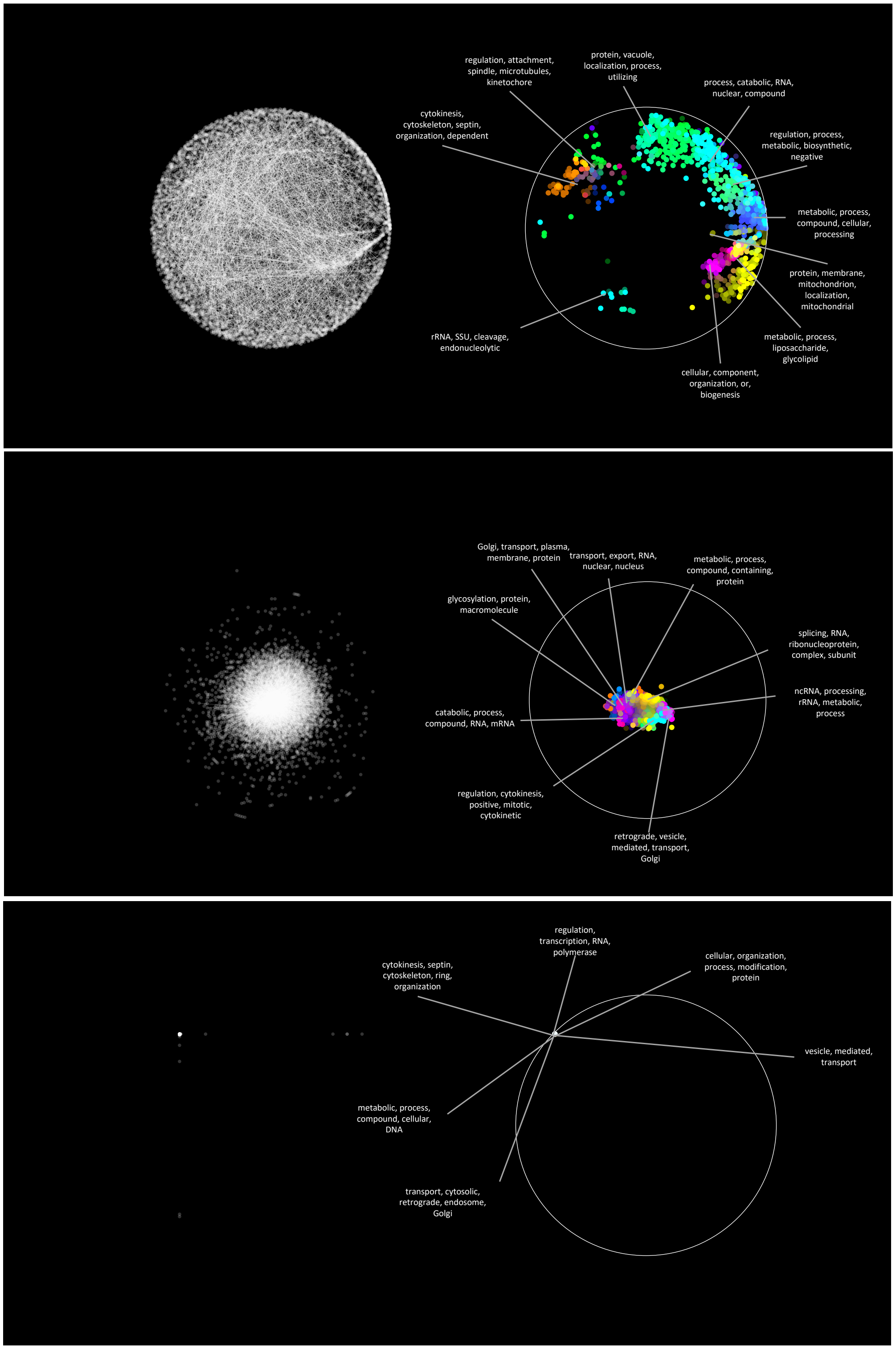

Supplementary Figure 11: **Functional landscape of the Budding yeast GI network for different types of network embedding based on graphlet adjacency $\widetilde{A}_{G_0}$.** We use SAFE to annotate the Budding yeast GI network with GO-BP for (Top) GraCoal embedding, (Center) Spring embedding and (Bottom) Spectral embedding. For each type of network embedding, we show the network embedding on the left and the SAFE enrichment domains highlighted in colour on the right.

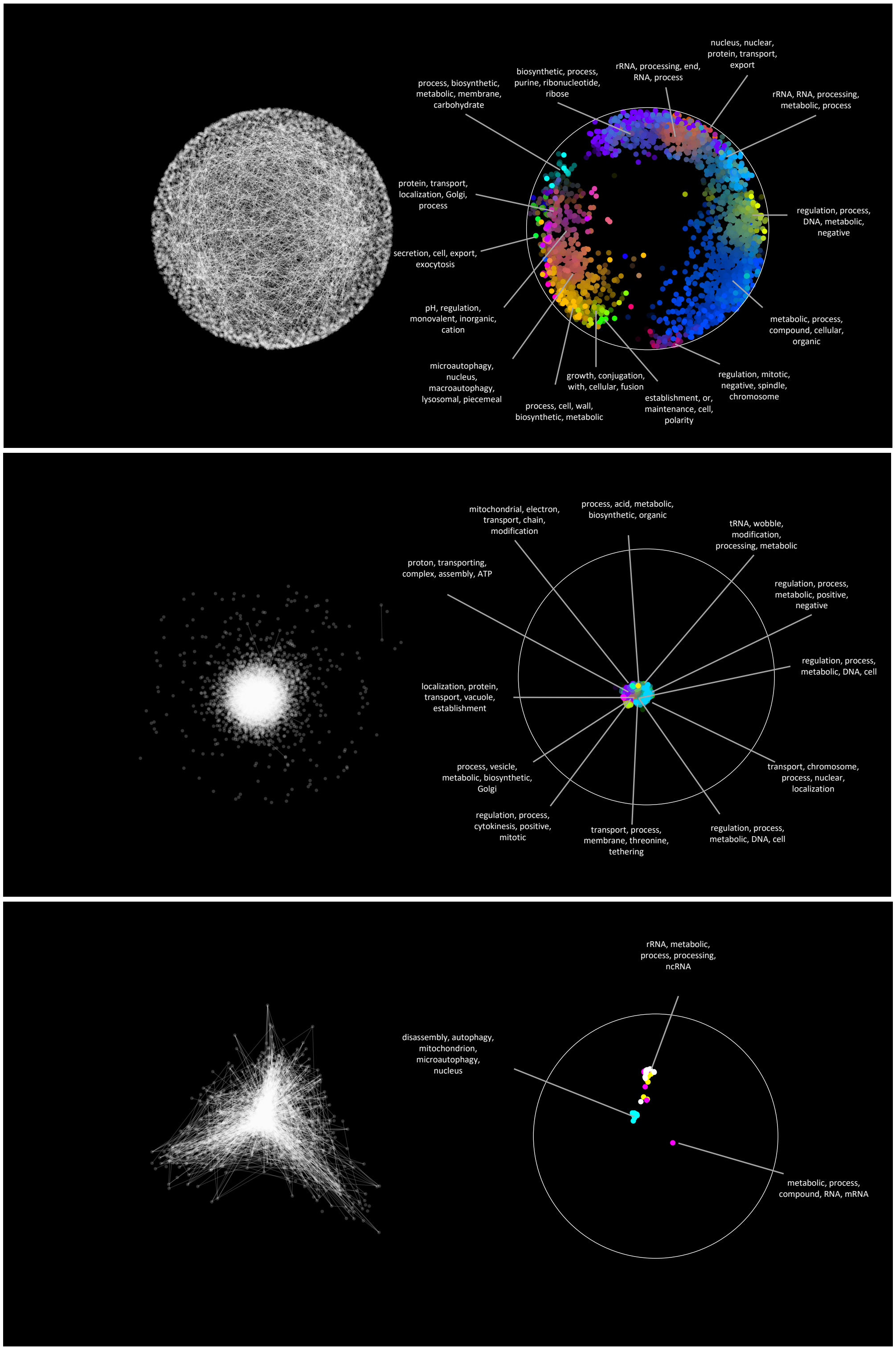

Supplementary Figure 12: **Functional landscape of the Budding yeast GI network for different types of network embedding based on graphlet adjacency $\widetilde{A}_{G_2}$.** We use SAFE to annotate the Budding yeast GI network with GO-BP for (Top) GraCoal embedding, (Center) Spring embedding and (Bottom) Spectral embedding. For each type of network embedding, we show the network embedding on the left and the SAFE enrichment domains highlighted in colour on the right.

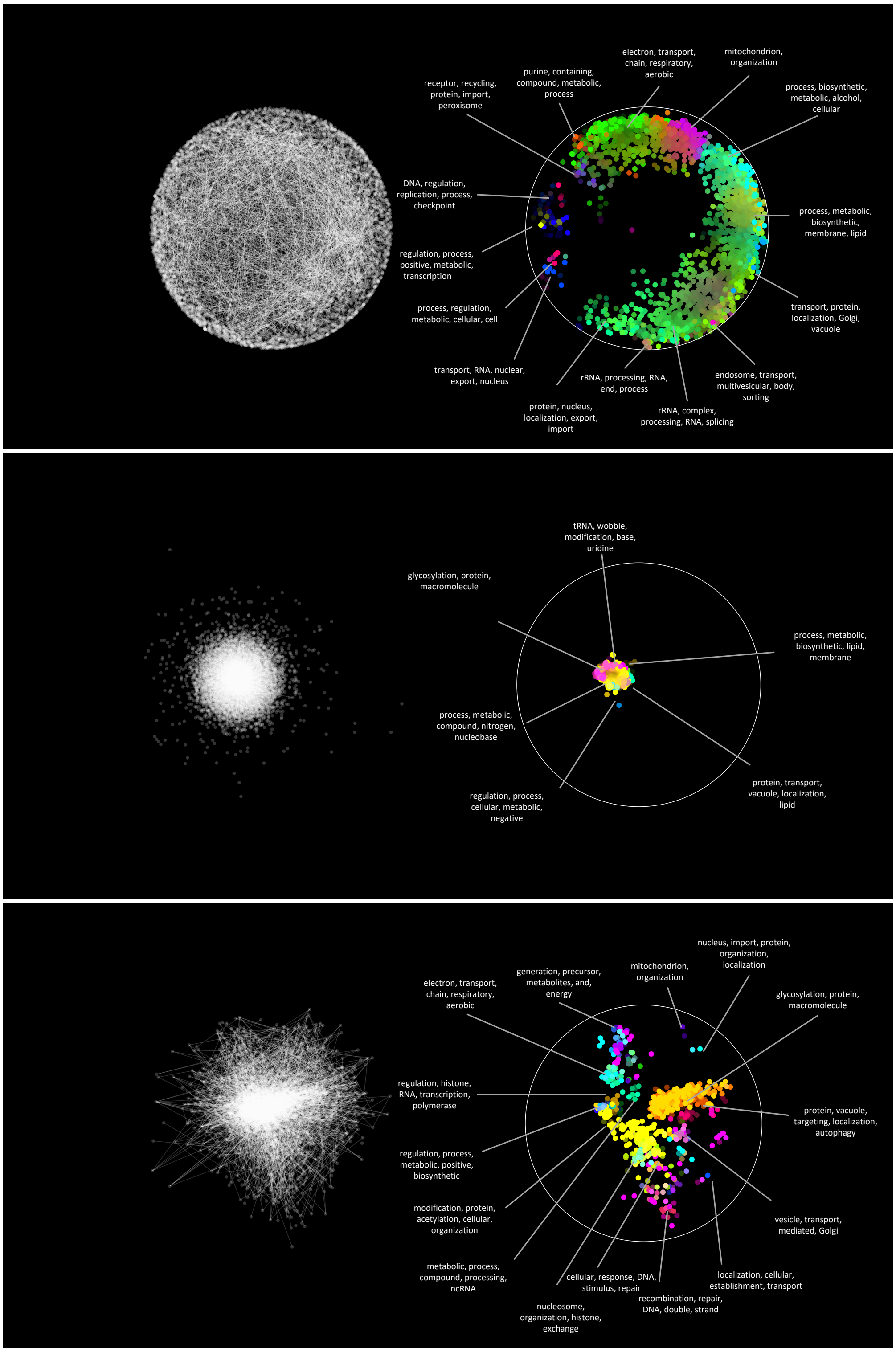

Supplementary Figure 13: **Functional landscape of the Budding yeast GI network for different types of network embedding based on graphlet adjacency A_{G_3}.** We use SAFE to annotate the Budding yeast GI network with GO-BP for (Top) GraCoal embedding, (Center) Spring embedding and (Bottom) Spectral embedding. For each type of network embedding, we show the network embedding on the left and the SAFE enrichment domains highlighted in colour on the right.

$\widetilde{A}_{G_i}$	**Organism**	**GraCoal**	**GraSpring**	**Spectral**
$\widetilde{A}_{G_0}$	Budding yeast	0.57 (std=0.27)	0.16 (std=0.09)	0.00 (std=0.03)
$\widetilde{A}_{G_1}$	Budding yeast	0.57 (std=0.27)	0.14 (std=0.08)	0.07 (std=0.06)
$\widetilde{A}_{G_2}$	Budding yeast	0.57 (std=0.27)	0.18 (std=0.10)	0.11 (std=0.11)
$\widetilde{A}_{G_3}$	Budding yeast	0.57 (std=0.27)	0.13 (std=0.07)	0.16 (std=0.11)
$\widetilde{A}_{G_4}$	Budding yeast	0.57 (std=0.27)	0.11 (std=0.06)	0.02 (std=0.03)
$\widetilde{A}_{G_5}$	Budding yeast	0.57 (std=0.27)	0.15 (std=0.09)	0.09 (std=0.09)
$\widetilde{A}_{G_6}$	Budding yeast	0.57 (std=0.27)	0.13 (std=0.08)	0.08 (std=0.07)
$\widetilde{A}_{G_7}$	Budding yeast	0.57 (std=0.27)	0.18 (std=0.10)	0.10 (std=0.10)
$\widetilde{A}_{G_8}$	Budding yeast	0.57 (std=0.27)	0.23 (std=0.13)	0.05 (std=0.08)
$\widetilde{A}_{G_0}$	*E. coli*	0.57 (std=0.27)	0.35 (std=0.17)	0.16 (std=0.14)
$\widetilde{A}_{G_1}$	*E. coli*	0.57 (std=0.27)	0.30 (std=0.15)	0.11 (std=0.10)
$\widetilde{A}_{G_2}$	*E. coli*	0.56 (std=0.27)	0.28 (std=0.14)	0.13 (std=0.14)
$\widetilde{A}_{G_3}$	*E. coli*	0.57 (std=0.27)	0.27 (std=0.14)	0.16 (std=0.14)
$\widetilde{A}_{G_4}$	*E. coli*	0.57 (std=0.27)	0.21 (std=0.10)	0.06 (std=0.05)
$\widetilde{A}_{G_5}$	*E. coli*	0.56 (std=0.27)	0.24 (std=0.12)	0.16 (std=0.16)
$\widetilde{A}_{G_6}$	*E. coli*	0.57 (std=0.27)	0.23 (std=0.11)	0.12 (std=0.11)
$\widetilde{A}_{G_7}$	*E. coli*	0.56 (std=0.27)	0.30 (std=0.15)	0.11 (std=0.13)
$\widetilde{A}_{G_8}$	*E. coli*	0.56 (std=0.27)	0.26 (std=0.14)	0.11 (std=0.14)
$\widetilde{A}_{G_0}$	Fission yeast	0.57 (std=0.27)	0.22 (std=0.12)	0.00 (std=0.03)
$\widetilde{A}_{G_1}$	Fission yeast	0.57 (std=0.27)	0.21 (std=0.12)	0.02 (std=0.04)
$\widetilde{A}_{G_2}$	Fission yeast	0.56 (std=0.27)	0.28 (std=0.15)	0.01 (std=0.06)
$\widetilde{A}_{G_3}$	Fission yeast	0.57 (std=0.27)	0.16 (std=0.09)	0.05 (std=0.07)
$\widetilde{A}_{G_4}$	Fission yeast	0.57 (std=0.27)	0.14 (std=0.09)	0.03 (std=0.04)
$\widetilde{A}_{G_5}$	Fission yeast	0.57 (std=0.27)	0.21 (std=0.12)	0.01 (std=0.08)
$\widetilde{A}_{G_6}$	Fission yeast	0.57 (std=0.27)	0.17 (std=0.10)	0.05 (std=0.08)
$\widetilde{A}_{G_7}$	Fission yeast	0.56 (std=0.27)	0.26 (std=0.14)	0.01 (std=0.05)
$\widetilde{A}_{G_8}$	Fission yeast	0.56 (std=0.27)	0.28 (std=0.15)	0.01 (std=0.07)
$\widetilde{A}_{G_0}$	Fruit fly	0.57 (std=0.27)	0.27 (std=0.14)	0.00 (std=0.04)
$\widetilde{A}_{G_1}$	Fruit fly	0.57 (std=0.27)	0.21 (std=0.11)	0.01 (std=0.05)
$\widetilde{A}_{G_2}$	Fruit fly	0.56 (std=0.26)	0.20 (std=0.12)	0.01 (std=0.07)
$\widetilde{A}_{G_3}$	Fruit fly	0.57 (std=0.27)	0.21 (std=0.11)	0.05 (std=0.06)
$\widetilde{A}_{G_4}$	Fruit fly	0.57 (std=0.27)	0.19 (std=0.11)	0.01 (std=0.06)
$\widetilde{A}_{G_5}$	Fruit fly	0.56 (std=0.26)	0.24 (std=0.13)	0.02 (std=0.09)
$\widetilde{A}_{G_6}$	Fruit fly	0.56 (std=0.27)	0.28 (std=0.14)	0.02 (std=0.06)
$\widetilde{A}_{G_7}$	Fruit fly	0.56 (std=0.26)	0.19 (std=0.11)	0.02 (std=0.09)
$\widetilde{A}_{G_8}$	Fruit fly	0.55 (std=0.26)	0.19 (std=0.12)	0.03 (std=0.12)

Supplementary Table 6: **Normalized average Euclidean distance between pairs of nodes in graphlet-based embeddings.** For each of our GI molecular networks (rows), we report the average Euclidean distance (normalized by the largest distance in each embedding method and each species) between all pairs of nodes across all graphlet adjacencies (i.e., $\widetilde{A}_{G_0}$ - $\widetilde{A}_{G_8}$) for GraCoal, GraSpring and graphlet-based Spectral embeddings (columns 1-3).

3.4 Enrichment statistics

In this section, we summarize the results obtained when using SAFE with the different graphlet-based embedding algorithms. That is, the percentages of genes that have at least one annotation enriched in their neighborhood and the percentages of enriched annotations for all our GI and PPI molecular networks across all annotation types.

3.4.1 Gene ontology biological processes enrichment statistics

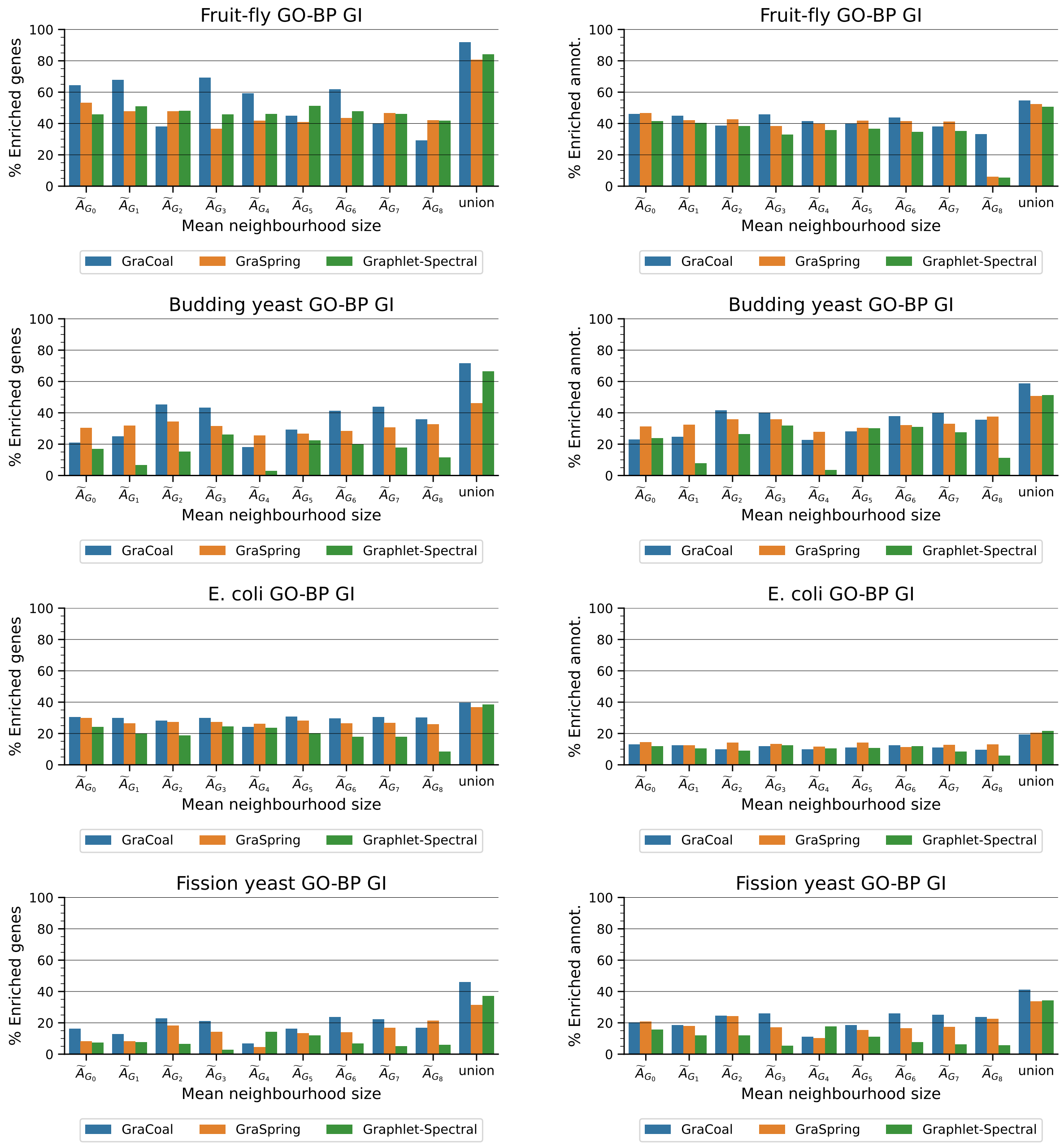

Supplementary Figure 14: **SAFE GO-BP enrichment analysis for the GI networks.** For each of the four GI networks (rows), we show for the different graphlet adjacencies (x-axis) for each of the graphlet-based embedding methods considered (color coded), the percentages of genes that have at least one annotation enriched in their neighborhood (left sub-plot, y-axis) and the percentages of enriched annotations (right sub-plot, y-axis). 'Union' (x-axis, far right) considers the union of the enriched genes and the union of the enriched annotations , across all of the graphlet adjacencies. For GraSpring, the reported enrichment scores are the average scores over ten runs, with the error bars indicating their standard deviation.

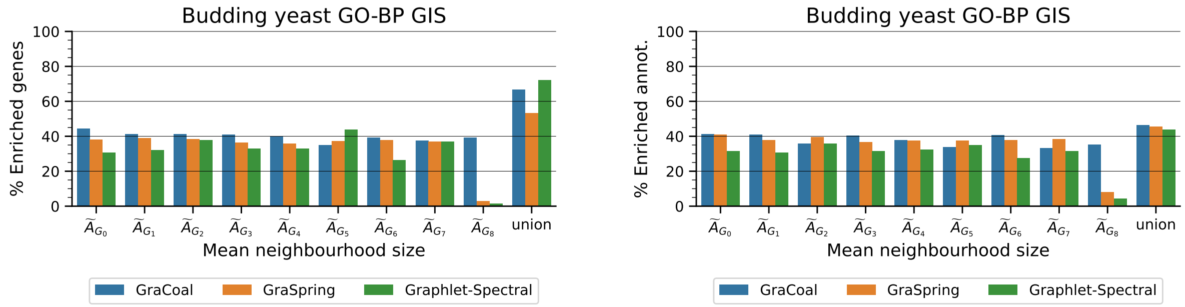

Supplementary Figure 15: **SAFE GO-BP enrichment analysis for the Budding yeast GIS network.** For the Budding yeast GIS network, we show for the different graphlet adjacencies (x-axis) for each of the graphlet-based embedding methods considered (color coded), the percentages of genes that have at least one annotation enriched in their neighborhood (left sub-plot, y-axis) and the percentages of enriched annotations (right sub-plot, y-axis). 'Union' (x-axis, far right) considers the union of the enriched genes and the union of the enriched annotations , across all of the graphlet adjacencies. For GraSpring, the reported enrichment scores are the average scores over ten runs, with the error bars indicating their standard deviation.

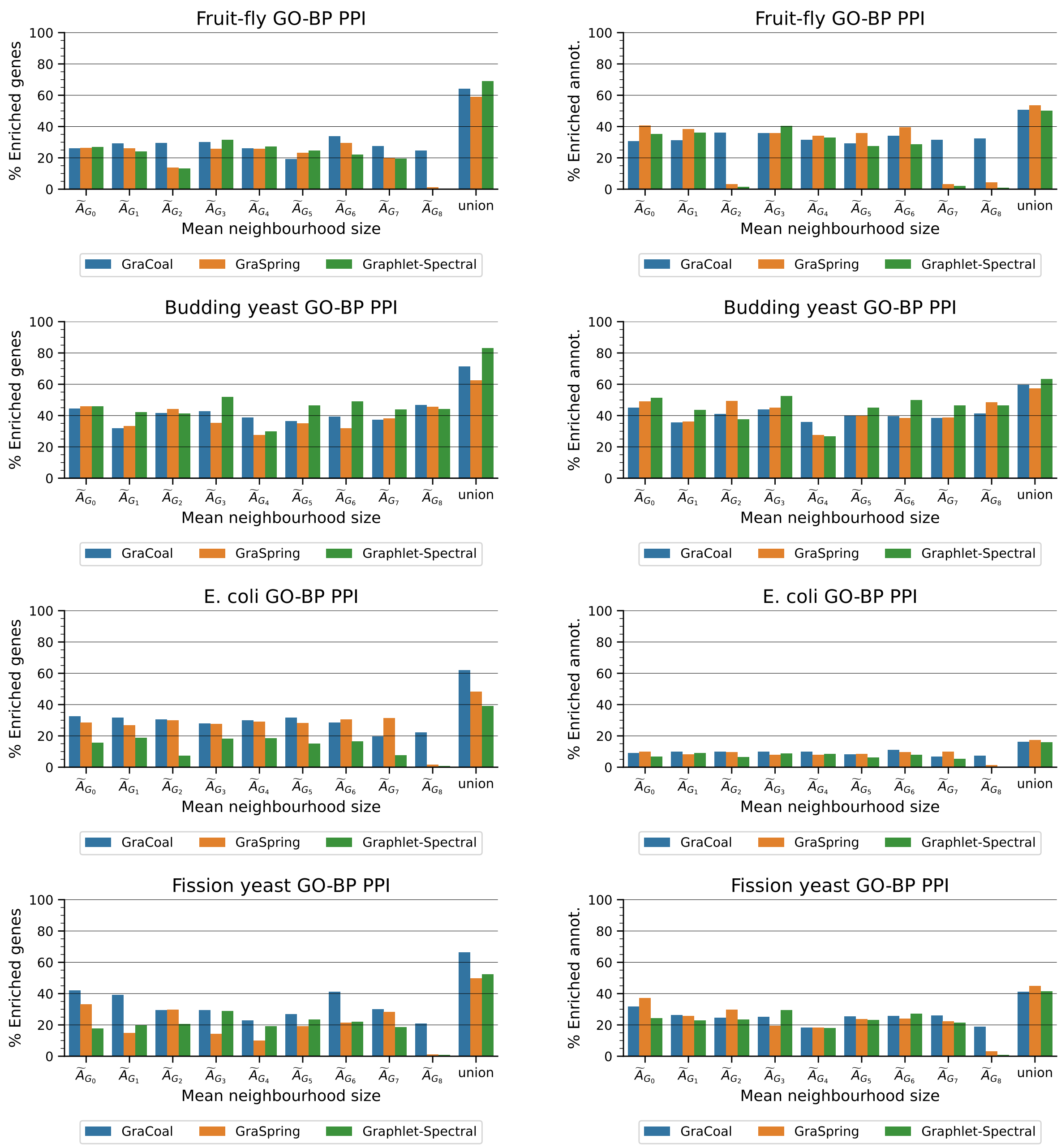

Supplementary Figure 16: **SAFE GO-BP enrichment analysis for the PPI networks, Part 1.** For each of the six PPI networks (rows), we show for the different graphlet adjacencies (x-axis) for each of the graphlet-based embedding methods considered (color coded), the percentages of genes that have at least one annotation enriched in their neighborhood (left sub-plot, y-axis) and the percentages of enriched annotations (right sub-plot, y-axis). 'Union' (x-axis, far right) considers the union of the enriched genes and the union of the enriched annotations , across all of the graphlet adjacencies. For GraSpring, the reported enrichment scores are the average scores over ten runs, with the error bars indicating their standard deviation.

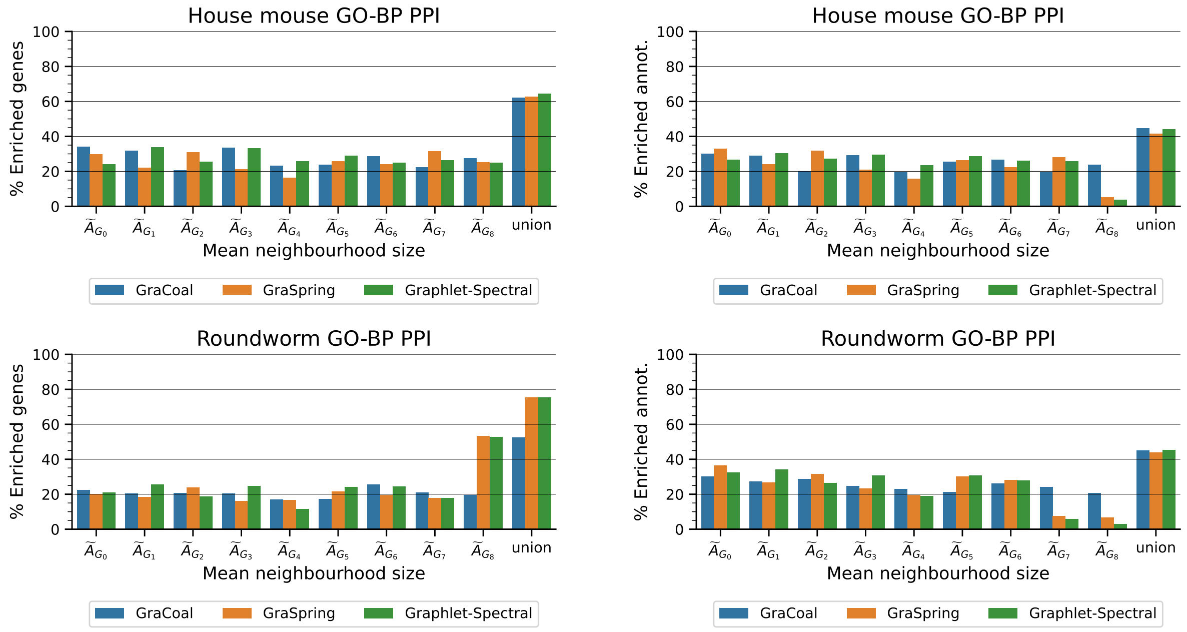

Supplementary Figure 16: **SAFE GO-BP enrichment analysis for the PPI networks, Part 2.** For each of the six PPI networks (rows), we show for the different graphlet adjacencies (x-axis) for each of the graphlet-based embedding methods considered (color coded), the percentages of genes that have at least one annotation enriched in their neighborhood (left sub-plot, y-axis) and the percentages of enriched annotations (right sub-plot, y-axis). 'Union' (x-axis, far right) considers the union of the enriched genes and the union of the enriched annotations , across all of the graphlet adjacencies. For GraSpring, the reported enrichment scores are the average scores over ten runs, with the error bars indicating their standard deviation.

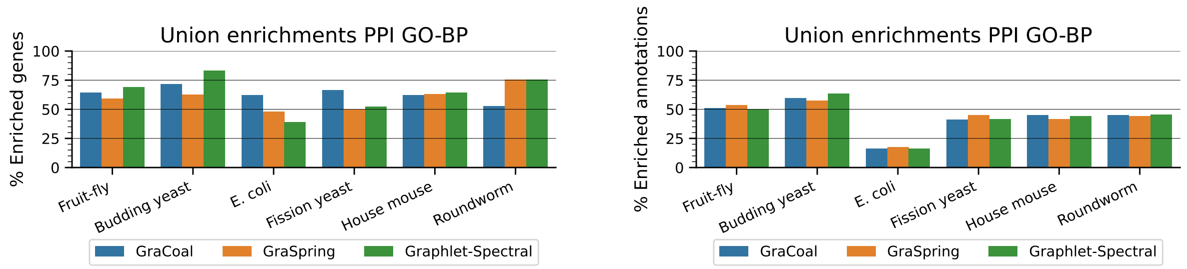

Supplementary Figure 17: **SAFE GO-BP enrichment analysis for PPI networks.** For the PPI networks of the six species (x-axis), we show the union of the percentages of enriched genes (y-axis) and union of the percentages of enriched annotations for each of the embedding algorithms considered (color coded). The error bars in the case of GraSpring embedding indicate the standard deviation across the ten randomised runs.

3.4.2 Gene ontology cellular components enrichment statistics

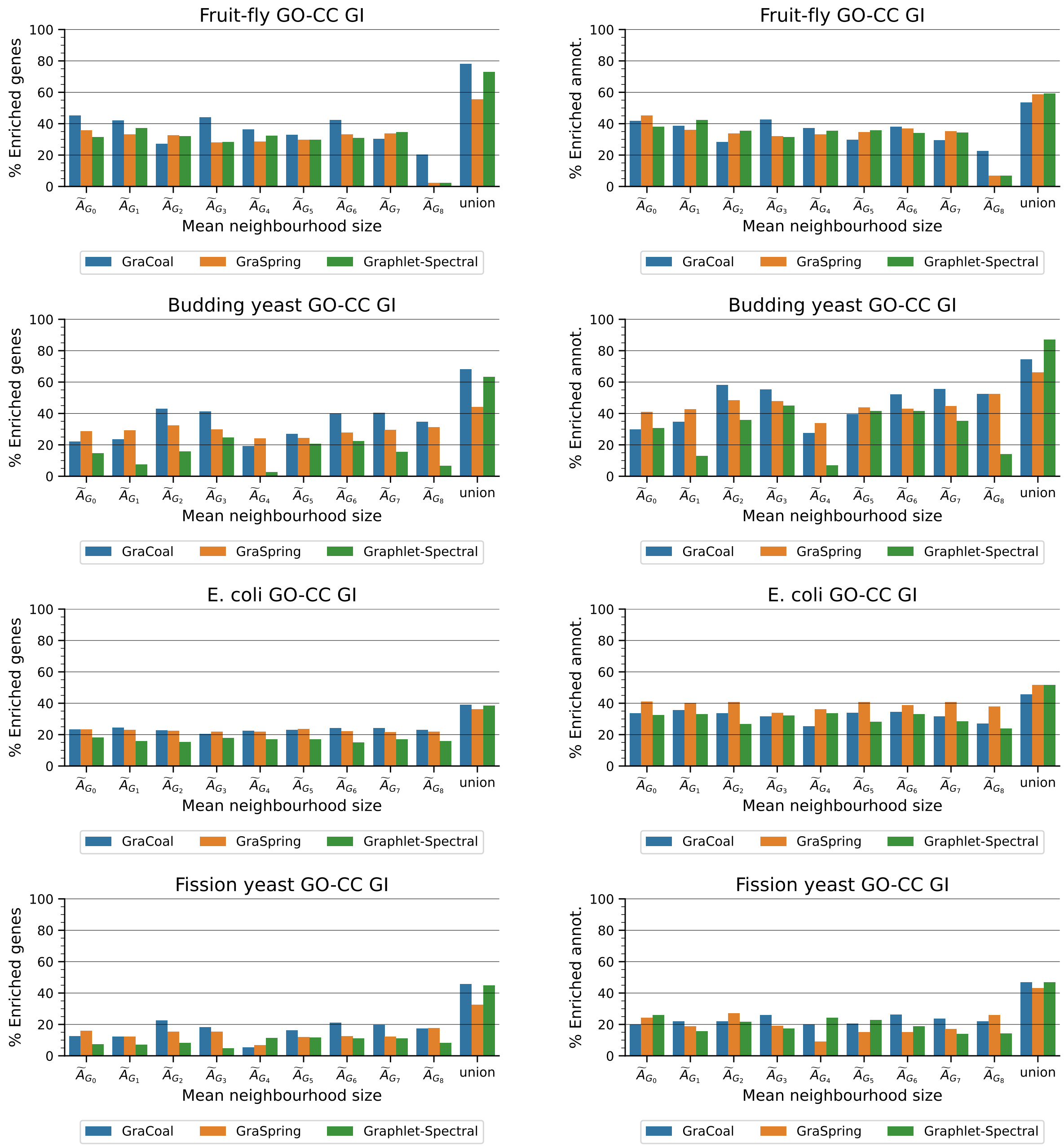

Supplementary Figure 18: **SAFE GO-CC enrichment analysis for the GI networks.** For each of the four GI networks (rows), we show for the different graphlet adjacencies (x-axis) for each of the graphlet-based embedding methods considered (color coded), the percentages of genes that have at least one annotation enriched in their neighborhood (left sub-plot, y-axis) and the percentages of enriched annotations (right sub-plot, y-axis). 'Union' (x-axis, far right) considers the union of the enriched genes and the union of the enriched annotations , across all of the graphlet adjacencies. For GraSpring, the reported enrichment scores are the average scores over ten runs, with the error bars indicating their standard deviation.

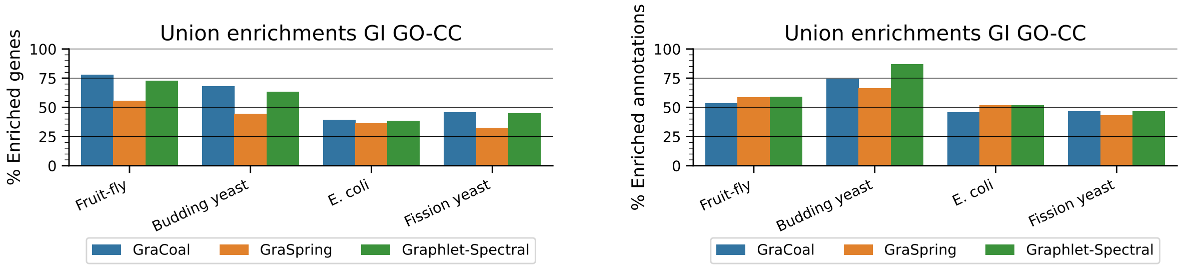

Supplementary Figure 19: **SAFE GO-CC enrichment analysis for GI networks.** For the GI networks of the four species (x-axis), we show the union of the percentages of enriched genes (y-axis) and union of the percentages of enriched annotations for each of the embedding algorithms considered (color coded). The error bars in the case of GraSpring embedding indicate the standard deviation across the ten randomised runs.

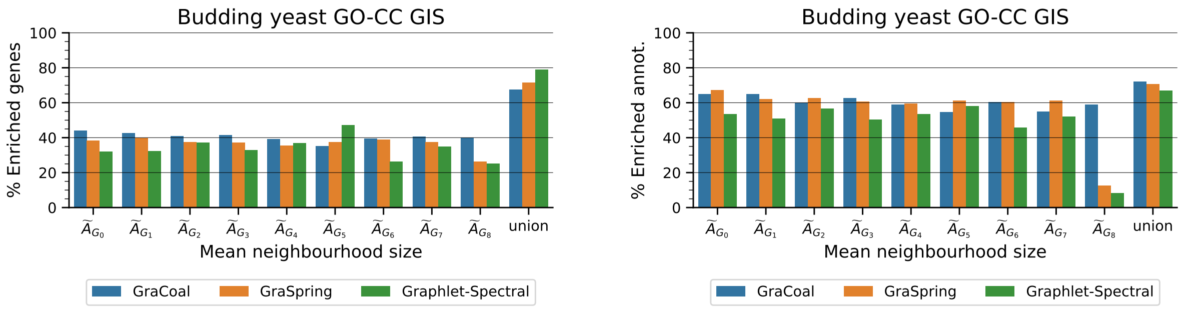

Supplementary Figure 20: **SAFE GO-CC enrichment analysis for the Budding yeast GIS network.** For the Budding yeast GIS network, we show for the different graphlet adjacencies (x-axis) for each of the graphlet-based embedding methods considered (color coded), the percentages of genes that have at least one annotation enriched in their neighborhood (left sub-plot, y-axis) and the percentages of enriched annotations (right sub-plot, y-axis). 'Union' (x-axis, far right) considers the union of the enriched genes and the union of the enriched annotations , across all of the graphlet adjacencies. For GraSpring, the reported enrichment scores are the average scores over ten runs, with the error bars indicating their standard deviation.

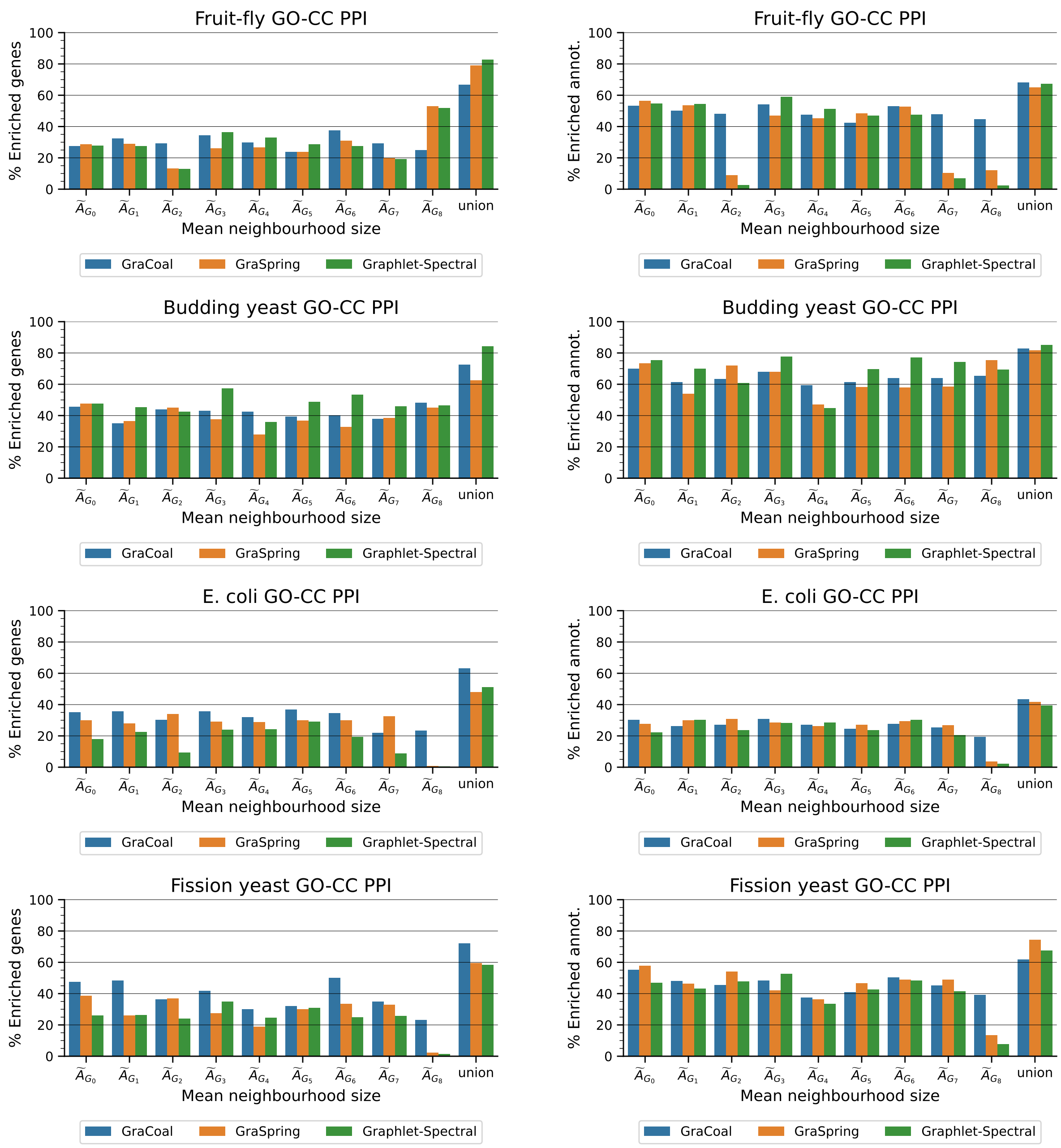

Supplementary Figure 21: **SAFE GO-CC enrichment analysis for the PPI networks, Part 1.** For each of the six PPI networks (rows), we show for the different graphlet adjacencies (x-axis) for each of the graphlet-based embedding methods considered (color coded), the percentages of genes that have at least one annotation enriched in their neighborhood (left sub-plot, y-axis) and the percentages of enriched annotations (right sub-plot, y-axis). 'Union' (x-axis, far right) considers the union of the enriched genes and the union of the enriched annotations , across all of the graphlet adjacencies. For GraSpring, the reported enrichment scores are the average scores over ten runs, with the error bars indicating their standard deviation.

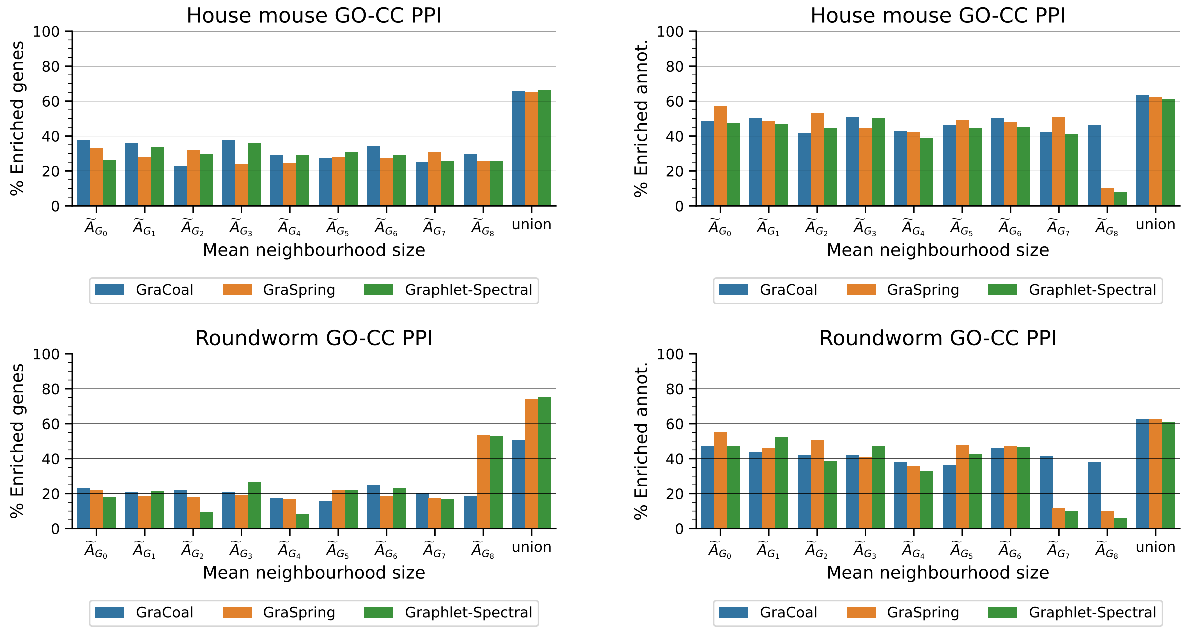

Supplementary Figure 21: **SAFE GO-CC enrichment analysis for the PPI networks, Part 2.** For each of the six PPI networks (rows), we show for the different graphlet adjacencies (x-axis) for each of the graphlet-based embedding methods considered (color coded), the percentages of genes that have at least one annotation enriched in their neighborhood (left sub-plot, y-axis) and the percentages of enriched annotations (right sub-plot, y-axis). 'Union' (x-axis, far right) considers the union of the enriched genes and the union of the enriched annotations , across all of the graphlet adjacencies. For GraSpring, the reported enrichment scores are the average scores over ten runs, with the error bars indicating their standard deviation.

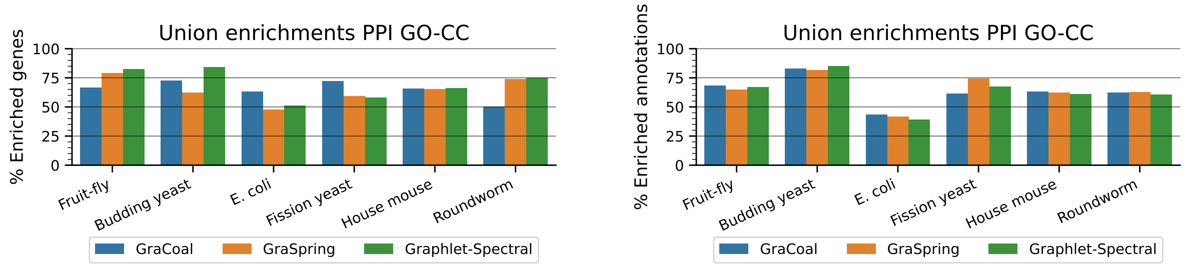

Supplementary Figure 22: **SAFE GO-CC enrichment analysis for PPI networks.** For the PPI networks of the six species (x-axis), we show the union of the percentages of enriched genes (y-axis) and the union of the percentages of enriched annotations for each of the embedding algorithms considered (color coded). The error bars in the case of GraSpring embedding indicate the standard deviation across the ten randomised runs.

3.4.3 Gene ontology molecular functions enrichment statistics

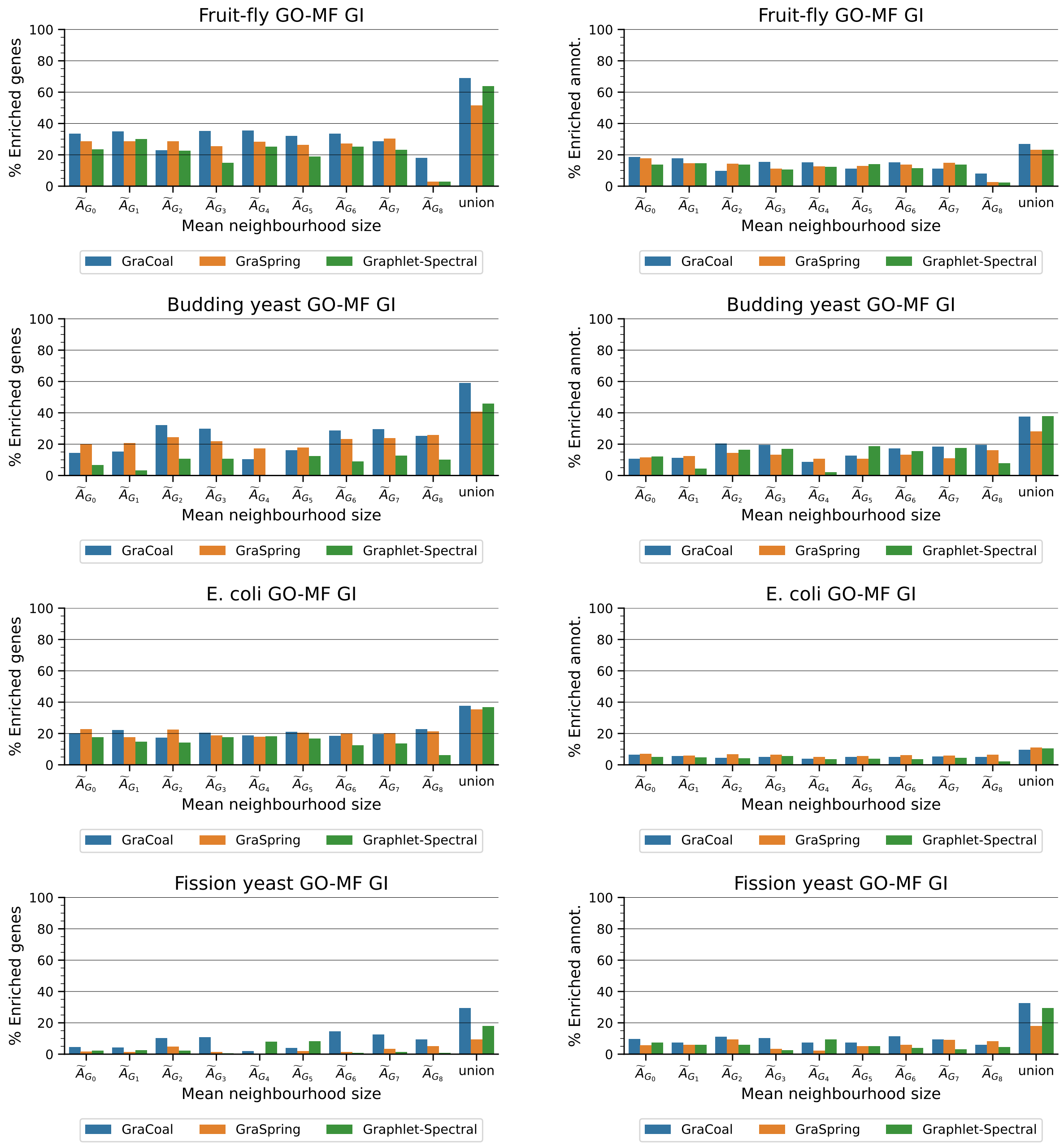

Supplementary Figure 23: **SAFE GO-MF enrichment analysis for the GI networks.** For each of the four GI networks (rows), we show for the different graphlet adjacencies (x-axis) for each of the graphlet-based embedding methods considered (color coded), the percentages of genes that have at least one annotation enriched in their neighborhood (left sub-plot, y-axis) and the percentages of enriched annotations (right sub-plot, y-axis). ‘Union’ (x-axis, far right) considers the union of the enriched genes and the union of the enriched annotations , across all of the graphlet adjacencies.

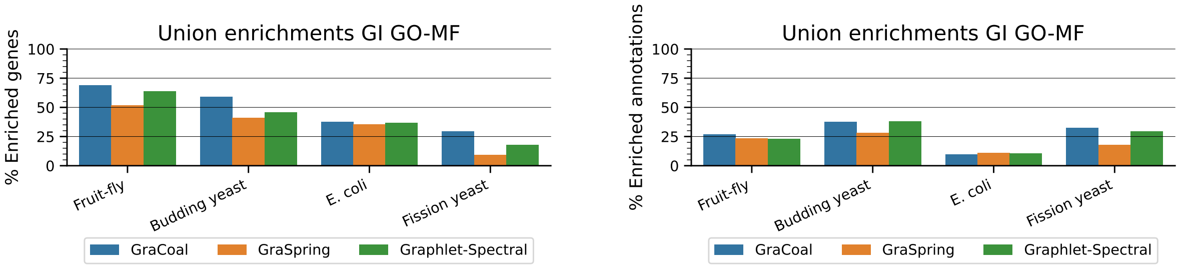

Supplementary Figure 24: **SAFE GO-MF enrichment analysis for GI networks.** For the GI networks of the four species (x-axis), we show the union of the percentages of enriched genes (y-axis) and the union of the percentages of enriched annotations for each of the embedding algorithms considered (color coded). The error bars in the case of GraSpring embedding indicate the standard deviation across the ten randomised runs.

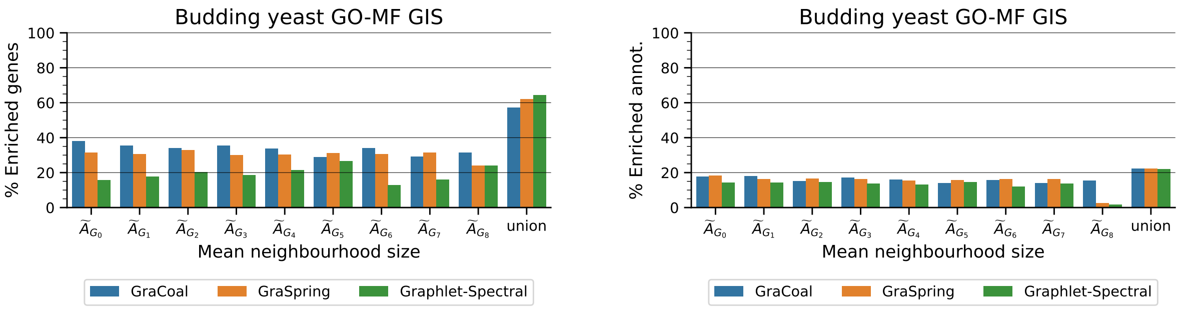

Supplementary Figure 25: **SAFE GO-MF enrichment analysis for the Budding yeast GIS network.** For the Budding yeast GIS network, we show for the different graphlet adjacencies (x-axis) for each of the graphlet-based embedding methods considered (color coded), the percentages of genes that have at least one annotation enriched in their neighborhood (left sub-plot, y-axis) and the percentages of enriched annotations (right sub-plot, y-axis). 'Union' (x-axis, far right) considers the union of the enriched genes and the union of the enriched annotations , across all of the graphlet adjacencies. For GraSpring, the reported enrichment scores are the average scores over ten runs, with the error bars indicating their standard deviation.

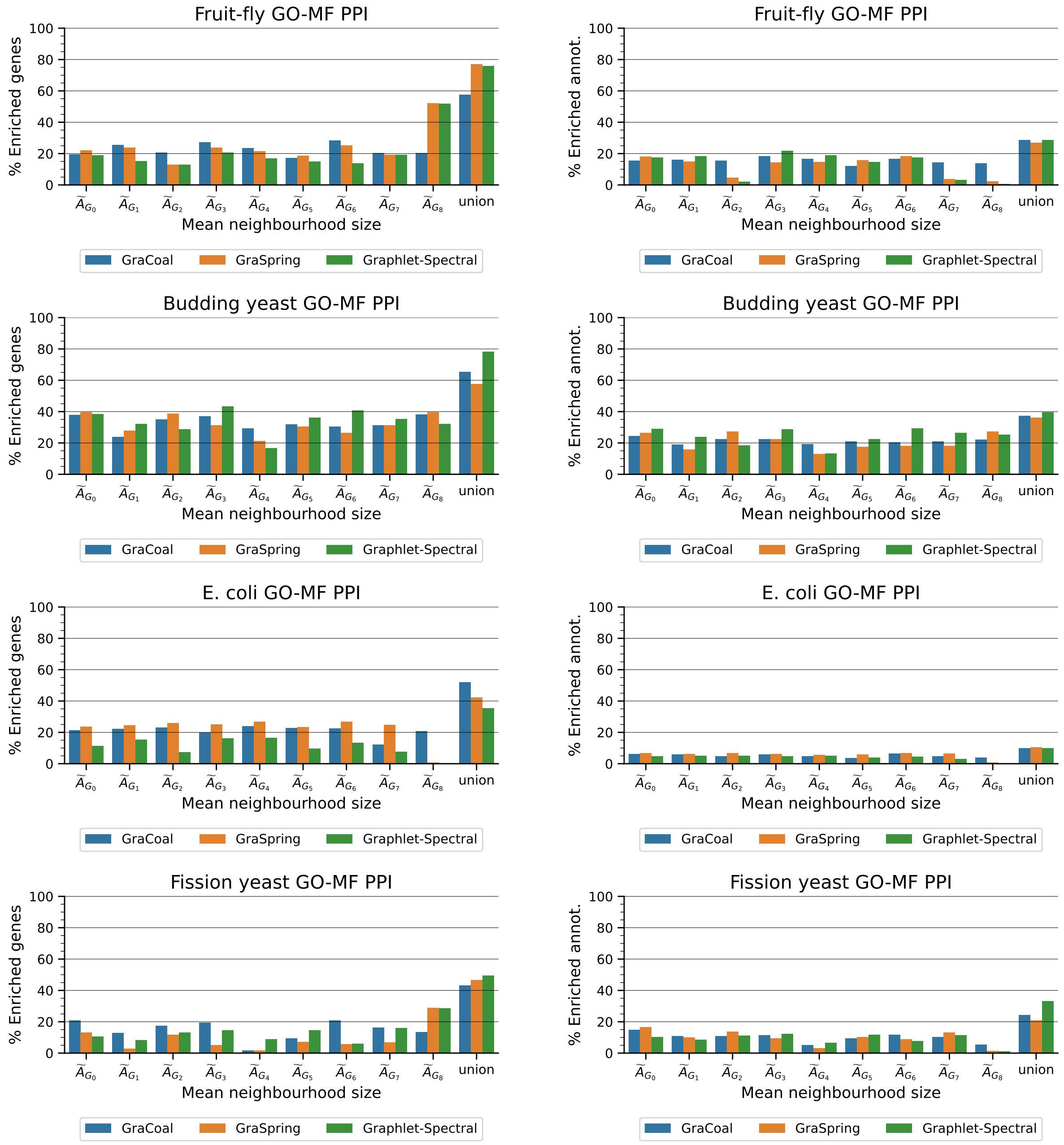

Supplementary Figure 26: **SAFE GO-MF enrichment analysis for the PPI networks, Part 1.** For each of the six PPI networks (rows), we show for the different graphlet adjacencies (x-axis) for each of the graphlet-based embedding methods considered (color coded), the percentages of genes that have at least one annotation enriched in their neighborhood (left sub-plot, y-axis) and the percentages of enriched annotations (right sub-plot, y-axis). ‘Union’ (x-axis, far right) considers the union of the enriched genes and the union of the enriched annotations , across all of the graphlet adjacencies. For GraSpring, the reported enrichment scores are the average scores over ten runs, with the error bars indicating their standard deviation.

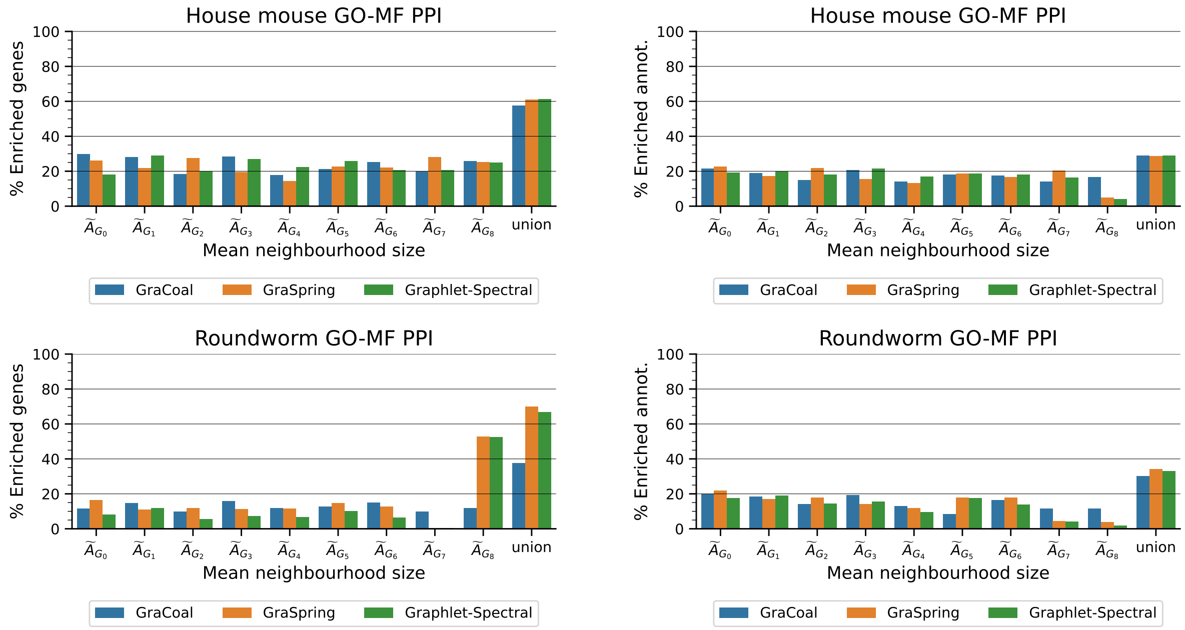

Supplementary Figure 26: **SAFE GO-MF enrichment analysis for the PPI networks, Part 2.** For each of the six PPI networks (rows), we show for the different graphlet adjacencies (x-axis) for each of the graphlet-based embedding methods considered (color coded), the percentages of genes that have at least one annotation enriched in their neighborhood (left sub-plot, y-axis) and the percentages of enriched annotations (right sub-plot, y-axis). 'Union' (x-axis, far right) considers the union of the enriched genes and the union of the enriched annotations , across all of the graphlet adjacencies. For GraSpring, the reported enrichment scores are the average scores over ten runs, with the error bars indicating their standard deviation.

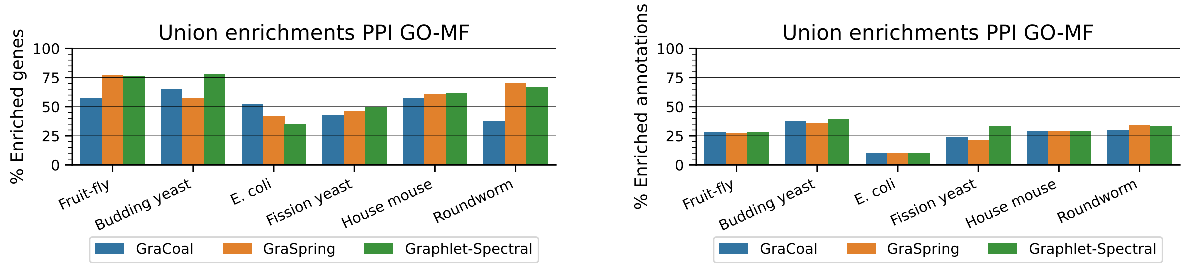

Supplementary Figure 27: **SAFE GO-MF enrichment analysis for PPI networks.** For the PPI networks of the six species (x-axis), we show the union of the percentages of enriched genes (y-axis) and the union of the percentages of enriched annotations for each of the embedding algorithms considered (color coded). The error bars in the case of GraSpring embedding indicate the standard deviation across the ten randomised runs.

3.4.4 Comparing GO-BP enrichment statistics between different species

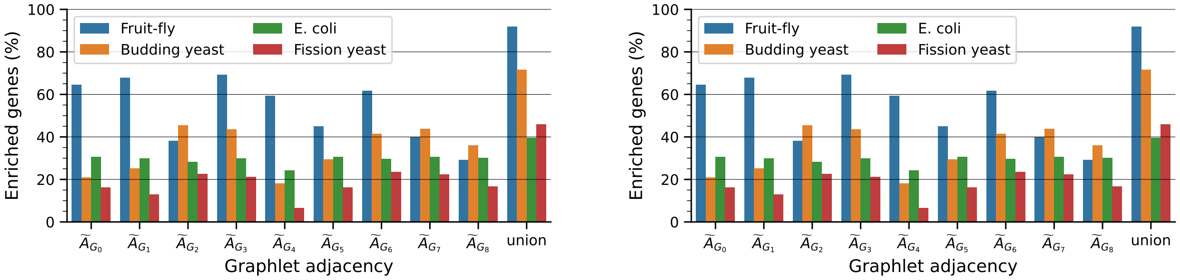

Supplementary Figure 28: **SAFE GO-BP enrichment analysis comparing GraCoals in GI networks.** For the GI networks of the four species (color coded), we show, on the y-axis, the percentage of enriched genes (left hand side) and percentage of enriched GO-BPs (right hand side) for each of the different Gracoal embeddings (x-axis).

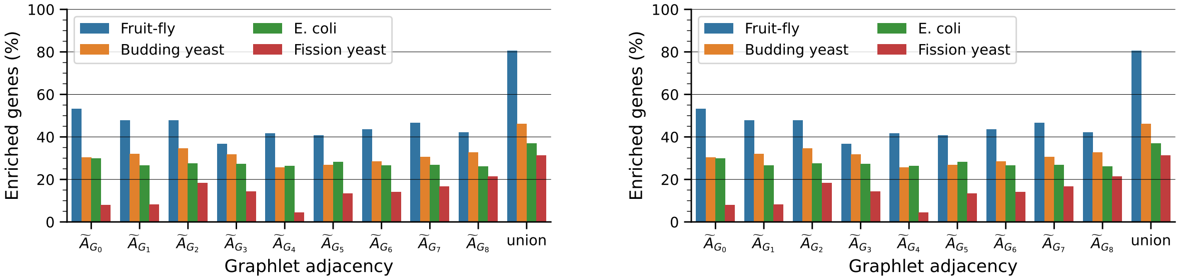

Supplementary Figure 29: **SAFE GO-BP enrichment analysis comparing GraSprings in GI networks.** For the GI networks of the four species (color coded), we show, on the y-axis, the percentage of enriched genes (left hand side) and percentage of enriched GO-BPs (right hand side) for each of the different GraSpring embeddings (x-axis).

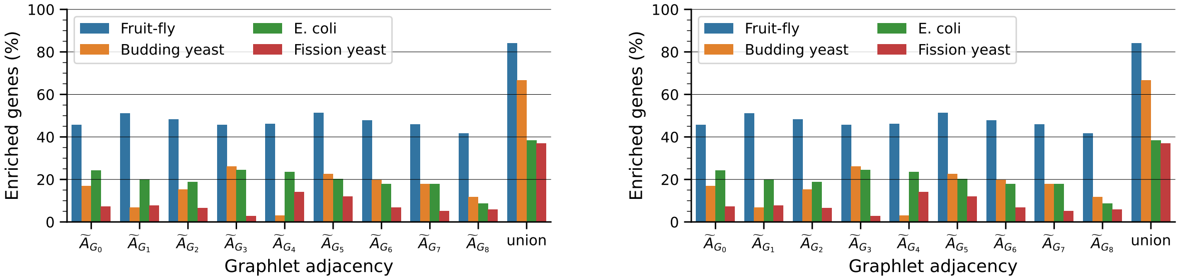

Supplementary Figure 30: **SAFE GO-BP enrichment analysis comparing graphlet-based Spectral embeddings in GI networks.** For the GI networks of the four species (color coded), we show, on the y-axis, the percentage of enriched genes (left hand side) and the percentage of enriched GO-BPs (right hand side) for each of the different graphlet-based Spectral embeddings (x-axis).

3.5 Model fitting

We perform model fitting experiments to characterize the structure of our molecular networks (i.e., the GI and PPI molecular networks for different model organisms as well as the Budding yeast GIS network) by comparing the molecular networks to eight different types of random model networks commonly used in biology. For the GI networks and the budding yeast GIS network, we observe none are well fitted by the ER model (Supplementary Figure 31), meaning the topology of these networks is not random. Similarly, for the PPI networks, we observe that none have random topology, as they are not well fitted by the ER model (Supplementary Figure 32). Moreover, for the GI networks, SF-GD appears to be overall the best fit, as evidenced by the lowest GCD-11 distances between the real networks and the SF-GD model networks (Supplementary Figure 31). Indeed, we observe that the distribution of model to model GCD-11 distances and the distribution of real to model GCD-11 distances overlap for three of the four GI networks (Supplementary Figure 33). To assess if there is a difference between these two sets of distances for all model networks and real molecular networks, we perform a MWU test. In Supplementary Table 7 and Supplementary Table 8 we show the p-values of the MWU test computed for all the GI networks and PPI networks, respectively. Even though the *E. coli* GI network is the only network that cannot be differentiated from the SFGD model, it is still the best fit for the budding yeast and the fission yeast GI networks (as evidenced by the overlap of the GCD-11 distances distributions) in Supplementary Figure 33.

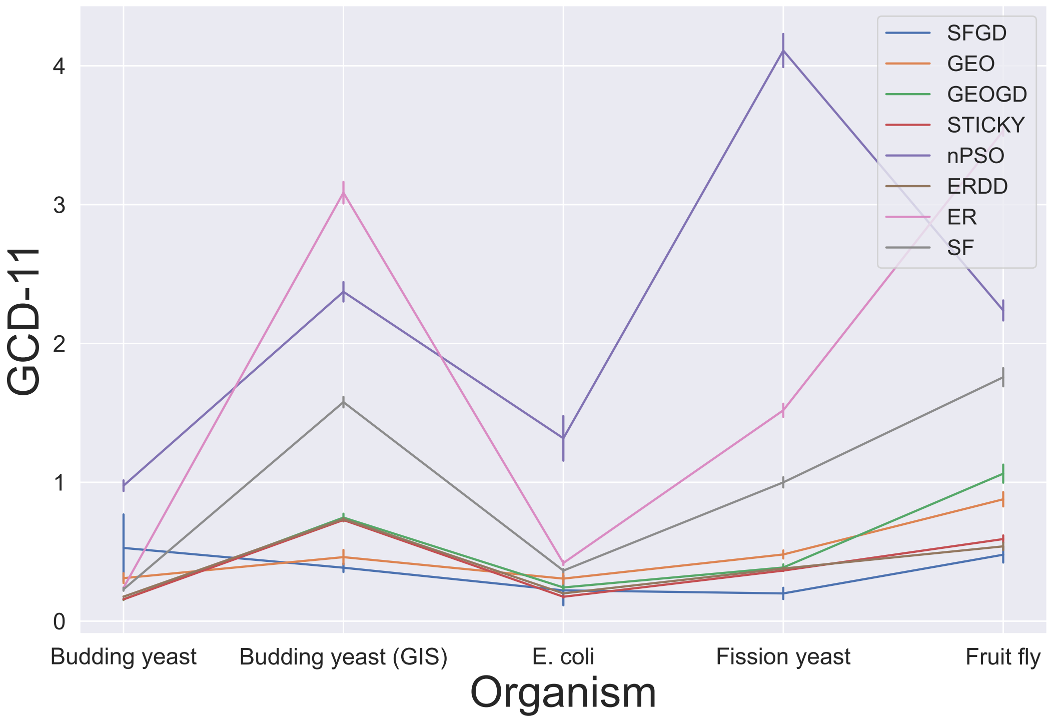

Supplementary Figure 31: **Fit of network models for the GI networks.** Each line shows the fitting of a network model for the different GI networks and the Budding yeast GIS network (x-axis). The error-bars indicate the standard deviations of the pairwise GCD-11 distances (y-axis) between the GI networks and 15 randomly generated networks corresponding to each network model.

Organism	ER	ERDD	GEO	GEOGD	nPSO	SF	SFGD	STICKY
**Budding yeast**	1.10E-06	1.10E-06	1.10E-06	1.10E-06	1.10E-06	1.10E-06	1.79E-02	1.10E-06
E. coli	1.10E-06	1.10E-06	1.10E-06	1.10E-06	1.10E-06	1.10E-06	**5.20E-01**	1.10E-06
**Fission yeast**	1.10E-06	1.10E-06	1.10E-06	1.10E-06	1.10E-06	1.10E-06	1.61E-02	1.10E-06
**Fruit fly**	9.63E-07	9.63E-07	9.63E-07	9.63E-07	9.63E-07	9.63E-07	9.63E-07	9.63E-07

Supplementary Table 7: **Mann-Whitney-U test p-values for model network fitting of GI networks.** For each random model, we perform a MWU test between two distance distributions to evaluate if there is any statistical difference: GCD-11 between the real data to model network data and model network data to model network data. After applying Benjamini-Hochberg correction, we highlight the non significant p-values ($p>0.05$) indicating no statistical difference between a molecular network and a particular network model.

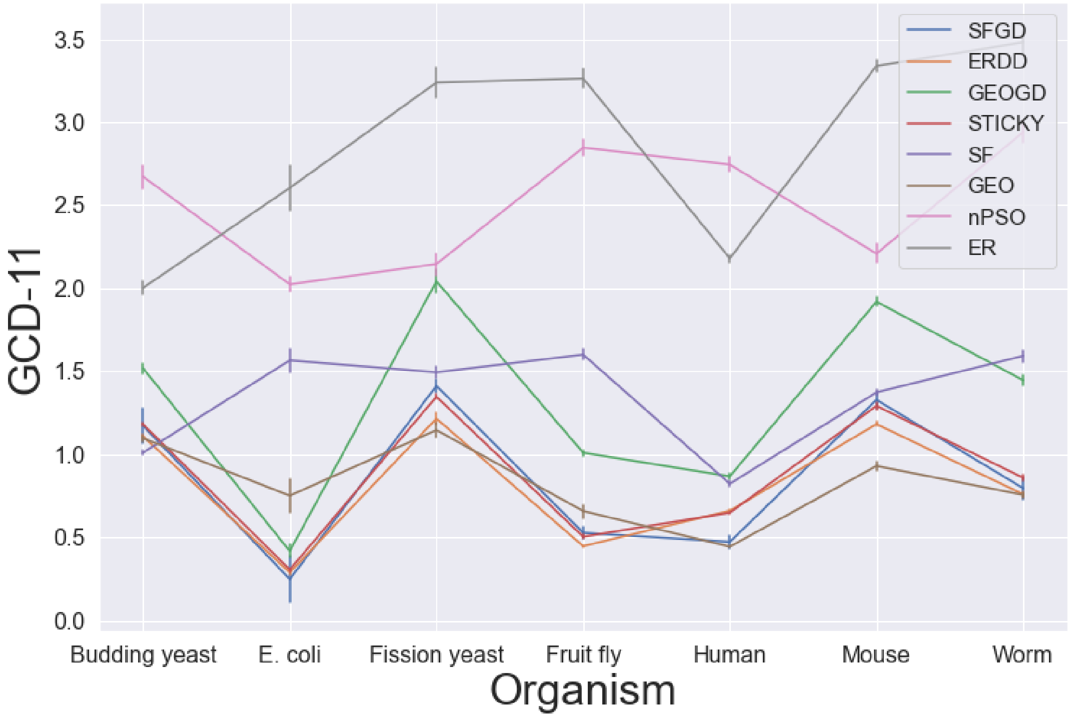

Supplementary Figure 32: **Fit of network models for the PPI networks.** Each line shows the fitting of a network model for the different PPI networks (x-axis). The error-bars indicate the standard deviations of the pairwise GCD-11 distances (y-axis) between the PPI networks and 15 randomly generated networks corresponding to each network model.

Organism	ER	ERDD	GEO	GEOGD	nPSO	SF	SFGD	STICKY
**Budding yeast**	9.63E-07	9.63E-07	9.63E-07	9.63E-07	9.63E-07	9.63E-07	9.63E-07	9.63E-07
**E. coli**	1.10E-06	1.10E-06	1.10E-06	1.10E-06	1.10E-06	1.10E-06	1.10E-06	1.10E-06
**Fission yeast**	9.63E-07	9.63E-07	9.63E-07	9.63E-07	9.63E-07	9.63E-07	9.63E-07	9.63E-07
**Fruit fly**	9.63E-07	9.63E-07	9.63E-07	9.63E-07	9.63E-07	9.63E-07	9.63E-07	9.63E-07
**Human**	9.63E-07	9.63E-07	9.63E-07	9.63E-07	9.63E-07	9.63E-07	9.63E-07	9.63E-07
**House mouse**	9.63E-07	9.63E-07	9.63E-07	9.63E-07	9.63E-07	9.63E-07	9.63E-07	9.63E-07
**Roundworm**	9.63E-07	9.63E-07	9.63E-07	9.63E-07	9.63E-07	9.63E-07	9.63E-07	9.63E-07

Supplementary Table 8: **Mann-Whitney-U test p-values for model network fitting of PPI networks.** For each random model, we perform a MWU test between two distance distributions to evaluate if there is any statistical difference: GCD-11 between the real data to model network data and model network data to model network data. After applying Benjamini-Hochberg correction, all the PPI molecular networks are statistically different than the model networks at a 0.05 level of statistical significance.

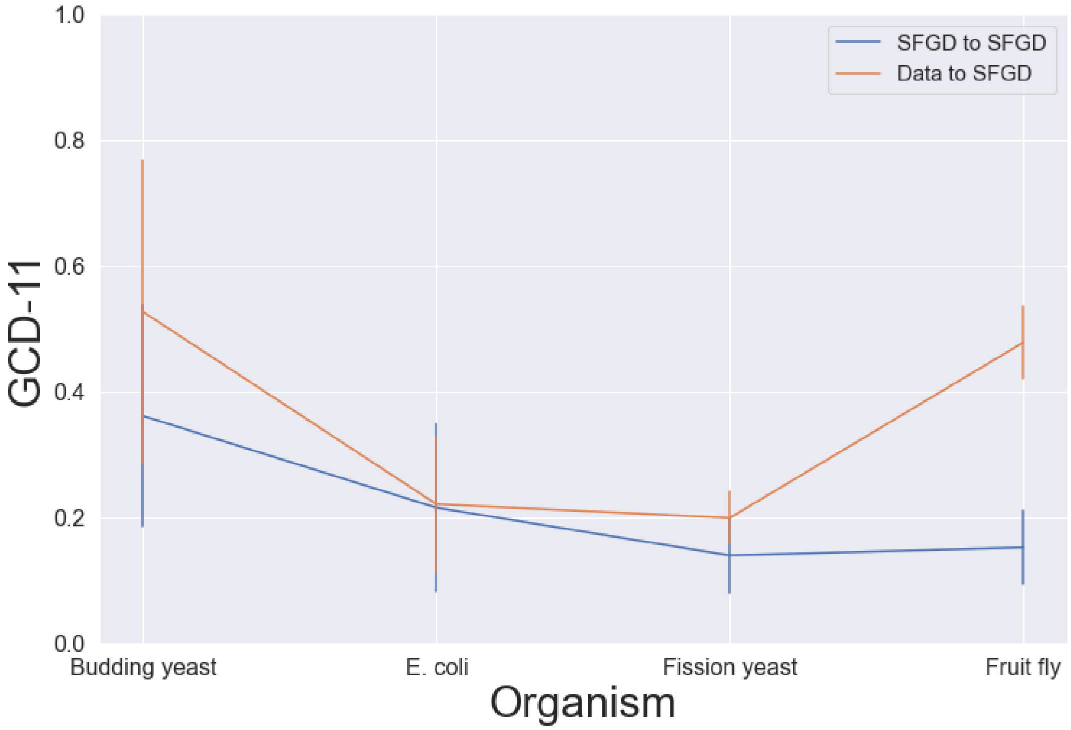

Supplementary Figure 33: **Fit of the SFGD model for the GI networks.** For the four GI networks (x-axis), we show the distribution of model to model (SFGD) GCD-11 distances (blue line) and distribution of real to model GCD-11 distances (orange line). The orange line and error-bars represent the averages and standard deviations of the pairwise GCD-11 distances (y-axis) between the GI networks and 15 randomly generated networks of the same size as the GI networks with scale-free and gene duplication properties. The orange line and error-bars in the blue line represent the same statistics, but between the randomly generated networks.

3.6 Gene paralog results

We observe that triangle based GraCoals, such as GraCoal$_2$, capture the functional organisation of the GI networks of *E. coli*, fission yeast and the budding yeast GI networks well. On the contrary, riangle-based GraCoals, perform poorly on the GI network of fruit-fly (see Section 3.2). This results correlates with our model fitting analysis, where we observe that the former species are fitted by the SF-GD model (see Section 3.3). This leads us to the hypothesis that GraCoal$_2$ (and others) capture GO-BPs with many paralogs. In this section, we aim to confirm this hypothesis. To do so, we first show that the enrichments for *E. coli*, fission yeast and budding yeast contain a lot of paralogs (section 3.6.1). We then explain why GraCoals based on triangle topology are able to capture these paralogous genes. That is, when two genes are paralogous they are form statistically significantly more triangles than if they were non-paralogous (section **??**).

3.6.1 Genes enriched in *E. coli*, Fission yeast and Budding yeast GI networks contain more paralogs than those enriched in the Fruit fly GI network

Below, in Supplementary Table 9, we report the total number of genes that have at least one GO-BP enriched in the neighborhood (i.e., "Enriched genes") and the percentages of genes that are enriched and are paralogs (i.e., "Paralogs"). For the Budding yeast, *E. coli* and the Fission yeast GI networks, we observe that GraCoals that correspond to triangle topology such as GraCoal$_2$ and GraCoal$_7$ tend to be the ones that contain the most paralogs enriched. Conversely, for the Fruit fly GI network, the GraCoals that correspond to triangle topology do not outperform the other GraCoals (except for GraCoal$_8$, and they all achieve less than 10.27% of paralogs enriched.

	**Budding yeast**		***E. coli***		**Fission yeast**		**Fruit fly**	
	Enriched Genes	Paralogs(%)	Enriched Genes	Paralogs(%)	Enriched Genes	Paralogs(%)	Enriched Genes	Paralogs(%)
$\widetilde{A}_{G_0}$	1,189	10.09	1,234	23.26	567	17.64	2,061	7.13
$\widetilde{A}_{G_1}$	1,476	11.04	1,191	26.87	445	16.85	2,121	6.51
$\widetilde{A}_{G_2}$	2,640	21.74	1,258	25.51	709	26.23	1,066	8.92
$\widetilde{A}_{G_3}$	2,551	18.35	1,188	25.67	752	19.28	2,211	7.92
$\widetilde{A}_{G_4}$	1,017	6.19	978	26.48	222	4.50	1,879	10.27
$\widetilde{A}_{G_5}$	1,759	16.09	1,223	25.92	549	20.22	1,036	7.24
$\widetilde{A}_{G_6}$	2,454	17.36	1,174	26.83	833	19.93	1,923	7.28
$\widetilde{A}_{G_7}$	2,509	20.65	1,220	25.49	708	22.46	944	7.31
$\widetilde{A}_{G_8}$	2,036	14.00	835	14.73	406	16.26	421	10.22

Supplementary Table 9: **Statistics for genes enriched and paralogs enriched.** For each of the four GI networks (Budding yeast, *E. coli*, Fission yeast and Fruit fly), we report the number of genes enriched when using SAFE with GraCoal embeddings (i.e., genes that have at least one annotation enriched in their neighborhood) and the percentages of genes enriched that are paralogs.

3.6.2 Paralogous genes touch tend to form triangles in the network

After showing that GraCoals based on triangles capture many paralogs, we assess if these paralogs are involved in many triangles in the networks. To do this, we compute the number of triangles that the pairs of paralogs touch simultaneously in the networks and the number of triangles that pairs of non-paralogs touch in the network. Next, we perform a one-sided MWU test between the distribution of triangle counts for pairs of paralogs and the distribution of triangle counts for the non-paralogous pairs. We observe that for the four GI networks (Supplementary Figure 34), paralog pairs of genes participate in statistically more triangles in the network than the non-paralog pairs of genes (p-values < 0.05).

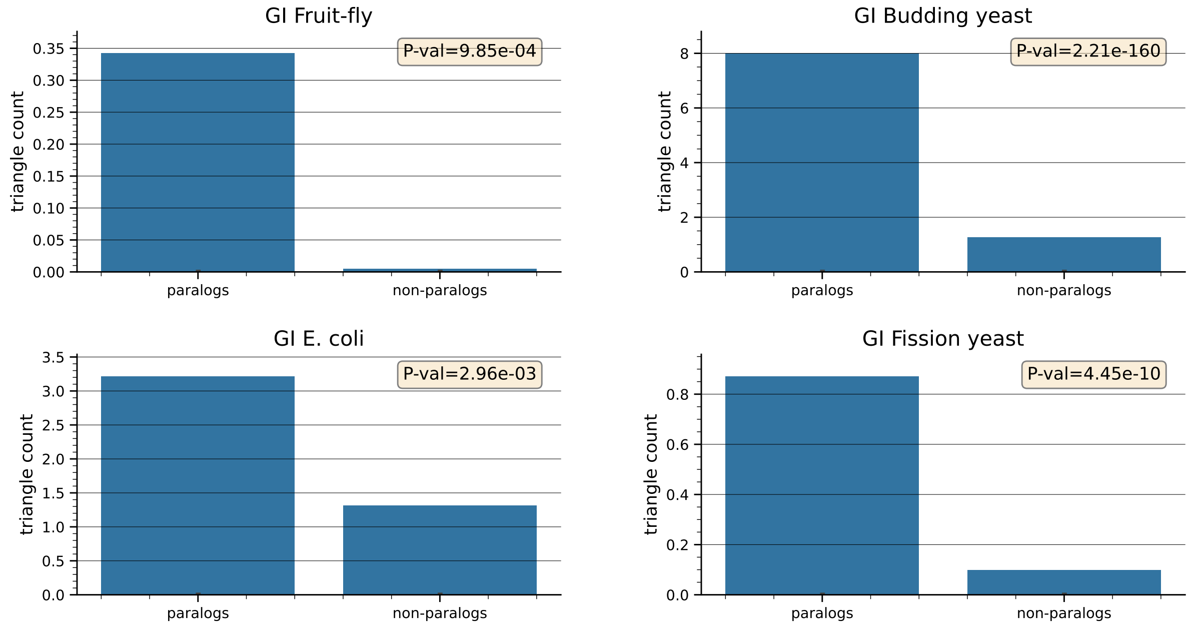

Supplementary Figure 34: **Triangle count distribution for pairs of paralogs and non-paralogs genes.** For each of our GI molecular networks, we show the triangle count distribution for all pairs of paralogs in the network (i.e., blue) and the triangle count distribution for pairs of non-paralogs in the network (i.e., orange). Pairs of paralogs are involved in statistically significantly more triangles than pairs of non-paralogs (MWU p-values ≤ 0.05).

3.7 GraCoal embeddings uncover unique biological functions in GI molecular networks

After having shown that GraCoal embeddings work best for the GI molecular networks when using SAFE, we then focus on identifying what characterizes each particular GraCoal (i.e., $\widetilde{A}_{G_0}$-$\widetilde{A}_{G_8}$) from a biological perspective. To this end, we explore in more detail the functional information uncovered by the GraCoal embeddings when used in SAFE. We do this at the annotation level (i.e., identifying particular GO-BPs that are characteristic of each GraCoal) and at the functional domain level (i.e., identifying particular functional domains that are characteristic of each GraCoal). At the annotation level, we identify the enriched annotations that are unique for each GraCoal (i.e., annotations enriched in a particular GraCoal that are not enriched in any of the other GraCoals). Finally, we rank the uniquely enriched annotations according to their size, defined as the total number of neighborhoods they are enriched in, as a measure of how well they are captured by each particular GraCoal. We report, in Supplementary Tables 10-13 for each particular GraCoal, the total number of uniquely enriched annotations (column 2) and the names of the top 10 largest uniquely enriched annotations (column 3) according to their size in terms of enriched neighborhoods (column 3).

$\widetilde{A}_{G_i}$	**Total annotations**	**EN**	**Annotation**
$\widetilde{A}_{G_0}$	17	193.0	protein localization to mitochondrion
		193.0	establishment of protein localization to mitochondrion
		192.0	mitochondrial transport
		118.0	ribosome disassembly
		110.0	protein insertion into membrane
		95.0	RNA methylation
		88.0	protein insertion into mitochondrial membrane
		85.0	establishment of protein localization to mitochondrial membrane
		82.0	regulation of DNA double-strand break processing
		72.0	tRNA methylation
$\widetilde{A}_{G_1}$	25	176.0	nuclear pore localization
		174.0	tRNA gene clustering
		161.0	positive regulation of attachment of spindle microtubules to kinetochore
		112.0	attachment of spindle microtubules to kinetochore involved in meiotic chromosome segregation
		112.0	monopolar spindle attachment to meiosis I kinetochore
		103.0	DNA unwinding involved in DNA replication
		91.0	positive regulation of chromosome segregation
		72.0	positive regulation of DNA-templated transcription, initiation
		70.0	U1 snRNA 3'-end processing
		66.0	DNA duplex unwinding
$\widetilde{A}_{G_2}$	46	270.0	double-strand break repair via nonhomologous end joining
		232.0	positive regulation of DNA metabolic process
		231.0	regulation of reproductive process
		195.0	endonucleolytic cleavage in ITS1 upstream of 5.8S rRNA from tricistronic rRNA transcript
		194.0	regulation of nuclease activity
		190.0	regulation of deoxyribonuclease activity
		173.0	regulation of endodeoxyribonuclease activity
		166.0	regulation of transcription by RNA polymerase I
		161.0	positive regulation of deoxyribonuclease activity
		161.0	positive regulation of nuclease activity
$\widetilde{A}_{G_3}$	21	182.0	retrograde vesicle-mediated transport, Golgi to endoplasmic reticulum
		165.0	protein methylation
		165.0	protein alkylation
		157.0	peptidyl-lysine methylation
		154.0	replication fork arrest
		146.0	Golgi organization
		140.0	mitotic DNA damage checkpoint signaling
		128.0	mitotic intra-S DNA damage checkpoint signaling
		124.0	histone H3-K79 methylation
		118.0	vesicle fusion with Golgi apparatus

Supplementary Table 10: **Summary of uniquely enriched GO-BPs for Gracoal embeddings on the budding yeast GI network, Part 1.** For the budding yeast GI network, we report, for GraCoals based on all graphlet adjacencies for up to four node graphlets, i.e., $\widetilde{A}_{G_0}$ to $\widetilde{A}_{G_8}$ (column 1), the number of uniquely enriched GO-BPs (column 2) and the names of the top ten largest GO-BPs (column 4), i.e., ranking them in descending order according to the number of neighborhoods in which they are enriched (column 4).

$\widetilde{A}_{G_i}$	**Total annotations**	**EN**	**Annotation**
$\widetilde{A}_{G_4}$	7	93.0	positive regulation of cell cycle process
		90.0	positive regulation of cell cycle
		13.0	phosphatidylcholine biosynthetic process
		13.0	phosphatidylcholine metabolic process
$\widetilde{A}_{G_5}$	35	200.0	cellular response to biotic stimulus
		200.0	response to cell cycle checkpoint signaling
		196.0	response to biotic stimulus
		192.0	cellular response to endogenous stimulus
		192.0	response to endogenous stimulus
		74.0	vacuole organization
		74.0	regulation of signal transduction
		74.0	regulation of signaling
		70.0	regulation of intracellular signal transduction
		70.0	vacuole fusion, non-autophagic
$\widetilde{A}_{G_6}$	1	101	ribosomal small unit export from nucleus
$\widetilde{A}_{G_7}$	13	152.0	regulation of cellular response to stress
		131.0	regulation of response to stress
		95.0	reproduction
		87.0	regulation of response to endoplasmic reticulum stress
		85.0	protein targeting to membrane
		83.0	regulation of endoplasmic reticulum unfolded protein response
		65.0	response to unfolded protein
		28.0	cellular response to abiotic stimulus
		28.0	cellular response to environmental stimulus
		14.0	cellular response to osmotic stress
$\widetilde{A}_{G_8}$	34	239.0	transcription by RNA polymerase II
		159.0	cellular chemical homeostasis
		151.0	regulation of cellular component organization
		139.0	regulation of microtubule-based process
		126.0	ubiquitin-dependent ERAD pathway
		116.0	autophagy of peroxisome
		114.0	chromosome organization involved in meiotic cell cycle
		111.0	regulation of microtubule cytoskeleton organization
		110.0	sphingolipid metabolic process
		110.0	chemical homeostasis

Supplementary Table 10: **Summary of uniquely enriched GO-BPs for Gracoal embeddings on the budding yeast GI network, Part 2.** For the budding yeast GI network, we report, for GraCoals based on all graphlet adjacencies for up to four node graphlets, i.e., $\widetilde{A}_{G_0}$ to $\widetilde{A}_{G_8}$ (column 1), the number of uniquely enriched GO-BPs (column 2) and the names of the top ten largest GO-BPs (column 4), i.e., ranking them in descending order according to the number of neighborhoods in which they are enriched (column 4).

$\widetilde{A}_{G_i}$	Total annotations	EN	Annotation
$\widetilde{A}_{G_0}$	18	142.0	peptide metabolic process
		121.0	rRNA modification
		119.0	ribosomal large subunit assembly
		119.0	pseudouridine synthesis
		118.0	N-terminal protein amino acid modification
		115.0	RNA methylation
		114.0	metabolic process
		111.0	organic substance metabolic process
		102.0	tRNA methylation
		100.0	peptide catabolic process
$\widetilde{A}_{G_2}$	10	141.0	negative regulation of DNA-templated DNA replication
		138.0	negative regulation of DNA replication
		138.0	negative regulation of DNA metabolic process
		129.0	cell communication
		110.0	regulation of DNA replication
		101.0	response to extracellular stimulus
		47.0	isopentenyl diphosphate metabolic process
		47.0	glyceraldehyde-3-phosphate metabolic process
		47.0	isopentenyl diphosphate biosynthetic process
		47.0	isopentenyl diphosphate biosynthetic process, methylerythritol 4-phosphate pathway
$\widetilde{A}_{G_3}$	7	185.0	DNA topological change
		102.0	viral process
		93.0	regulation of DNA recombination
		93.0	translesion synthesis
		49.0	bile acid and bile salt transport
		32.0	organic anion transport
$\widetilde{A}_{G_4}$	19	159.0	enterobacterial common antigen metabolic process
		159.0	enterobacterial common antigen biosynthetic process
		150.0	glycerophospholipid biosynthetic process
		150.0	glycerolipid biosynthetic process
		136.0	glucan biosynthetic process
		131.0	glycogen biosynthetic process
		112.0	glycerolipid metabolic process
		112.0	glycerophospholipid metabolic process
		96.0	lipid modification
		80.0	glycerol-3-phosphate catabolic process

Supplementary Table 11: **Summary of uniquely enriched GO-BPs for Gracoal embeddings on the *E. coli* GI network, Part 1.** For the *E. coli* GI network, we report, for GraCoals based on all graphlet adjacencies for up to four node graphlets, i.e., $\widetilde{A}_{G_0}$ to $\widetilde{A}_{G_8}$ (column 1), the number of uniquely enriched GO-BPs (column 2) and the names of the top ten largest GO-BPs (column 4), i.e., ranking them in descending order according to the number of neighborhoods in which they are enriched (column 4).

$\widetilde{A}_{G_i}$	**Total annotations**	**EN**	**Annotation**
$\widetilde{A}_{G_5}$	2	73.0	tRNA modification
		31.0	proteolysis involved in cellular protein catabolic process
$\widetilde{A}_{G_7}$	3	15.0	copper ion transport
		15.0	copper ion transmembrane transport
		15.0	copper ion export
$\widetilde{A}_{G_8}$	6	52.0	dipeptide transport
		49.0	dipeptide transmembrane transport
		38.0	organophosphate ester transport
		37.0	heme transport
		37.0	aerobic electron transport chain
		29.0	glycerol-3-phosphate transmembrane transport

Supplementary Table 11: **Summary of uniquely enriched GO-BPs for Gracoal embeddings on the *E. coli* GI network, Part 2.** For the *E. coli* GI network, we report, for GraCoals based on all graphlet adjacencies for up to four node graphlets, i.e., $\widetilde{A}_{G_0}$ to $\widetilde{A}_{G_8}$ (column 1), the number of uniquely enriched GO-BPs (column 2) and the names of the top ten largest GO-BPs (column 4), i.e., ranking them in descending order according to the number of neighborhoods in which they are enriched (column 4).

$\widetilde{A}_{G_i}$	**Total annotations**	**EN**	**Annotation**
$\widetilde{A}_{G_0}$	5	167.0	regulation of biological process
		167.0	regulation of cell cycle switching, mitotic to meiotic cell cycle
		120.0	negative regulation of conjugation with cellular fusion
		86.0	regulation of cell cycle G1/S phase transition
		86.0	regulation of G1/S transition of mitotic cell cycle
$\widetilde{A}_{G_2}$	24	95.0	regulation of Ras protein signal transduction
		95.0	regulation of small GTPase mediated signal transduction
		90.0	regulation of cell wall macromolecule metabolic process
		90.0	regulation of polysaccharide biosynthetic process
		90.0	regulation of glucan biosynthetic process
		90.0	regulation of polysaccharide metabolic process
		88.0	regulation of septation initiation signaling
		87.0	regulation of (1->3)-beta-D-glucan biosynthetic process
		87.0	regulation of beta-glucan biosynthetic process
		87.0	regulation of cell wall (1->3)-beta-D-glucan biosynthetic process
$\widetilde{A}_{G_3}$	3	91.0	cellular component assembly
		67.0	protein-DNA complex subunit organization
		40.0	protein-DNA complex assembly
$\widetilde{A}_{G_4}$	7	15.0	RNA splicing, via transesterification reactions
		15.0	RNA splicing
		15.0	mRNA processing
		15.0	mRNA cis splicing, via spliceosome
		15.0	RNA splicing, via transesterification reactions with bulged adenosine as nucleophile
		15.0	mRNA splicing, via spliceosome
		13.0	RNA processing

Supplementary Table 12: **Summary of uniquely enriched GO-BPs for Gracoal embeddings on the fission yeast GI network, Part 1.** For the fission yeast GI network, we report, for GraCoals based on all graphlet adjacencies for up to four node graphlets, i.e., $\widetilde{A}_{G_0}$ to $\widetilde{A}_{G_8}$ (column 1), the number of uniquely enriched GO-BPs (column 2) and the names of the top ten largest GO-BPs (column 4), i.e., ranking them in descending order according to the number of neighborhoods in which they are enriched (column 4).

$\widetilde{A}_{G_i}$	**Total annotations**	**EN**	**Annotation**
$\widetilde{A}_{G_5}$	17	237.0	ubiquitin-dependent protein catabolic process
		237.0	modification-dependent protein catabolic process
		236.0	proteolysis involved in cellular protein catabolic process
		235.0	modification-dependent macromolecule catabolic process
		227.0	protein metabolic process
		211.0	macromolecule catabolic process
		180.0	regulation of primary metabolic process
		169.0	regulation of macromolecule metabolic process
		162.0	regulation of metabolic process
		13.0	regulation of nucleic acid-templated transcription
$\widetilde{A}_{G_6}$	27	226.0	cell cycle DNA replication maintenance of fidelity
		226.0	mitotic recombination-dependent replication fork processing
		226.0	mitotic DNA replication maintenance of fidelity
		188.0	regulation of cytokinetic process
		152.0	UV-damage excision repair
		152.0	response to radiation
		152.0	cellular response to light stimulus
		152.0	cellular response to UV
		152.0	cellular response to radiation
		152.0	response to light stimulus
$\widetilde{A}_{G_7}$	10	133.0	regulation of mitotic cytokinetic process
		103.0	cellular glucan metabolic process
		103.0	glucan metabolic process
		103.0	glucan biosynthetic process
		99.0	DNA biosynthetic process
		85.0	gene conversion
		83.0	telomere organization
		83.0	telomere maintenance
		77.0	protein localization to cell periphery
		48.0	nucleotide-excision repair
$\widetilde{A}_{G_8}$	40	90.0	regulation of reproductive process
		79.0	regulation of cellular protein catabolic process
		79.0	positive regulation of cellular protein catabolic process
		79.0	regulation of protein catabolic process
		79.0	positive regulation of protein catabolic process
		73.0	positive regulation of mitotic cell cycle phase transition
		72.0	positive regulation of proteasomal ubiquitin-dependent protein catabolic process
		72.0	regulation of proteolysis involved in cellular protein catabolic process
		72.0	regulation of proteasomal ubiquitin-dependent protein catabolic process
		72.0	positive regulation of ubiquitin-dependent protein catabolic process

Supplementary Table 12: **Summary of uniquely enriched GO-BPs for Gracoal embeddings on the fission yeast GI network, Part 2.** For the fission yeast GI network, we report, for GraCoals based on all graphlet adjacencies for up to four node graphlets, i.e., $\widetilde{A}_{G_0}$ to $\widetilde{A}_{G_8}$ (column 1), the number of uniquely enriched GO-BPs (column 2) and the names of the top ten largest GO-BPs (column 4), i.e., ranking them in descending order according to the number of neighborhoods in which they are enriched (column 4).

$\widetilde{A}_{G_i}$	**Total annotations**	**EN**	**Annotation**
$\widetilde{A}_{G_0}$	67	555.0	cellular component organization
		554.0	cellular component organization or biogenesis
		403.0	cell division
		303.0	organelle localization
		276.0	response to radiation
		247.0	detection of stimulus involved in sensory perception
		241.0	adult behavior
		234.0	regulation of membrane potential
		222.0	calcium ion transport
		218.0	positive regulation of filopodium assembly
$\widetilde{A}_{G_1}$	59	492.0	response to stimulus
		347.0	system process
		315.0	behavior
		311.0	animal organ formation
		311.0	heart formation
		245.0	wing disc anterior/posterior pattern formation
		236.0	sensory perception of smell
		230.0	wing disc development
		229.0	neuroblast fate determination
		223.0	sensory perception of chemical stimulus
$\widetilde{A}_{G_2}$	10	162.0	morphogenesis of a polarized epithelium
		140.0	cellular response to stimulus
		85.0	establishment of proximal/distal cell polarity
		85.0	imaginal disc-derived wing hair site selection
		72.0	asymmetric protein localization involved in cell fate determination
		60.0	cell-cell junction organization
		45.0	positive regulation of protein kinase B signaling
$\widetilde{A}_{G_3}$	40	390.0	regulation of trans-synaptic signaling
		390.0	modulation of chemical synaptic transmission
		229.0	regulation of actin filament bundle assembly
		192.0	organic substance metabolic process
		188.0	gonad development
		174.0	regulation of circadian sleep/wake cycle, sleep
		173.0	larval midgut cell programmed cell death
		173.0	regulation of circadian sleep/wake cycle
		166.0	synapse assembly
		165.0	oenocyte differentiation

Supplementary Table 13: **Summary of uniquely enriched GO-BPs for Gracoal embeddings on the fruit fly GI network, Part 1.** For the fruit fly GI network, we report, for GraCoals based on all graphlet adjacencies for up to four node graphlets, i.e., $\widetilde{A}_{G_0}$ to $\widetilde{A}_{G_8}$ (column 1), the number of uniquely enriched GO-BPs (column 2) and the names of the top ten largest GO-BPs (column 4), i.e., ranking them in descending order according to the number of neighborhoods in which they are enriched (column 4).

$\widetilde{A}_{G_i}$	**Total annotations**	**EN**	**Annotation**
$\widetilde{A}_{G_4}$	30	363.0	cell fate determination
		263.0	heart development
		245.0	pericardial nephrocyte differentiation
		233.0	response to mechanical stimulus
		193.0	regulation of multi-organism process
		188.0	neuronal stem cell population maintenance
		168.0	defense response
		168.0	response to biotic stimulus
		168.0	response to external biotic stimulus
		164.0	defense response to other organism
$\widetilde{A}_{G_5}$	47	247.0	cell cycle comprising mitosis without cytokinesis
		247.0	syncytial blastoderm mitotic cell cycle
		233.0	mitotic cell cycle, embryonic
		232.0	anterior/posterior axis specification
		193.0	regulation of biological quality
		191.0	DNA conformation change
		190.0	regulation of mitotic cell cycle phase transition
		189.0	regulation of cell cycle phase transition
		177.0	meiotic chromosome segregation
		167.0	organelle fission
$\widetilde{A}_{G_6}$	40	263.0	regulation of chromatin organization
		247.0	histone modification
		247.0	covalent chromatin modification
		208.0	appendage segmentation
		208.0	imaginal disc-derived leg segmentation
		195.0	peptidyl-lysine modification
		152.0	protein metabolic process
		151.0	embryonic anterior midgut (ectodermal) morphogenesis
		146.0	multi-organism cellular process
		146.0	multi-organism metabolic process

Supplementary Table 13: **Summary of uniquely enriched GO-BPs for Gracoal embeddings on the fruit fly GI network, Part 2.** For the fruit fly GI network, we report, for GraCoals based on all graphlet adjacencies for up to four node graphlets, i.e., $\widetilde{A}_{G_0}$ to $\widetilde{A}_{G_8}$ (column 1), the number of uniquely enriched GO-BPs (column 2) and the names of the top ten largest GO-BPs (column 4), i.e., ranking them in descending order according to the number of neighborhoods in which they are enriched (column 4).

$\widetilde{A}_{G_i}$	**Total annotations**	**EN**	**Annotation**
$\widetilde{A}_{G_7}$	15	223.0	regulation of cell projection organization
		223.0	regulation of plasma membrane bounded cell projection organization
		151.0	cellular component assembly
		97.0	Rho protein signal transduction
		92.0	determination of adult lifespan
		85.0	imaginal disc-derived appendage development
		79.0	appendage development
		72.0	positive regulation of transmembrane receptor protein serine/threonine kinase signaling pathway
		72.0	negative regulation of cell cycle G1/S phase transition
		72.0	negative regulation of G1/S transition of mitotic cell cycle
$\widetilde{A}_{G_8}$	5	36.0	positive regulation of immune system process
		27.0	regulation of immune response
		24.0	negative regulation of cell cycle phase transition
		24.0	negative regulation of mitotic cell cycle phase transition

Supplementary Table 13: **Summary of uniquely enriched GO-BPs for Gracoal embeddings on the fruit fly GI network, Part 3.** For the fruit fly GI network, we report, for GraCoals based on all graphlet adjacencies for up to four node graphlets, i.e., $\widetilde{A}_{G_0}$ to $\widetilde{A}_{G_8}$ (column 1), the number of uniquely enriched GO-BPs (column 2) and the names of the top ten largest GO-BPs (column 4), i.e., ranking them in descending order according to the number of neighborhoods in which they are enriched (column 4).

3.8 GraCoal embeddings uncover unique functional domains in GI networks

Lastly, we observe that unique domains uncovered by the triangle topology tend to have large paralog ratios. For instance, the most characteristic functional domain of $GraCoal_8$, with JI=0.0, has the largest paralog ratio (0.43). Interestingly, the GO-BPs involved are related to chitin biosynthesis, and the genes that are annotated by these biological processes form a protein complex called the exomer that is assembled in the trans-Golgi network, and is required for the delivery of a distinct set of proteins to the plasma membrane. Gene duplication events lead to the formation of the exomer [19]. We find that $GraCoal_2$ also captures this gene duplication relationship quite well, as it has a functional domain also with the largest paralog ratio (0.43) and a low similarity score (JI=0.12). This domain is composed of GO-BPs such as 'export from cell', 'secretion by cell', 'secretion' and 'exocytosis', which are all vesicle traffic related functions. Previous studies suggest there is a link between genome duplication and the expansion and diversification of the vesicle traffic pathway [20]. They prove that gene duplication allowed the formation of paralogous modules which are responsible for complex and diverse functions such as the vesicle traffic pathway. They emphasize that the many properties of paralogs such as that they can be differentially expressed or regulated, have different interaction partners or other distinct properties, represent a selective advantage when acting in union as modules. For the vesicle traffic pathway, this potentially increases its versatility [20]. Finally, we measure the overlap between the genes enriched in this particular functional domain and the genes that are reported [20] as part of this module of paralogs (JI=0.21) (i.e., 12 out of 14 genes enriched in the functional domains are reported).

$\widetilde{A}_{G_i}$	Num functional domains	Mean paralog ratio	Domain paralog ratio	Domain max JI	Domain description
$\widetilde{A}_{G_0}$	10	0.16 (std=0.08)	0.32	0.00	cytokinesis, cytoskeleton, septin, organization, histone
			0.15	0.07	regulation, attachment, spindle, microtubules, kinetochore
			0.07	0.13	metabolic, process, glycolipid, liposaccharide
$\widetilde{A}_{G_1}$	15	0.15 (std=0.06)	0.25	0.00	process, amino, acid, biosynthetic, glutamine
			0.11	0.05	histone, methylation, lysine, H3, K4
			0.06	0.13	acetylation, peptidyl, lysine, modification, internal
$\widetilde{A}_{G_2}$	15	0.20 (std=0.09)	0.26	0.00	growth, in, filamentous, conjugation, with
			0.17	0.00	biosynthetic, process, purine, ribonucleotide, nucleotide
			0.43	0.12	secretion, cell, exocytosis, export, by
$\widetilde{A}_{G_3}$	15	0.16 (std=0.07)	0.25	0.00	purine, containing, compound, metabolic, process
			0.16	0.00	electron, transport, chain, aerobic, respiratory
			0.18	0.02	receptor, recycling, protein, import, peroxisome
$\widetilde{A}_{G_4}$	11	0.19 (std=0.12)	0.24	0.00	transition, metal, ion, transport
			0.22	0.00	phosphatidylcholine, process, metabolic, biosynthetic
			0.26	0.08	transport, retrograde, endosome, Golgi, endosomal
$\widetilde{A}_{G_5}$	11	0.18 (std=0.19)	0.15	0.00	transmembrane, transport, hexose, monosaccharide, small
			0.13	0.00	regulation, heterochromatin, assembly, negative, organization
			0.17	0.04	positive, regulation, process, cellular, biological
$\widetilde{A}_{G_6}$	10	0.18 (std=0.19)	0.18	0.00	rRNA, RNA, splicing, transesterification, LSU
			0.12	0.57	rRNA, processing, SSU, RNA, endonucleolytic
			0.14	0.62	catabolic, process, dependent, macromolecule, protein
$\widetilde{A}_{G_7}$	16	0.18 (std=0.19)	0.15	0.00	cellular, response, stimulus, abiotic, osmotic
			0.08	0.08	mRNA, cleavage, polyadenylation, processing, response
			0.26	0.44	rRNA, SSU, processing, endonucleolytic, cleavage
$\widetilde{A}_{G_8}$	10	0.22 (std=0.06)	0.43	0.00	membrane, cell, wall, chitin, process
			0.21	0.00	actin, cytoskeleton, organization, filament, based
			0.21	0.12	response, compound, organonitrogen, ERAD, pathway

Supplementary Table 14: **Summary of most unique functional domains for Gracoal embeddings on the budding yeast GI network.** We report, for each GraCoal embedding used with SAFE based on all graphlet adjacencies for up to four node graphlets, i.e., $\widetilde{A}_{G_0}$ to $\widetilde{A}_{G_8}$ (column 1), the number of functional domains (column 2) the mean paralog ratio (column 3) and the top three most characteristic functional domains (column 6). Lastly, for each functional domain we report its paralog ratio (column 4) and the maximum Jaccard similarity index (JI).

$\widetilde{A}_{G_i}$	**Num functional domains**	**Mean paralog ratio**	**Domain paralog ratio**	**Domain max JI**	**Domain description**
$\widetilde{A}_{G_0}$	12	0.21 (std=0.16)	0.20	0.25	protein, transport, establishment, localization, targeting
			0.22	0.35	process, metabolic, rRNA, tRNA, pseudouridine
			0.24	0.50	localization, membrane, lipoprotein, protein, cellular
$\widetilde{A}_{G_1}$	7	0.23 (std=0.17)	0.18	0.30	metabolic, process, processing, tRNA, rRNA
			0.07	0.50	process, cell, transport, protein, biosynthetic
			0.14	0.57	process, localization, metabolic, biosynthetic, cellular
$\widetilde{A}_{G_2}$	9	0.15 (std=0.13)	0.12	0.10	proteolysis
			0.12	0.35	process, metabolic, biosynthetic, regulation, isopentenyl
			0.33	0.69	localization, transport, protein, cellular, macromolecule
$\widetilde{A}_{G_3}$	8	0.21 (std=0.17)	0.52	0.39	transport, ion, iron, localization, organic
			0.00	0.50	process, biosynthetic, cell, macromolecule, wall
			0.17	0.57	process, biosynthetic, localization, regulation, cellular
$\widetilde{A}_{G_4}$	9	0.17 (std=0.09)	0.05	0.42	localization, process, protein, cell, cytokinesis
			0.14	0.43	process, biosynthetic, metabolic, lipid, cellular
			0.17	0.44	localization, protein, membrane, cellular, within
$\widetilde{A}_{G_5}$	11	0.22 (std=0.16)	0.17	0.22	ubiquinone, process, biosynthetic, metabolic
			0.37	0.40	rRNA, metabolic, process, processing
			0.51	0.44	iron, ion, transport, cell, import
$\widetilde{A}_{G_6}$	10	0.21 (std=0.17)	0.14	0.50	process, localization, metabolic, cellular, lipid
			0.17	0.67	processing, ncRNA, RNA
			0.29	0.70	homeostasis, ion, cellular, metal, copper
$\widetilde{A}_{G_7}$	12	0.19 (std=0.16)	0.00	0.22	ion, copper, transport, detoxification, homeostasis
			0.19	0.40	subunit, assembly, rRNA, ribonucleoprotein, complex
			0.16	0.47	processing, macromolecule, methylation, RNA, modification
$\widetilde{A}_{G_8}$	6	0.18 (std=0.09)	0.12	0.00	transport, glycerol, 3, phosphate, transmembrane
			0.36	0.48	transport, localization, protein, establishment, transmembrane
			0.17	0.50	metabolic, process, compound, cellular, DNA

Supplementary Table 15: **Summary of most unique functional domains for Gracoal embeddings on the *E. coli* GI network.** We report, for each GraCoal embedding used with SAFE based on all graphlet adjacencies for up to four node graphlets, i.e., $\widetilde{A}_{G_0}$ to $\widetilde{A}_{G_8}$ (column 1), the number of functional domains (column 2) the mean paralog ratio (column 3) and the top three most characteristic functional domains (column 6). Lastly, for each functional domain we report its paralog ratio (column 4) and the maximum Jaccard similarity index (JI).

$\widetilde{A}_{G_i}$	Num functional domains	Mean paralog ratio	Domain paralog ratio	Domain max JI	Domain description
$\widetilde{A}_{G_0}$	2	0.12 (std=0.12)	0.00	0.09	DNA, replication, initiation, cell, cycle
			0.24	0.27	regulation, cell, cycle, mitotic, negative
$\widetilde{A}_{G_1}$	3	0.03 (std=0.04)	0.00	0.03	regulation, mitotic, division, septum, assembly
			0.08	0.07	DNA, repair
			0.00	0.50	DNA, replication, independent, chromatin, assembly
$\widetilde{A}_{G_2}$	5	0.12 (std=0.10)	0.00	0.18	homeostasis, ion, cellular, calcium, chemical
			0.06	0.28	heterochromatin, assembly, organization, constitutive, negative
			0.21	0.33	regulation, process, positive, biological, conjugation
$\widetilde{A}_{G_3}$	2	0.08 (std=0.03)	0.10	0.54	DNA, cell, cycle, process, mitotic
			0.05	0.56	assembly, heterochromatin, organization, chromatin, cellular
$\widetilde{A}_{G_4}$	3	0.04 (std=0.05	0.00	0.00	splicing, RNA, mRNA, transesterification, reactions
			0.11	0.05	organization, cellular, component, or, biogenesis
			0.00	0.50	DNA, replication, independent, chromatin, organization
$\widetilde{A}_{G_5}$	5	0.13 (std=0.10)	0.27	0.00	regulation, biosynthetic, process, transcription, templated
			0.23	0.07	regulation, cellular, component, biogenesis, positive
			0.02	0.08	repair, recombination, double, strand, break
$\widetilde{A}_{G_6}$	6	0.22 (std=0.19)	0.42	0.00	negative, regulation, response, stimulus, cell
			0.07	0.00	transport, anion, transmembrane, ion, organic
			0.53	0.15	positive, regulation, cell, cycle, phase
$\widetilde{A}_{G_7}$	5	0.10 (std=0.10)	0.00	0.18	regulation, cytosolic, calcium, ion, concentration
			0.28	0.33	regulation, process, positive, cell, cycle
			0.12	0.36	regulation, assembly, process, organization, actomyosin
$\widetilde{A}_{G_8}$	6	0.15 (std=0.06)	0.13	0.00	chromosome, segregation, attachment, spindle, microtubules
			0.24	0.03	microtubule, organization, transport, based, cytoskeleton
			0.07	0.24	metabolic, process, compound, cellular, DNA

Supplementary Table 16: **Summary of most unique functional domains for Gracoal embeddings on the fission yeast GI network.** We report, for each GraCoal embedding used with SAFE based on all graphlet adjacencies for up to four node graphlets, i.e., $\widetilde{A}_{G_0}$ to $\widetilde{A}_{G_8}$ (column 1), the number of functional domains (column 2) the mean paralog ratio (column 3) and the top three most characteristic functional domains (column 6). Lastly, for each functional domain we report its paralog ratio (column 4) and the maximum Jaccard similarity index (JI).

$\widetilde{A}_{G_i}$	Num functional domains	Mean paralog ratio	Domain paralog ratio	Domain max JI	Domain description
			0.22	0.00	response, regulation, cuticle, stress, chitin
$\widetilde{A}_{G_0}$	15	0.06 (std=0.07)	0.00	0.00	intrinsic, apoptotic, signaling, pathway, in
			0.00	0.05	dosage, compensation
			0.00	0.00	mitotic, negative, regulation, spindle, checkpoint
$\widetilde{A}_{G_1}$	13	0.06 (std=0.05)	0.11	0.10	regulation, TORC1, signaling
			0.05	0.15	cell, establishment, polarity, organization, maintenance
			0.09	0.16	regulation, signaling, pathway, growth, neuromuscular
$\widetilde{A}_{G_2}$	9	0.06 (std=0.04)	0.07	0.18	regulation, cell, establishment, polarity, organization
			0.03	0.32	regulation, negative, process, cell, metabolic
			0.07	0.02	synaptic, response, external, stimulus, cell
$\widetilde{A}_{G_3}$	11	0.05 (std=0.03)	0.04	0.16	cell, regulation, signaling, pathway, in
			0.11	0.20	regulation, process, positive, cell, cellular
			0.08	0.00	muscle, cell, cellular, homeostasis
$\widetilde{A}_{G_4}$	10	0.07 (std=0.06)	0.08	0.00	synaptic, signaling, trans, anterograde, chemical
			0.00	0.00	olfactory, behavior
			0.03	0.03	guidance, neuron, projection, axon
$\widetilde{A}_{G_5}$	9	0.05 (std=0.05)	0.07	0.16	regulation, positive, process, cell, negative
			0.19	0.21	detection, stimulus, phototransduction, external, light
			0.04	0.20	regulation, signaling, insulin, receptor, pathway
$\widetilde{A}_{G_6}$	11	0.06 (std=0.05)	0.16	0.22	regulation, cascade, morphogenesis, stress, cell
			0.00	0.25	RNA, 3', end, processing
			0.14	0.00	cofactor, metabolic, process
$\widetilde{A}_{G_7}$	10	0.07 (std=0.05)	0.04	0.02	regulation, Notch, signaling, pathway
			0.09	0.21	regulation, cell, organization, projection, morphogenesis
			0.04	0.19	regulation, response, mitochondrion, organization, cellular
$\widetilde{A}_{G_8}$	4	0.05 (std=0.02)	0.02	0.22	regulation, process, positive, templated, transcription
			0.07	0.32	regulation, cell, morphogenesis, positive, process

Supplementary Table 17: **Summary of most unique functional domains for Gracoal embeddings on the fruit fly GI network.** We report, for each GraCoal embedding used with SAFE based on all graphlet adjacencies for up to four node graphlets, i.e., $\widetilde{A}_{G_0}$ to $\widetilde{A}_{G_8}$ (column 1), the number of functional domains (column 2) the mean paralog ratio (column 3) and the top three most characteristic functional domains (column 6). Lastly, for each functional domain we report its paralog ratio (column 4) and the maximum Jaccard similarity index (JI).

3.9 Robustness of key results to noise in the annotation data

In the main paper, we focus on GI networks for four different species (fruit-fly, budding yeast, E. coli and fission yeast) and GO-BP annotations. We show, based on the union of the percentage of enriched genes (or the enriched annotations in Suppl. Section 3.4), that GraCoal outperforms GraSpring embedding and Graphlet Spectral embedding in general. Additionally, we showed that triangle-based GraCoal embeddings (GraCoal_2 and GraCoal_7) best capture the functional organisation of the budding yeast, fission yeast and E.coli GI networks, and that GraCoal embeddings void of triangles (GraCoal_0, GraCoal_1, GraCoal_3 and GraCoal_4) tend to best capture the functional organisation of the fruit-fly GI network.

Noise in the network data could impact these two results. Here, we show for our four GI networks and GO-BP annotations, in line with the main focus of our paper, that the results are robust with respect to noise in the network data. To simulate noise in the network data, we shuffle the sets of annotations assigned to the genes, varying the degrees of noise from 0% to 30% of shuffled annotations in increments of 10%.

We present the impact for the various degrees of noise on the union of enriched genes (annotations) for GraCoal, GraSpring and Graphlet Spectral embedding applied on our GI networks using GO-BP annotations in Suppl. Fig. 35. We observe that noise does not impact the relative performance of Gracoal, as it remains to outperform GraSpring and Graphlet Spectral embedding regardless of the mount of noise added.

We present the impact for the various degrees of noise on the percentage of enriched genes (annotations) for each of the different Gracoals, applied on our GI networks using GO-BP annotations in Suppl. Fig. 39. We observe that the ranking of the various GraCoals is stable across the various amounts of noise. So the best performing GraCoal for each species does not change depending on the amount of noise.

We conclude that our claim GraCoal embedding is the best performing embedding network for GI networks and GO-BP annotations holds even for highly noisy datasets. Additionally, regardless of the amount of noise, triangle-based GraCoal embeddings (GraCoal_2 and GraCoal_7) best capture the functional organisation of the budding yeast, fission yeast and E.coli GI networks, and GraCoal embeddings void of triangles

(GraCoal$_0$, GraCoal$_1$, GraCoal$_3$ and GraCoal$_4$) tend to best capture the functional organisation of the fruit-fly GI network.

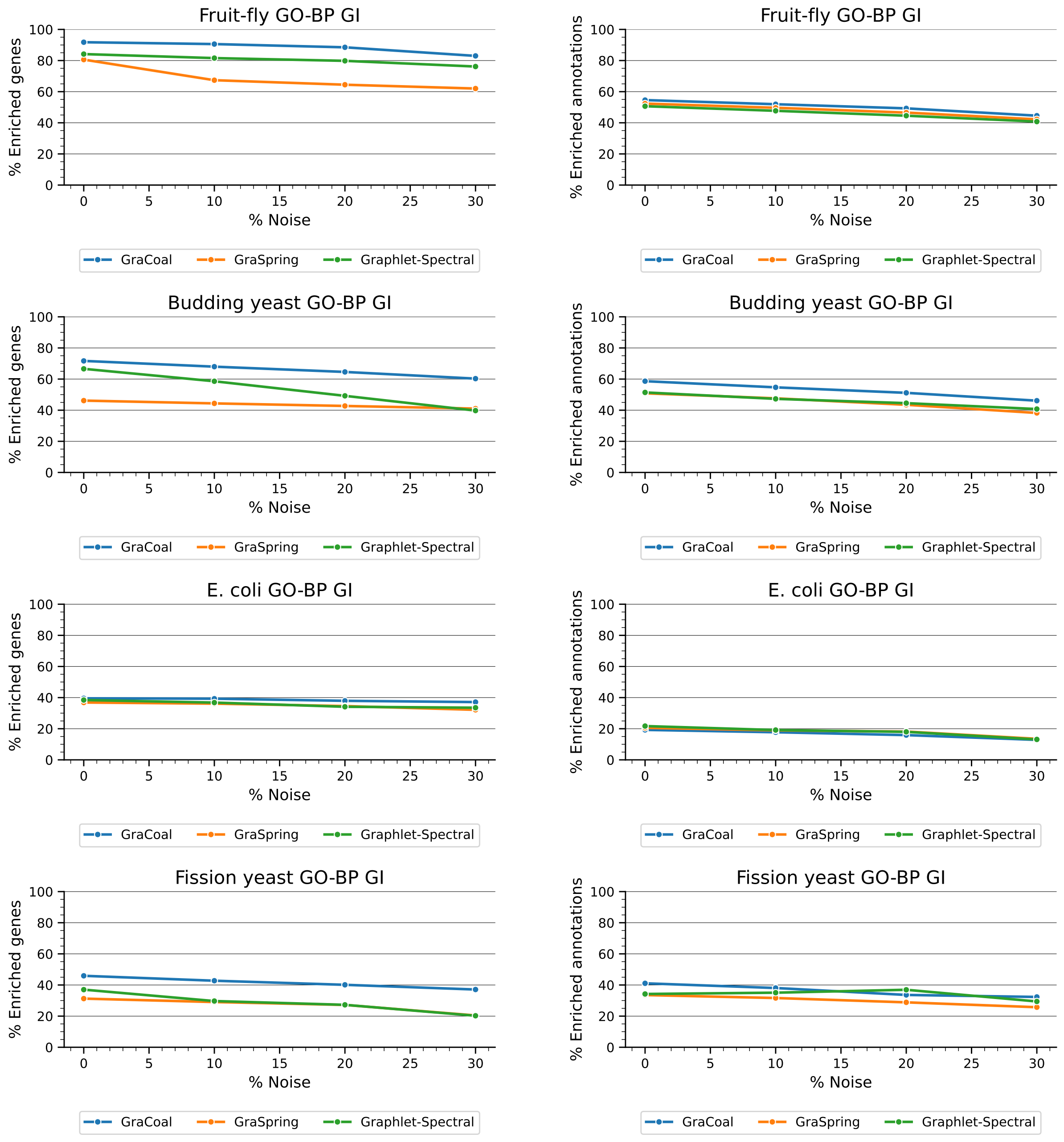

Supplementary Figure 35: **Impact of noise on the best performing embedding algorithm for GI networks.** For each of our GI networks (top to bottom), we report the union of the percentage of enriched genes and annotations (left column, right column) for various degrees of noise (x-axis), for each of the different embedding algorithms (legend).

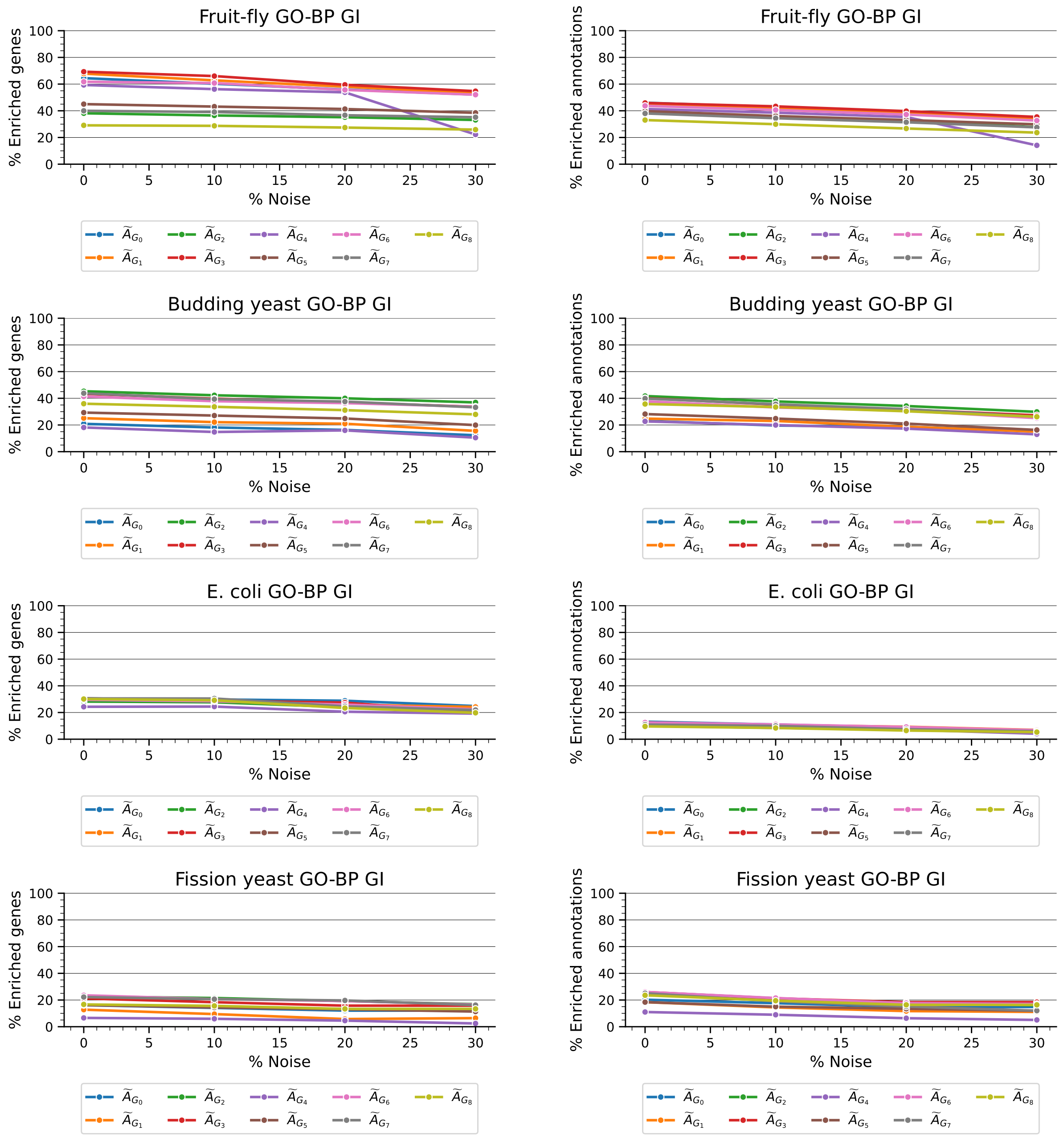

Supplementary Figure 36: **Impact of noise on the best performing GraCoal for GI networks.** For each of our GI networks (top to bottom), we report the the percentage of enriched genes and annotations (left column, right column) for various degrees of noise (x-axis), for each of the different Gracoals (legend).

3.10 Robustness of key results to noise in network data

In the main paper, we focus on GI networks for four different species (fruit-fly, budding yeast, E. coli and fission yeast) and GO-BP annotations. We show, based on the union of the percentage of enriched genes (or the enriched annotations in Suppl. Section 3.4), that GraCoal outperforms GraSpring embedding and Graphlet Spectral embedding in general. Additionally, we show that triangle-based GraCoal embeddings (GraCoal_2 and GraCoal_7) best capture the functional organisation of the budding yeast, fission yeast and E.coli GI networks, and that GraCoal embeddings void of triangles (GraCoal_0, GraCoal_1, GraCoal_3 and GraCoal_4) tend to best capture the functional organisation of the fruit-fly GI network.

Here, we validate that these two results are robust with respect to noise in the network data. To simulate noise, we randomly rewire the edges in the network using the Maslov-Sneppen rewiring algorithm [21]. This algorithm preserves the degree distribution of the network, while randomizing the edges. We vary the amount of noise, in terms of percentage of edges rewired, from 0% to 100% in increments of 25%. By considering noise up to 100% rewired edges with degree preservation, we can quantify how much of the enrichment is coming from the connectivity information of the network and how much is coming from the degree information [22].

We present the impact for the various amounts of noise on the union of enriched genes (annotations) for GraCoal, GraSpring and Graphlet Spectral embedding applied on our GI networks using GO-BP annotations in Suppl. Fig. 37. We observe that noise does not impact the relative performance of GraCoal, as it remains to outperform GraSpring and Graphlet Spectral embedding regardless of the mount of noise added. We also observe that when rewiring 100% of the edges, the percentage of annotations and genes enriched drops considerably for all embeddings but remains different from 0%, indicating that all embeddings capture some degree information, which serves as a source of enrichment.

We present the impact for the various amounts of noise on the percentage of enriched genes (annotations) for each of the different GraCoals, applied on our GI networks using GO-BP annotations in Suppl. Fig. 39. We observe that the ranking of the various GraCoals is stable across the various amounts of noise. So the best performing GraCoal for each species does not change depending on the amount of noise.

We conclude that our claim GraCoal embedding is the best performing embedding network for GI networks and GO-BP annotations holds even for highly noisy datasets. Additionally, regardless of the amount of noise, triangle-based GraCoal embeddings (GraCoal_2 and GraCoal_7) best capture the functional organisation of the budding yeast, fission yeast and E.coli GI networks, and GraCoal embeddings void of triangles (GraCoal_0, GraCoal_1, GraCoal_3 and GraCoal_4) tend to best capture the functional organisation of the fruit-fly GI network.

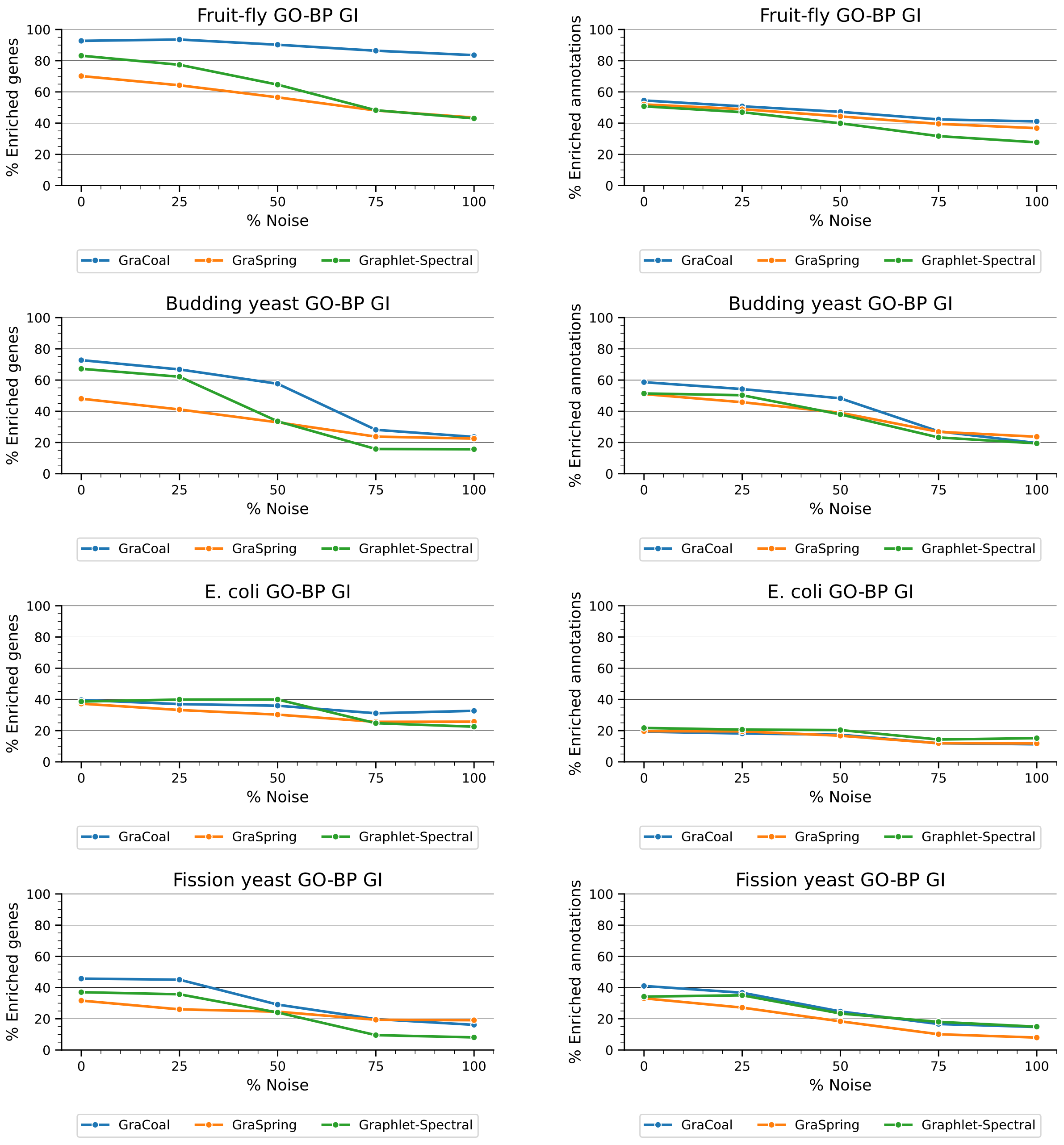

Supplementary Figure 37: **Impact of noise in the network on the best performing embedding algorithm for GI networks.** For each of our GI networks (top to bottom), we report the union of the percentage of enriched genes and annotations (left column, right column) for various degrees of noise (x-axis), for each of the different embedding algorithms (legend).

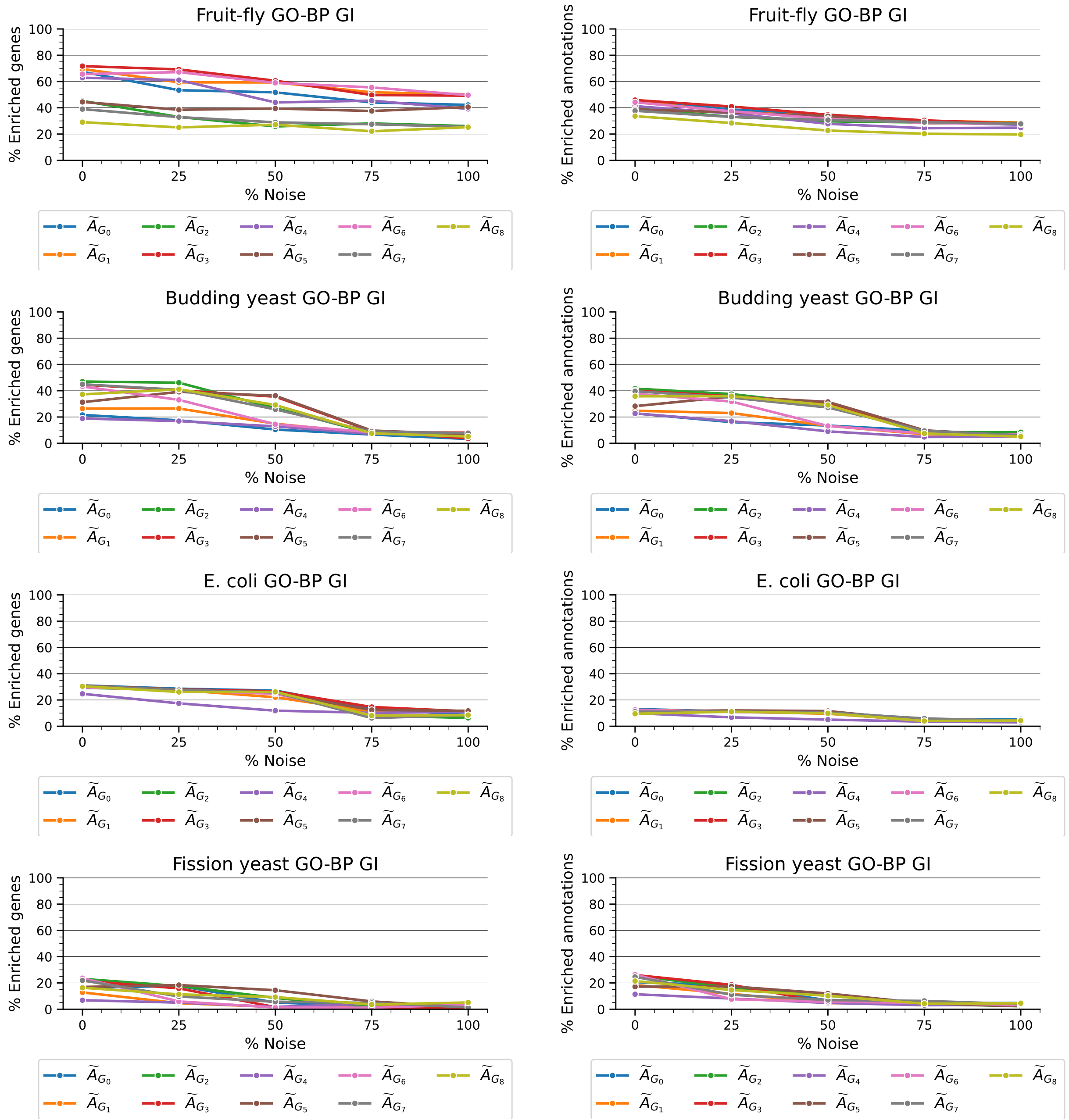

Supplementary Figure 38: **Impact of noise in the network data on the best performing GraCoal for GI networks.** For each of our GI networks (top to bottom), we report the percentage of enriched genes and annotations (left column, right column) for various percentages of rewired edges (x-axis), for each of the different GraCoals (legend).

3.11 Robustness of key results with respect to the average neighbourhood size

In the main paper, we focus on GI networks for four different species (fruit-fly, budding yeast, E. coli and fission yeast) and GO-BP annotations. We show, based on the union of the percentage of enriched genes (or the enriched annotations in the Suppl. Section 3.4), that GraCoal outperforms GraSpring embedding and Graphlet Spectral embedding in general. Additionally, we showed that triangle-based GraCoal embeddings (GraCoal_2 and GraCoal_7) best capture the functional organisation of the budding yeast, fission yeast and E.coli GI networks, and that GraCoal embeddings void of triangles (GraCoal_0, GraCoal_1, GraCoal_3 and GraCoal_4) tend to best capture the functional organisation of the fruit-fly GI network. In further in depth analysis we show that this is due to triangle-based GraCoal embeddings capturing the functional redundancy of paralogous genes.

In our analysis, we set SAFE's neighbourhood parameter α to so that the average neighbourhood size of each node is 50. This, because we found that when varying the average number of neighbours from 10 to 90 in increments of 20, that from 50 onwards the enrichment scores reach a maximum and plateau, for any of the Gracoal embeddings, for of our networks, for any species 3.2. However, given the hierarchical nature of the GO-ontology, considering larger average neighbourhood sizes could influence the functional organisation captured by each of the GraCoals. We already showed, based on the union of the percentage of enriched genes and annotations, that regardless of the chosen average neighbourhood size GraCoal in general outperforms GraSpring embedding and Graphlet spectral embedding in Supp. Section 3.2. Here, we show for the four GI networks and using GO-BP annotations, the main focus of this study, that: (1) varying the average neighbourhood size beyond 50 does not impact the type of best performing Gracoal for any given species and (2) does not significantly impact the functional organisation captured by any GraCoal.

To show that varying the average neighbourhood size does not impact the type of best performing GraCoal, we vary number of average neighbours from 10 to 100 in increments of 10, and from 100 to 1000 in increments of 100, and measure the percentage of enriched genes and annotations for each GraCoal and GI network, using GO-BP annotations in Suppl. Fig 39. We observe that the best performing GraCoal is stable for each network, for both the percentage of enriched genes and annotations, regardless of the average number of neighbours for each node. Additionally, we observe that whereas the percentage of enriched annotations plateaus from 50 onwards, the percentage of enriched genes keeps rising for all networks, for all GraCoals. To illustrate why this happens, we show the enrichment landscape of the 'Protein Prenilation' biological processes for the budding yeast GI network using GraCoal_2 in Suppl. Fig. 40, when setting the average number of neighbours to 50 and 1000, respectively. We observe that when increasing the average neighbourhood size, the genes in whose neighbourhood the 'Protein Prenilation' annotation is enriched increase in terms of frequency and in terms of spread in the embedding space. So, our results suggest that in general, due to the way SAFE defines a gene to be enriched, increasing the average neighbourhood size increases the spread of the genes that can be associated with a given annotation, leading to higher percentages of enriched genes.

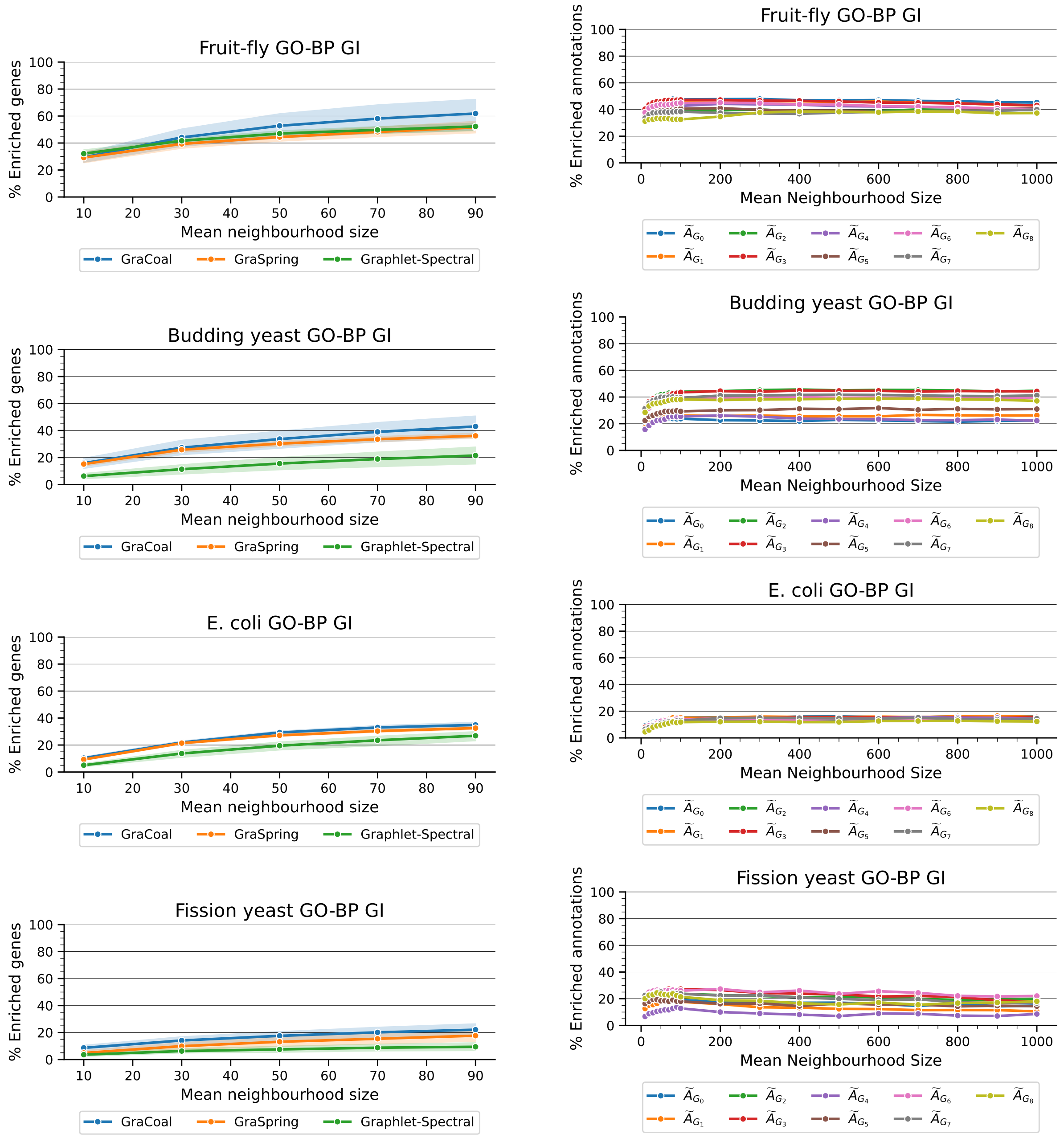

Supplementary Figure 39: **Impact of SAFE's α parameter on the best performing GraCoal algorithm for GI networks.** For each of our GI networks (top to bottom), we report the percentage of enriched genes and annotations (left column, right column) for varying number of average neighbours (x-axis), for each of the different Gracoals (legend).

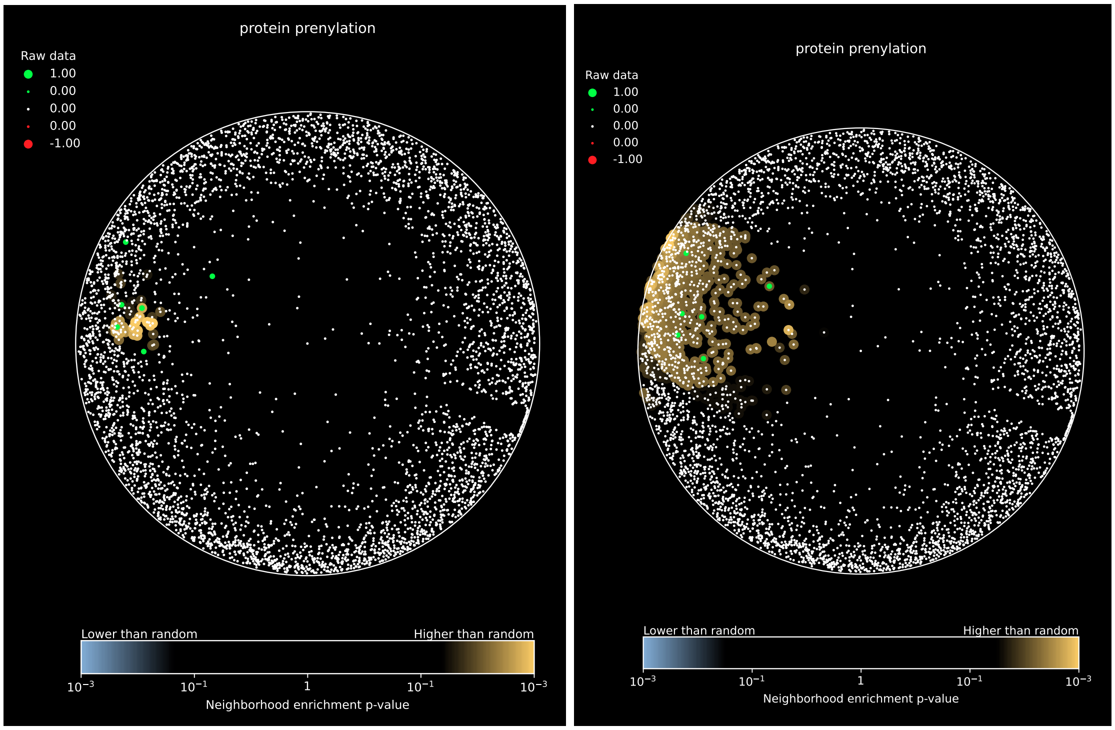

Supplementary Figure 40: **Illustrating the impact of the average neighbourhood size on gene enrichment.** We show for the budding yeast GI network using GraCoal_2 (embedded nodes in white) the enrichment landscape (enriched nodes in yellow) of the 'Protein Prenilation' biological processes (annotated nodes in green), when setting the average number of neighbours of each node to 50 (left) and 1000 (right), respectively.

To measure the impact of the average number of neighbours considered for each node on the functional organisation captured by a given Gracoal in a given network, we compute the pairwise Jaccard similarity between the enriched GO-terms considering various average neighbourhood size between 10 and 100 in increments of 10 and between 100 and 1000 in increments in 100. We show the result for GraCoal_2 applied on the budding yeast GI network in Suppl. Fig. 41. We observe that, in general, the sets of enriched annotations are highly overlapping, with an average Jaccard index of 80% when considering average neighbourhood sizes from 10 to 1000. We also see that beyond an average neighbourhood size of 50, the sets of enriched annotations are even more stable, with an average Jaccard index of 85%.

To show that regardless of the considered type graphlet, the functional organisation captured by a given GraCoal is stable beyond an average neighbourhood size of 50, we repeat this repeat our experiment for all GI networks and GraCoals and summarise the results by measuring the average pairwise Jaccard index when considering average neighbourhoods between 50 and 1000. We present the results in Suppl. Table 18. As the lowest measured average Jaccard is 70% (GraCoal_1 for fission yeast) we conclude that beyond an average neighbourhood size of 50, the average number of neighbours has little impact on the functional organisation captured. This is especially true for $Gracoal_2$, the focus of our in depth analysis of the functional organisation captured by GraCoal, for which we find an average score of 82% over the four species.

We conclude that varying the average number of neighbours beyond 50 does not impact the best performing GraCoal and only has a limited impact on the functional organisation captured by any GraCoal. Additionally, going beyond an average neighbourhood size of 50 leads to large amounts of enriched genes, spread more widely in the embedding space. We believe these results support our choice of focusing on an average number of neighbours of 50, as it we capture as much functional detail as possible while keeping the focus on strongly associated genes.

network	$\widetilde{A}_{G_0}$	$\widetilde{A}_{G_1}$	$\widetilde{A}_{G_2}$	$\widetilde{A}_{G_3}$	$\widetilde{A}_{G_4}$	$\widetilde{A}_{G_5}$	$\widetilde{A}_{G_6}$	$\widetilde{A}_{G_7}$	$\widetilde{A}_{G_8}$	union
Budding yeast	0.72	0.75	0.84	0.84	0.73	0.79	0.83	0.85	0.84	0.89
E. coli	0.79	0.80	0.76	0.78	0.79	0.79	0.80	0.77	0.79	0.80
Fission yeast	0.73	0.70	0.83	0.78	0.60	0.75	0.82	0.76	0.77	0.86

Supplementary Table 18: **The stability of the functional organisation captured by GraCoals in GI networks.** For our four GI networks (rows), for each of our GraCoals (columns), we show the average Jaccard index between the annotations enriched when considering various pairwise combinations of average number of neighbour ranging between 50 and 1000.

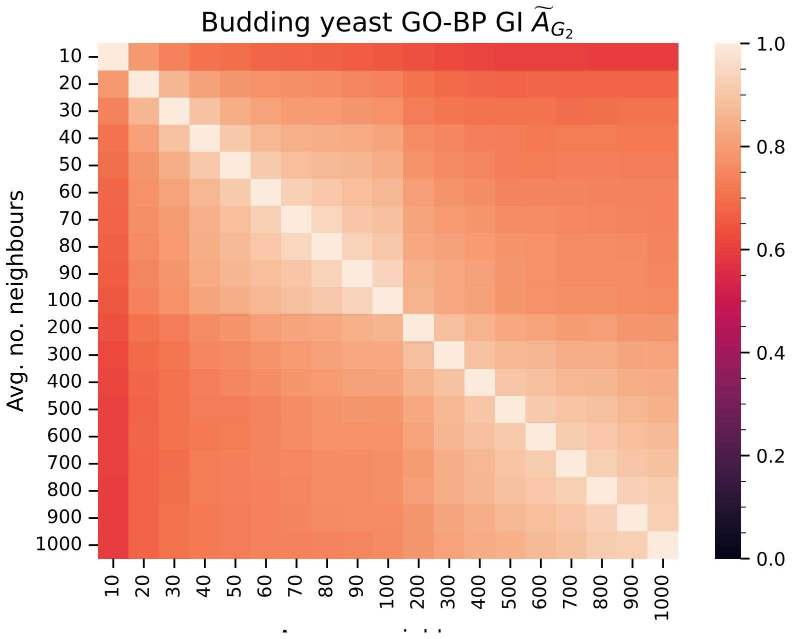

Supplementary Figure 41: **Similarity of the functional organisation captured by Gracoal$_2$ in the budding yeast GI network using GO-BP annotations.** We show the pairwise Jaccard index between the enriched annotations when applying Gracoal$_2$ on the yeast GI network, comparing the enrichments for varying combinations of average number of neighbours (x-axis and y-axis).

References

1. Oughtred, R. *et al.* The BioGRID database: A comprehensive biomedical resource of curated protein, genetic, and chemical interactions. *Protein Science* **30,** 187–200 (2021).
2. Usaj, M. *et al.* TheCellMap. org: a web-accessible database for visualizing and mining the global yeast genetic interaction network. *G3: Genes, genomes, genetics* **7,** 1539–1549 (2017).
3. Costanzo, M. *et al.* The genetic landscape of a cell. *science* **327,** 425–431 (2010).
4. Costanzo, M. *et al.* A global genetic interaction network maps a wiring diagram of cellular function. *Science* **353,** aaf1420 (2016).
5. Windels, S. F. L., Malod-Dognin, N. & Pržulj, N. Graphlet Laplacians for topology-function and topology-disease relationships. eng. *Bioinformatics (Oxford, England)* **35,** 5226–5234 (Dec. 2019).
6. Windels, S. F., Malod-Dognin, N. & Przulj, N. Graphlets correct for the topological information missed by random walks. *arXiv preprint arXiv:2405.14194* (2024).
7. Fruchterman, T. M. & Reingold, E. M. Graph drawing by force-directed placement. *Software: Practice and experience* **21,** 1129–1164 (1991).
8. Windels, S. F., Malod-Dognin, N. & Pržulj, N. Graphlet Laplacians for topology-function and topology-disease relationships. *Bioinformatics* **35,** 5226–5234 (2019).
9. Yaveroğlu, Ö. N. *et al.* Revealing the Hidden Language of Complex Networks. *Scientific Reports* **4** (Apr. 2014).
10. Erdős, P., Rényi, A., *et al.* On the evolution of random graphs. *Publ. Math. Inst. Hung. Acad. Sci* **5,** 17–60 (1960).
11. Newman, M. *Networks* (Oxford University Press, Mar. 2010).
12. Penrose, M. *Random Geometric Graphs* (Oxford University Press, May 2003).
13. Pržulj, N., Kuchaiev, O., Stevanović, A. & Hayes, W. in *Biocomputing 2010* 178–189 (WORLD SCIENTIFIC, Oct. 2009).
14. Barabási, A.-L. & Albert, R. Emergence of Scaling in Random Networks. *Science* **286,** 509–512 (Oct. 1999).
15. Vázquez, A., Flammini, A., Maritan, A. & Vespignani, A. Modeling of Protein Interaction Networks. *Complexus* **1,** 38–44 (Dec. 2002).
16. Muscoloni, A. & Cannistraci, C. V. A nonuniform popularity-similarity optimization (nPSO) model to efficiently generate realistic complex networks with communities. *New Journal of Physics* **20,** 052002 (May 2018).
17. Clauset, A., Newman, M. E. & Moore, C. Finding community structure in very large networks. *Physical review E* **70,** 066111 (2004).
18. Ravasz, E. & Barabási, A.-L. Hierarchical organization in complex networks. *Physical Review E* **67** (Feb. 2003).
19. Anton, C., Taubas, J. V. & Roncero, C. The functional specialization of exomer as a cargo adaptor during the evolution of fungi. *Genetics* **208,** 1483–1498 (2018).
20. Purkanti, R. & Thattai, M. Genome doubling enabled the expansion of yeast vesicle traffic pathways. *Scientific Reports* **12,** 11213 (2022).
21. Maslov, S. & Sneppen, K. Specificity and stability in topology of protein networks. eng. *Science (New York, N.Y.)* **296,** 910–913 (May 2002).
22. Lazareva, O., Baumbach, J., List, M. & Blumenthal, D. B. On the limits of active module identification. *Briefings in Bioinformatics* **22,** bbab066 (2021).
